# Supplementary material for: Evolutionary history of the Arctic flora
Source: Nat Commun. 2023 Jul 18;14:4021. doi: 10.1038/s41467-023-39555-6 (PMC10354081; doi:10.1038/s41467-023-39555-6)
Supplement: Supplementary file 1 — Supplementary Information [file 41467_2023_39555_MOESM1_ESM.pdf]

Supplementary Information for

Evolutionary history of the Arctic flora

Jun Zhang[1,2,3]†, Xiao-Qian Li[1,3,4]†, Huan-Wen Peng[1,3,4], Lisi Hai[1,3,4], Andrey S. Erst[5], Florian Jabbour[6], Rosa del C. Ortiz[7], Fu-Cai Xia[2]*, Pamela S. Soltis[8]*, Douglas E. Soltis[8,9]* & Wei Wang[1,3,4]*

[1] State Key Laboratory of Systematic and Evolutionary Botany, Institute of Botany, Chinese Academy of Sciences, Beijing 100093, China.
[2] Forestry College, Beihua University, Jilin 132013, China.
[3] China National Botanical Garden, Beijing 100093, China.
[4] University of Chinese Academy of Sciences, Beijing 100049, China.
[5] Central Siberian Botanical Garden, Russian Academy of Sciences, Zolotodolinskaya str. 101, Novosibirsk 630090, Russia.
[6] Institut de Systématique, Evolution, Biodiversité (ISYEB), Muséum national d'Histoire naturelle, CNRS, Sorbonne Université, EPHE, Université des Antilles, Paris 75005, France.
[7] Missouri Botanical Garden, 4344 Shaw Blvd, St. Louis, Missouri 63110, USA.
[8] Florida Museum of Natural History, University of Florida, Gainesville, FL 32611, USA.
[9] Department of Biology, University of Florida, Gainesville, FL 32611, USA.
† These authors contributed equally: Jun Zhang, Xiao-Qian Li.

* **Corresponding authors.** email: wangwei1127@ibcas.ac.cn; xfc0707@163.com; psoltis@flmnh.ufl.edu; dsoltis@ufl.edu

This file includes:

Supplementary Note
Legends to Supplementary Figures 1–46
Supplementary Figures 1–46
Supplementary Tables 1–6
Supplementary References

Supplementary Note

Data collection and dated phylogenetic analysis. We selected 32 angiosperm clades that contain Arctic and non-Arctic species and that have sufficient molecular data available to infer a time-calibrated phylogeny covering the major taxonomic and geographical diversity of the clade. The 32 clades contain 3,626 species, of which 548 are distributed in the Arctic and 40 are endemic to the Arctic. These selected taxa belong to 10 orders and 16 families of angiosperms (following APG IV[1]; Supplementary Fig. 1; Supplementary Table 1). This study offers the most comprehensive evolutionary analysis of the Arctic tundra to date. Phylogenetic data using DNA sequences were generated in this study (185 new sequences) or collected from GenBank. GenBank accession numbers are listed in Supplementary Table 7. Dated phylogenetic analysis for each clade is described below.

Arctic *Artemisia* clade (Asteraceae, Asterales)

Two nuclear (ETS and ITS) and two plastid (*mat*K and *rbc*L) DNA regions were used to construct a dated phylogeny for *Artemisia*. Following Tkach et al.[2], we used two fossil calibration points: (i) the crown group age of the *Artemisia*-*Kaschgaria* lineage (mean = 34.0 Ma, SD = 2.0, 95% HPD: 30.7–37.3 Ma), and (ii) the crown age of *Artemisia* (mean = 0.5, SD = 0.5, offset = 31.0 Ma). Two secondary calibration points were also used based on the results of Tkach et al.[2]: (i) the root age (mean = 41.0 Ma, SD = 2.0, 95% HPD: 37.7–44.3), and (ii) the age of the oldest North American clade (mean = 14.0 Ma, SD = 2.0, 95% HPD: 10.7–17.3 Ma).

***Astragalus* (Fabaceae, Fabales)**

Two DNA regions (ITS and *trn*K/*mat*K) were used to construct a dated phylogeny for *Astragalus*. Given that there are no reliable *Astragalus* fossils, we used three secondary calibration points with a normal prior distribution based on the results of Azani et al.[3]: (i) 17.2 Ma for the root of the tree (SD = 3.0, 95% HPD: 12.3–22.1 Ma), (ii) the divergence time of *Oxytropis* and *Astragalus* (mean = 16.1Ma, SD = 2.3, 95% HPD: 12.3–19.9 Ma), and (iii) the crown age of *Astragalus* (mean = 14.4 Ma, SD = 2.0, 95% HPD: 11.1–17.7 Ma).

***Braya* (Brassicaceae, Brassicales)**

Two nuclear (ITS) and plastid (*trn*L) DNA regions were used to construct a dated phylogeny for *Braya*. Based on the results of Chen et al.[4], we used two secondary calibration points: (i) the root age of the tree (mean = 12.0 Ma, SD = 2.0, 95% HPD: 8.7–15.3 Ma), and (ii) the timing of the split of *Lepidostemon* and *Braya* (mean = 8.0 Ma, SD = 2.0, 95% HPD: 4.7–11.3 Ma).

***Cardamine* (Brassicaceae, Brassicales)**

The dataset of Carlsen et al.[5] containing three DNA regions (ITS, *trn*L, and *trn*L-*trn*F) was used to construct a dated phylogeny for *Cardamine*. Based on the results of Guo et al.[6], we used three secondary calibration points: (i) the root age of the tree (mean = 23.0 Ma, SD = 2.0, 95% HPD: 17.0–23.6 Ma), (ii) the crown age of

Barbarea, *Nasturtium* and *Cardamine* (mean = 14.2 Ma, SD = 2.0, 95% HPD: 10.9–17.5 Ma), and (iii) the divergence time of *Capsella* and *Arabidopsis* (mean = 9.9 Ma, SD = 1.5, 95% HPD: 7.4–12.4 Ma).

Arctic *Carex* clade (Cyperaceae, Poales)

Based on the phylogenetic analysis of Martín-Bravo et al.[7], we focused on the clade with Arctic *Carex* species. Two nuclear (ETS and ITS) and one plastid (*mat*K) DNA regions were used to construct a dated phylogeny for this clade. Following Jiménez-Mejías et al.[8], we used eight fossil calibration points: (i) the crown age of *Carex* (SD = 1.0, offset = 33.9 Ma, 95% HPD: 34.1–39.1 Ma), (ii) the crown age of subg. *Vignea* (SD = 1.0, offset = 16.0 Ma, 95% HPD: 16.2–21.2 Ma), (iii) the crown age of sect. *Ammoglochin* (SD = 1.0, offset = 2.6 Ma, 95% HPD: 2.8–7.8 Ma), (iv) the crown age of sect. *Ovales* (SD = 1.0, offset = 2.6 Ma, 95% HPD: 2.8–7.8 Ma), (v) the crown age of subg. *Carex* (SD = 1.0, offset = 23.0 Ma, 95% HPD: 23.2–28.2 Ma), (vi) the crown age of sect. *Paniceae* (SD = 1.0, offset = 5.3 Ma, 95% HPD: 5.5–10.5 Ma), (vii) the crown age of sect. *Phacocystis* (SD = 1.0, offset = 16.0 Ma, 95% HPD: 16.2–21.2 Ma), and (viii) the crown age of sect. *Vesicariae* and *Paludosae* (SD = 1.0, offset = 16.0 Ma, 95% HPD: 16.2–21.2 Ma). The root was also constraint to 54.9 Ma (SD = 3.0, 95% HPD: 50.0–58.8 Ma) based on the result of Martín-Bravo et al.[7].

***Cassiope* (Ericaceae, Ericales)**

The dataset of Hou et al.[9] containing two plastid DNA (*mat*K and *rbc*L) was used to

construct a dated phylogeny for *Cassiope*. Following Hou et al.[9], we used four fossil calibration points: (i) the root age of the tree (mean = 90.0 Ma, SD = 2.5, 95% HPD: 85.9–94.1 Ma), (ii) the crown age of *Vaccinium* (SD = 1.0, offset = 26.5 Ma), (iii) the crown age of *Leucothoe* (SD = 1.0, offset = 13.5 Ma), and (iv) the crown age of *Rhododendron* (SD = 1.0, offset = 54.5 Ma).

***Cerastium* (Caryophyllaceae, Caryophyllales)**

Three plastid DNA regions (*trn*L, *psb*A–*trn*H, and *trn*L-*trn*F) were used to construct a dated phylogeny for *Cerastium*. Following Gizaw et al.[10], we used a fossil calibration point: the root age (mean = 0.0, SD = 1.37, offset = 33.9 Ma, 95% HPD:34.0–43.4 Ma). One paleogeographic event was also used as a calibration point, the age of the earliest opening of the Bering Strait (7.4–4.8 Ma)[11,12], to constrain the divergence time of *Cerastium fragillimum* and *C. biebersteinii*.

***Chrysosplenium* (Saxifragaceae, Saxifragales)**

One plastid DNA region (*mat*K) was used to construct a dated phylogeny for *Chrysosplenium*. Based on the results of Deng et al.[13], we used two secondary calibration points: (i) the root age (mean = 23.14 Ma, SD = 4.5, 95% HPD: 15.7–30.5 Ma), and (ii) the crown age of *Chrysosplenium* (mean =10.84 Ma, SD = 2.0, 95% HPD: 7.6–14.1 Ma).

***Delphinium* (Ranunculaceae, Ranunculales)**

Two DNA regions (ITS and *trn*L-*trn*F) were used to construct a dated phylogeny for *Delphinium*. Based on the results of Jabbour & Renner[14], we used three secondary calibration points: (i) the root age (mean = 39.8 Ma, SD = 2.0, 95% HPD: 36.5–43.1 Ma), (ii) the crown age of *Delphinium* (mean = 23.01 Ma, SD = 2.0, 95% HPD: 19.7–26.3 Ma), and (iii) the crown age of subg. *Consolida* (mean = 19.1 Ma, SD = 2.0, 95% HPD: 15.8–22.4 Ma).

Diapensia **(Diapensiaceae, Ericales)**

The dataset of Hou et al.[15] containing two plastid DNA regions (*mat*K and *rbc*L) was used to construct a dated phylogeny for *Diapensia*. Based on the results of Hou et al.[15], we used three secondary calibration points: (i) the root age (mean = 19.0 Ma, SD = 3.9, 95% HPD: 12.6–25.4 Ma), (ii) the crown age of the clade containing *Shortia*, *Schizocodon* and *Diapensia* (mean = 13.0 Ma, SD = 3.2, 95% HPD: 7.7–18.3 Ma), and (iii) the crown age of *Diapensia* (mean = 8.3 Ma, SD = 2.1, 95% HPD: 4.8–11.8 Ma).

Douglasia-Androsace **clade (Primulaceae, Ericales)**

Two DNA regions (ITS and *trn*L-*trn*F) were used to construct a dated phylogeny for *Douglasia-Androsace* clade. Based on the results of Boucher et al.[16], we used three secondary calibration points: (i) the root age (mean = 36.7Ma, SD = 1.3, 95% HPD: 34.6–38.8 Ma), (ii) the divergence time of *Trientalis* and *Douglasia* (mean = 34.9 Ma, SD = 2.0, 95% HPD: 34.6–35.2 Ma), and (iii) the crown age of *Douglasia*

(mean = 2.7 Ma, SD = 1.5, 95% HPD: 0.2–5.2 Ma).

***Draba* (Brassicaceae, Brassicales)**

Jordon-Thaden et al.[17] built a phylogeny for *Draba* based on ITS and *trn*L-F sequences. We added two plastid DNA regions (*mat*K and *rbc*L) to construct a dated phylogeny for *Draba*. Based on the results of Guo et al.[6], we used two secondary calibration points: (i) the root age (mean = 20.6 Ma, SD = 3.0, 95% HPD: 15.7–25.5 Ma), and (ii) the crown age of Arabideae (mean = 14.6 Ma, SD = 2.0, 95% HPD: 11.3–17.9 Ma).

***Erigeron* (Asteraceae, Asterales)**

Four DNA regions (ITS, *mat*K, *rbc*L, and *trn*L-*trn*F) were used to construct a dated phylogeny for *Erigeron*. Based on the results of Panero & Crozier[18] and Farhani et al.[19], we used five secondary calibration points: (i) the root age (mean = 46.6 Ma, SD = 10.0, 95% HPD: 30.1–63.0 Ma), (ii) the divergence time of *Bellis* and *Erigeron* (mean = 38.7 Ma, SD = 2.1, 95% HPD: 35.4–42.0 Ma), (iii) the divergence time of *Tripolum pannonicum* and *Galatella villosa* (mean = 10.8 Ma, SD = 3.0, 95% HPD: 5.9–15.7 Ma), (iv) the divergence time of *Aster altaicus* and *Psychrogeton alexeenkoi* (mean = 16.2 Ma, SD = 3.0, 95% HPD: 11.3–21.1 Ma), and (v) the crown age of *Erigeron* (mean = 5.8 Ma, SD = 2.0, 95% HPD: 2.5–9.1 Ma).

***Euphrasia* (Orobanchaceae, Lamiales)**

Four DNA regions (ITS, *mat*K, *atp*B-*rbc*L, and *trn*L-*trn*F) were used to construct a dated phylogeny for *Euphrasia*. Based on the results of Gussarova et al.[20], we used three secondary calibration points: (i) the root age (mean = 28.0 Ma, SD = 3.0, 95% HPD: 23.1–32.9 Ma), (ii) the divergence time of *Tozzia alpina* and *Bartsia trixago* (mean = 13.0 Ma, SD = 3.0, 95% HPD: 8.1–17.9 Ma), and (iii) the crown age of *Euphrasia* (mean = 8.0 Ma, SD = 1.0, 95% HPD: 6.4–9.6 Ma).

***Festuca* (Poaceae, Poales)**

Three DNA regions (ITS, *trn*L-F, and *trn*T-L) were used to construct a dated phylogeny for *Festuca*. Based on the results of Inda et al.[21], we used four secondary calibration points: (i) the root age (mean = 44.0 Ma, SD = 2.0, 95% HPD: 40.7–47.3 Ma), (ii) the crown age of Triticeae (mean = 31.2 Ma, SD = 3.5, 95% HPD: 25.0–37.5 Ma), (iii) the crown age of Poeae (mean = 21.6 Ma, SD = 2.5, 95% HPD: 14.2–29.0 Ma), and (iv) the crown age of *Festuca* (mean = 12.9 Ma, SD = 1.0, 95% HPD: 6.1–15.7 Ma).

***Oxytropis* (Fabaceae, Fabales)**

Four DNA regions (ITS, *mat*K, *rbc*L, and *trn*L-F) were used to construct a dated phylogeny for *Oxytropis*. Based on the results of Shavvon et al.[22], we used four secondary calibration points: (i) the root age (mean = 19.7 Ma, SD = 4.0, 95% HPD: 13.1–26.3 Ma), (ii) the divergence time of *Oxytropis* and *Astragalus* (mean = 15.7 Ma, SD = 2.0, 95% HPD: 12.4–19.0 Ma), (iii) the divergence time of *Podlechiella*

vogelii and *Colutea persica* (mean = 15.6 Ma, SD = 2.0, 95% HPD: 12.3–18.9 Ma), and (iv) the crown age of *Oxytropis* (mean = 5.6 Ma, SD = 1.0, 95% HPD: 4.0–7.2 Ma).

Packera **(Asteraceae, Asterales)**

The dataset of Bain & Golden[23] containing ITS1 and ITS2 was used to construct a dated phylogeny for *Packera*. Based on the results of Mandel et al.[24], we used two secondary calibration points: (i) the root age (mean = 20.9 Ma, SD = 2.0, 95% HPD: 17.6–24.2 Ma), and (ii) the crown age of *Packera* (mean = 13.78 Ma, SD = 2.0, 95% HPD: 10.5–17.1 Ma).

Arctic *Papaver* clade (Papaveraceae, Ranunculales)

Four DNA regions (ITS, *mat*K, *rbc*L, and *trn*L-*trn*F) were used to construct a dated phylogeny for the Arctic *Papaver* clade. Based on the results of Peng et al.[25], we used two secondary calibration points: (i) the root age (mean = 33.48 Ma, SD = 2.0, 95% HPD: 30.2–36.8 Ma), and (ii) the crown age of the Arctic *Papaver* clade (mean = 4.12 Ma, SD = 1.0, 95% HPD: 2.5–5.8 Ma).

Parrya **(Brassicaceae, Brassicales)**

The dataset of German et al.[26] containing two DNA regions (ITS and *trn*L-*trn*F) was used to construct a dated phylogeny for *Parrya*. Based on the results of Guo et al.[6], we used three secondary calibration points: (i) the root age (mean = 35.2 Ma, SD =

2.0, 95% HPD: 31.9–38.5 Ma), (ii) the divergence time of *Neuroloma* and *Parrya* (mean = 16.6 Ma, SD = 2.0, 95% HPD: 13.3–19.9 Ma), and (iii) the divergence time of *Matthiola* and *Solms-laubachia* (mean = 17.3 Ma, SD = 2.0, 95% HPD: 14.0–20.6 Ma).

***Pedicularis* (Orobanchaceae, Lamiales)**

Three DNA regions (ITS, *mat*K/*trn*K, and *rbc*L) were used to construct a dated phylogeny for *Pedicularis*. Based on the results of Yu et al.[27], we used three secondary calibration points: (i) the root age (mean = 47.9 Ma, SD = 5.0, 95% HPD: 39.7–56.1 Ma), (ii) the crown age of Pedicularideae (mean = 35.4 Ma, SD = 6.5, 95% HPD: 24.7–46.1 Ma), and (iii) the crown age of Cymbarieae (mean = 37.0 Ma, SD = 6.0, 95% HPD: 26.3–47.7 Ma).

***Plantago* (Plantaginaceae, Lamiales)**

Based on the phylogenetic analysis of Rønsted et al.[28], we focused on the clade with Arctic *Plantago* species. Three DNA regions (ITS, *rbc*L, and *trn*L-*trn*F) were used to construct a dated phylogeny for this clade. Following the results of Rønsted et al.[28], we used two secondary calibration points: (i) the root age (mean = 7.1 Ma, SD = 2.0, 95% HPD: 3.8–10.4 Ma), and (ii) the crown age of *Plantago* (mean = 5.47 Ma, SD = 1.0, 95% HPD: 3.8–7.1 Ma).

***Pleuropogon* (Poaceae, Poales)**

Three DNA regions (ITS, *rbc*L, and *trn*K) were used to construct a dated phylogeny for *Pleuropogon*. Based on the results of Bouchenak-Khelladi et al.[29], we used two secondary calibration points: (i) the root age (mean = 44.0 Ma, SD = 2.0, 95% HPD: 40.7–47.3 Ma), and (ii) the stem age of *Dryopoa* (mean =21.6 Ma, SD = 2.0, 95% HPD: 18.3–24.9 Ma).

***Poa* (Poaceae, Poales)**

Two nuclear (ETS and ITS) and two plastid (*mat*K and *trn*T-*trn*F) DNA regions were used to construct a dated phylogeny for *Poa*. Based on the results of Bouchenak-Khelladi et al.[29], we used five secondary calibration points: (i) the root age (mean = 44.0 Ma, SD = 2.0, 95% HPD: 40.7–47.3 Ma), (ii) the crown age of Triticeae (mean = 31.2 Ma, SD = 2.0, 95% HPD: 25.0–37.5 Ma), (iii) the crown age of Poeae (mean = 21.6 Ma, SD = 4.6, 95% HPD: 14.2–29.0 Ma), (iv) the crown age of *Festuca* (mean = 10.6 Ma, SD = 2.0, 95% HPD: 6.1–15.7 Ma), and (v) the crown age of *Puccinellia* (mean = 3.6 Ma, SD = 1.0, 95% HPD: 1.5–5.9 Ma).

***Potentilla* (Brassicaceae, Brassicales)**

Six DNA regions (ITS, *mat*K, *rbc*L, *trn*C-*ycf*6, *trn*L-*trn*F, and *trn*S-*ycf*9) were used to construct a dated phylogeny for *Potentilla*. Based on the results of Dobeš & Paule[30], we used five secondary calibration points: (i) the root age (mean = 76.0 Ma, SD = 2.0, 95% HPD: 72.7–79.3 Ma), (ii) the divergence time of *Fragaria* and *Potentilla* (mean = 49.3 Ma, SD = 2.0, 95% HPD: 46.0–52.6 Ma), (iii) the crown age

of *Drymocallis* (mean = 4.5 Ma, SD = 1.0, 95% HPD: 2.9–6.1 Ma), (iv) the stem age of *Horkelia* (mean = 3.7 Ma, SD = 1.0, 95% HPD: 2.1–5.3 Ma), and (v) the split time of *Dasiphora* and *Drymocallis* (mean = 10.9 Ma, SD = 2.0, 95% HPD: 7.6–14.2 Ma).

***Primula* (Primulaceae, Ericales)**

The dataset of Mast et al.[31] containing four plastid DNA regions (*mat*K, *rpl*16, *trn*L, and *trn*L-*trn*F) was used to construct a dated phylogeny for *Primula*. Based on the results of de Vos et al.[32], we used four secondary calibration points: (i) the root age (mean = 39.9 Ma, SD = 11.5, 95% HPD: 21.0–58.8 Ma), (ii) the crown age of *Androsace* (mean = 24.5 Ma, SD = 8.0, 95% HPD: 11.3–37.7 Ma), (iii) the stem age of *Bryocarpum* (mean = 14.0 Ma, SD = 5.0, 95% HPD: 5.8–22.2 Ma), and (iv) the split time of *Vitaliana* and *Primula* (mean = 22.0 Ma, SD = 6.0, 95% HPD: 12.1–31.9 Ma).

***Puccinellia* (Poaceae, Poales)**

Four DNA regions (ITS, *mat*K, *rbc*L, and *trn*L-*trn*F) were used to construct a dated phylogeny for *Puccinellia.* Based on the results of Bouchenak-Khelladi et al.[29], we used three secondary calibration points: (i) the root age (mean = 44.0 Ma, SD = 2.0, 95% HPD: 40.7–47.3 Ma), (ii) the crown age of Triticeae (mean = 31.2 Ma, SD = 2.0, 95% HPD: 25.0–37.5 Ma), and (iii) the crown age of Poeae (mean = 21.6 Ma, SD = 4.6, 95% HPD: 14.2–29.0 Ma).

***Ranunculus* (Ranunculaceae, Ranunculales)**

Six DNA regions (ITS, *mat*K, *rbc*L, *atp*B-*rbc*L, *psb*J-*pet*A, and *trn*L-*trn*F) were used to construct a dated phylogeny for *Ranunculus*. Based on the results of Emadzade & Hörandl[33], we used six secondary calibration points: (i) the root age (mean = 46.5 Ma, SD = 3.0, 95% HPD: 41.6–51.4 Ma), (ii) the divergence time of *Psychrophila* and *Ranunculus* (mean = 45.7 Ma, SD = 3.0, 95% HPD: 40.7–47.3 Ma), (iii) the crown age of *Ranunculus* (mean = 39.1 Ma, SD = 3.0, 95% HPD: 34.2–44.0 Ma), (iv) the divergence time of *Beckwithia* and *Halerpestes* (mean = 34.8 Ma, SD = 3.0, 95% HPD: 29.9–39.7 Ma), (v) the divergence time of *Kumlienia* and *Halerpestes* (mean = 28.1 Ma, SD = 3.0, 95% HPD: 23.2–33.0 Ma), and (vi) the crown age of *Oxygraphis* (mean = 23.0 Ma, SD = 3.0, 95% HPD: 18.1–28.0 Ma).

***Rumex* (Polygonaceae, Caryophyllales)**

Four DNA regions (ITS, *mat*K, *rbc*L, and *trn*L-*trn*F) were used to construct a dated phylogeny for *Rumex*. Following Schuster et al.[34], we used a fossil calibration point: the crown age of *Calligonum* (mean = 5.3 Ma, offset = 2.6, 95% HPD: 2.9–18.5 Ma). Three secondary calibration points were also used based on the results of Schuster et al.[31]: (i) the root age (mean = 65.0 Ma, SD = 3.0, 95% HPD: 60.1–69.9 Ma), (ii) the divergence time of *Rumex* and *Oxyria* (mean = 20.8 Ma, SD = 2.0, 95% HPD: 17.5–24.1 Ma), and (iii) the divergence time of *Koenigia* and *Polygonum* (mean = 30.7 Ma, SD = 2.0, 95% HPD: 27.4–34.0 Ma).

***Saxifraga* (Saxifragaceae, Saxifragales)**

Based on the phylogenetic analysis of Ebersbach et al.[35], we focused on the clade with Arctic *Saxifraga* species. Three DNA regions (ITS, *mat*K, and *trn*L-*trn*F) were used to construct a dated phylogeny for *Saxifraga*. Following Ebersbach et al.[35], we used four fossil calibration points: (i) the stem age of *Ribes* (mean = 1.5, SD = 1.0, offset = 48.9, 95% HPD: 49.8–72.1 Ma), (ii) the divergence time of *Itea* and *Pterostemon* (mean = 1.5, SD = 1.0, offset = 49.0, 95% HPD: 49.9–72.2 Ma), (iii) the crown age of *Ribes* (mean = 1.5, SD = 1.0, offset = 14.5, 95% HPD: 15.4–37.7 Ma), and (iv) the crown age of *Saxifraga* (mean = 61.8 Ma, SD = 6.0, 95% HPD: 51.9–71.7 Ma). The root was also constrained to 89.0 Ma (SD = 1.0, 95% HPD: 89.9–112.2 Ma) based on the result of Ebersbach et al.[35].

***Silene* (Caryophyllaceae, Caryophyllales)**

Four DNA regions (ITS, *rbc*L, *rps*16, and *trn*L-*trn*F) were used to construct a dated phylogeny for *Silene*. Based on the results of Sloan et al.[36], we used two secondary calibration points: (i) the root age (mean = 14.2 Ma, SD = 3.0, 95% HPD: 9.3–19.1 Ma), and (ii) the crown age of clade containing *Heliosperma*, *Viscaria,* and *Atocion* (mean = 10.6 Ma, SD = 2.5, 95% HPD: 6.5–14.7 Ma).

***Smelowskia* (Brassicaceae, Brassicales)**

The dataset of Carlsen et al.[37] containing six DNA regions (ITS, *rbc*L, *rpa*2, *rps*16,

*rpl*20-*rps*12, and *trn*H-*psb*A) was used to construct a dated phylogeny for *Smelowskia*. Based on the results of Couvreur et al.[38], the split time of *Smelowskia* and *Sophiopsis* was constraint to 9.9 Ma (SD = 3.0, 95% HPD: 6.6–13.2 Ma).

***Symphyotrichum* (Asteraceae, Asterales)**

The dataset of Vaezi & Brouillet[39] containing two nuclear DNA regions (GAPDH and ITS) was used to construct a dated phylogeny for *Symphyotrichum*. Based on the results of Farhani et al.[19], we used two secondary calibration points: (i) the root age (mean = 17.75 Ma, SD = 2.0, 95% HPD: 14.5–21.1 Ma), and (ii) the divergence time of *Erigeron* and *Symphyotrichum* (mean = 16.19 Ma, SD = 2.0, 95% HPD: 12.9–19.5 Ma).

Geographic range and habitat data. Geographic range and habitat information were mainly assembled from the following sources:

Plants of the World Online (https://powo.science.kew.org/)

Global Biodiversity Information Facility (https://www.gbif.org/)

The Flora of Svalbard (https://svalbardflora.no/oldsite/)

New York Flora Atlas (https://newyork.plantatlas.usf.edu/)

Arctic Flora of Canada and Alaska (https://arcticplants.myspecies.info/)

Flora of North America (http://beta.floranorthamerica.org/)

Flora of China (http://www.iplant.cn/)

Panarctic Flora (http://panarcticflora.org/)

Legends to Supplementary Figures 1–46

Supplementary Figure 1 | Distribution of the selected 32 clades in the angiosperm order-level tree of life. The phylogenetic tree is modified from APG IV[1]. The orders and families to which the selected 32 clades belong are in bold.

Supplementary Figure 2 | Timetree for Arctic *Artemisia* clade. Gray bars represent 95% highest posterior density intervals. Arctic species are indicated by asterisks, with endemic species printed in red, and Source data are provided as a Source Data file.

Supplementary Figure 3 | Timetree for *Astragalus*. Gray bars represent 95% highest posterior density intervals. Arctic species are indicated by asterisks, with endemic species printed in red, and Source data are provided as a Source Data file.

Supplementary Figure 4 | Timetree for *Cardamine*. Gray bars represent 95% highest posterior density intervals. Arctic species are indicated by asterisks, with endemic species printed in red, and Source data are provided as a Source Data file.

Supplementary Figure 5 | Timetree for Arctic *Carex* clade. Gray bars represent 95% highest posterior density intervals. Arctic species are indicated by asterisks, with endemic species printed in red, and Source data are provided as a Source Data file.

Supplementary Figure 6 | Timetree for *Cerastium*. Gray bars represent 95% highest posterior density intervals. Arctic species are indicated by asterisks, with endemic species printed in red, and Source data are provided as a Source Data file.

Supplementary Figure 7 | Timetree for *Delphinium*. Gray bars represent 95%

highest posterior density intervals. Arctic species are indicated by asterisks, with endemic species printed in red, and Source data are provided as a Source Data file.

Supplementary Figure 8 | Timetree for *Draba*. Gray bars represent 95% highest posterior density intervals. Arctic species are indicated by asterisks, with endemic species printed in red, and Source data are provided as a Source Data file.

Supplementary Figure 9 | Timetree for *Erigeron*. Gray bars represent 95% highest posterior density intervals. Arctic species are indicated by asterisks, with endemic species printed in red, and Source data are provided as a Source Data file.

Supplementary Figure 10 | Timetrees for *Euphrasia*. Gray bars represent 95% highest posterior density intervals. Arctic species are indicated by asterisks, with endemic species printed in red, and Source data are provided as a Source Data file.

Supplementary Figure 11 | Timetree for *Festuca*. Gray bars represent 95% highest posterior density intervals. Arctic species are indicated by asterisks, with endemic species printed in red, and Source data are provided as a Source Data file.

Supplementary Figure 12 | Timetrees for *Oxytropis*. Gray bars represent 95% highest posterior density intervals. Arctic species are indicated by asterisks, with endemic species printed in red, and Source data are provided as a Source Data file.

Supplementary Figure 13 | Timetree for *Parrya*. Gray bars represent 95% highest posterior density intervals. Arctic species are indicated by asterisks, with endemic species printed in red, and Source data are provided as a Source Data file.

Supplementary Figure 14 | Timetree for *Pedicularis*. Gray bars represent 95% highest posterior density intervals. Arctic species are indicated by asterisks, with

endemic species printed in red, and Source data are provided as a Source Data file.

Supplementary Figure 15 | Timetree for *Plantago*. Gray bars represent 95% highest posterior density intervals. Arctic species are indicated by asterisks, with endemic species printed in red, and Source data are provided as a Source Data file.

Supplementary Figure 16 | Timetree for *Poa*. Gray bars represent 95% highest posterior density intervals. Arctic species are indicated by asterisks, with endemic species printed in red, and Source data are provided as a Source Data file.

Supplementary Figure 17 | Timetree for *Potentilla*. Gray bars represent 95% highest posterior density intervals. Arctic species are indicated by asterisks, with endemic species printed in red, and Source data are provided as a Source Data file.

Supplementary Figure 18 | Timetree for *Primula*. Gray bars represent 95% highest posterior density intervals. Arctic species are indicated by asterisks, with endemic species printed in red, and Source data are provided as a Source Data file.

Supplementary Figure 19 | Timetree for *Puccinellia*. Gray bars represent 95% highest posterior density intervals. Arctic species are indicated by asterisks, with endemic species printed in red, and Source data are provided as a Source Data file.

Supplementary Figure 20 | Timetree for *Ranunculus*. Gray bars represent 95% highest posterior density intervals. Arctic species are indicated by asterisks, with endemic species printed in red, and Source data are provided as a Source Data file.

Supplementary Figure 3 | Timetrees for *Astragalus*. Gray bars represent 95% highest posterior density intervals. Arctic species are indicated by asterisks, with endemic species printed in red, and Source data are provided as a Source Data file.

Supplementary Figure 21 | Timetree for *Rumex*. Gray bars represent 95% highest posterior density intervals. Arctic species are indicated by asterisks, with endemic species printed in red, and Source data are provided as a Source Data file.

Supplementary Figure 22 | Timetree for *Saxifraga*. Gray bars represent 95% highest posterior density intervals. Arctic species are indicated by asterisks, with endemic species printed in red, and Source data are provided as a Source Data file.

Supplementary Figure 23 | Timetrees for *Silene*. Gray bars represent 95% highest posterior density intervals. Arctic species are indicated by asterisks, with endemic species printed in red, and Source data are provided as a Source Data file.

Supplementary Figure 24 | Timetrees for *Cassiope*, *Symphyotrichum* , *Braya*, and *Diapensia*. Gray bars represent 95% highest posterior density intervals. Arctic species are indicated by asterisks, with endemic species printed in red, and Source data are provided as a Source Data file.

Supplementary Figure 25 | Timetrees for *Douglasia-Androsace* clade and *Smelowskia*. Gray bars represent 95% highest posterior density intervals. Arctic species are indicated by asterisks, with endemic species printed in red, and Source data are provided as a Source Data file.

Supplementary Figure 26 | Timetrees for *Pleuropogon* and *Packera*. Gray bars represent 95% highest posterior density intervals. Arctic species are indicated by asterisks, with endemic species printed in red, and Source data are provided as a Source Data file.

Supplementary Figure 27 | Timetrees for Arctic *Papaver* clade and

***Chrysosplenium*.** Gray bars represent 95% highest posterior density intervals. Arctic species are indicated by asterisks, with endemic species printed in red, and Source data are provided as a Source Data file.

Supplementary Figure 28 | Identification of dispersal events and *in situ* diversification events at the species level. Example of a phylogenetic tree showing the ancestral areas depicted for each node. Red represents a dispersal event into the Arctic, and blue represents an Arctic *in situ* diversification event.

Supplementary Figure 29 | Ancestral range reconstructions of Arctic *Carex* clade, *Ranunculus*, *Saxifraga*, and *Diapensia* with two defined regions. Pie charts on each node show the relative probabilities of alternative ancestral distributions derived from the Dispersal-Extinction-Cladogenesis (DEC) model in BioGeoBEARS. Arctic endemic species are in red, and Source data are provided as a Source Data file.

Supplementary Figure 30 | Ancestral range reconstructions of *Silene*, *Draba*, *Erigeron*, and *Braya* with two defined regions. Pie charts on each node show the relative probabilities of alternative ancestral distributions derived from the Dispersal-Extinction-Cladogenesis (DEC) model in BioGeoBEARS. Arctic endemic species are in red, and Source data are provided as a Source Data file.

Supplementary Figure 31 | Ancestral range reconstructions of *Oxytropis, Poa,* Arctic *Artemisia* clade, *Euphrasia*, *Puccinellia*, *Festuca*, and *Plantago* with two defined regions. Pie charts on each node show the relative probabilities of alternative ancestral distributions derived from the

Dispersal-Extinction-Cladogenesis (DEC) model in BioGeoBEARS. Arctic endemic species are in red, and Source data are provided as a Source Data file.

Supplementary Figure 32 | Ancestral range reconstructions of *Cardamine*, Cassiope, *Parrya*, *Delphinium*, and *Douglasia-Androsace* clade with two defined regions. Pie charts on each node show the relative probabilities of alternative ancestral distributions derived from the Dispersal-Extinction-Cladogenesis (DEC) model in BioGeoBEARS. Arctic endemic species are in red, and Source data are provided as a Source Data file.

Supplementary Figure 33 | Ancestral range reconstructions of *Cerastium*, *Chrysosplenium*, *Packera*, and *Symphyotrichum* with two defined regions. Pie charts on each node show the relative probabilities of alternative ancestral distributions derived from the Dispersal-Extinction-Cladogenesis (DEC) model in BioGeoBEARS. Arctic endemic species are in red, and Source data are provided as a Source Data file.

Supplementary Figure 34 | Ancestral range reconstructions of *Potentilla*, *Smelowskia*, Arctic *Papaver* clade, and *Pleuropogon* with two defined regions. Pie charts on each node show the relative probabilities of alternative ancestral distributions derived from the Dispersal-Extinction-Cladogenesis (DEC) model in BioGeoBEARS. Arctic endemic species are in red, and Source data are provided as a Source Data file.

Supplementary Figure 35 | Ancestral range reconstructions of *Pedicularis*, *Astragalus*, *Primula*, and *Rumex* with two defined regions. Pie charts on each

node show the relative probabilities of alternative ancestral distributions derived from the Dispersal-Extinction-Cladogenesis (DEC) model in BioGeoBEARS. Arctic endemic species are in red, and Source data are provided as a Source Data file.

Supplementary Figure 36 | Results of timepoints when the rate changed with 3 breakpoints. a Dispersal events. **b** *In situ* diversification events. MDisE = maximal number of observed dispersal events per Ma. MDivE = maximal number of observed *in situ* diversification events per Ma. Source data are provided as a Source Data file.

Supplementary Figure 37 | Ancestral range reconstructions of Arctic *Carex* clade, *Ranunculus*, *Chrysosplenium*, *Symphyotrichum*, and *Cerastium* with the thirteen defined regions. Pie charts on each node show the relative probabilities of alternative ancestral distributions derived from the Dispersal-Extinction-Cladogenesis (DEC) model in BioGeoBEARS. Arctic endemic species are in red, and Source data are provided as a Source Data file.

Supplementary Figure 38 | Ancestral range reconstructions of *Silene*, *Draba*, *Erigeron*, and *Braya* with the thirteen defined regions. Pie charts on each node show the relative probabilities of alternative ancestral distributions derived from the Dispersal-Extinction-Cladogenesis (DEC) model in BioGeoBEARS. Arctic endemic species are in red, and Source data are provided as a Source Data file.

Supplementary Figure 39 | Ancestral range reconstructions of *Oxytropis*, *Euphrasia*, Arctic *Artemisia* clade, *Festuca*, and *Puccinellia* with the thirteen defined regions. Pie charts on each node show the relative probabilities of alternative ancestral distributions derived from the

Dispersal-Extinction-Cladogenesis (DEC) model in BioGeoBEARS. Arctic endemic species are in red, and Source data are provided as a Source Data file.

Supplementary Figure 40 | Ancestral range reconstructions of *Parrya*, *Douglasia-Androsace* clade, *Smelowskia*, and Arctic *Papaver* clade with the thirteen defined regions. Pie charts on each node show the relative probabilities of alternative ancestral distributions derived from the Dispersal-Extinction-Cladogenesis (DEC) model in BioGeoBEARS. Arctic endemic species are in red, and Source data are provided as a Source Data file.

Supplementary Figure 41 | Ancestral range reconstructions of *Primula*, *Poa*, *Cardamine*, *Pleuropogon*, and *Potentilla* with the thirteen defined regions. Pie charts on each node show the relative probabilities of alternative ancestral distributions derived from the Dispersal-Extinction-Cladogenesis (DEC) model in BioGeoBEARS. Arctic endemic species are in red, and Source data are provided as a Source Data file.

Supplementary Figure 42 | Ancestral habitat reconstructions of Arctic *Carex* clade, *Ranunculus*, *Chrysosplenium*, *Symphyotrichum*, and *Cerastium*. Pie charts on each node show marginal probabilities for each alternative ancestral habitat type derived from the Bayesian binary Markov chain Monte Carlo (BBM) method in RASP. Arctic endemic species are in red, and Source data are provided as a Source Data file.

Supplementary Figure 43 | Ancestral habitat reconstructions of *Silene*, *Draba*, *Erigeron*, and *Braya*. Pie charts on each node show marginal probabilities for each

alternative ancestral habitat type derived from the Bayesian binary Markov chain Monte Carlo (BBM) method in RASP. Arctic endemic species are in red, and Source data are provided as a Source Data file.

Supplementary Figure 44 | Ancestral habitat reconstructions of *Oxytropis*, *Euphrasia*, Arctic *Artemisia* clade, *Festuca*, and *Puccinellia*. Pie charts on each node show marginal probabilities for each alternative ancestral habitat type derived from the Bayesian binary Markov chain Monte Carlo (BBM) method in RASP. Arctic endemic species are in red, and Source data are provided as a Source Data file.

Supplementary Figure 45 | Ancestral habitat reconstructions of *Parrya*, *Douglasia-Androsace* clade, *Smelowskia*, and Arctic *Papaver* clade. Pie charts on each node show marginal probabilities for each alternative ancestral habitat type derived from the Bayesian binary Markov chain Monte Carlo (BBM) method in RASP. Arctic endemic species are in red, and Source data are provided as a Source Data file.

Supplementary Figure 46 | Ancestral habitat reconstructions of *Primula*, *Poa*, *Cardamine*, *Pleuropogon*, and *Potentilla*. Pie charts on each node show marginal probabilities for each alternative ancestral habitat type derived from the Bayesian binary Markov chain Monte Carlo (BBM) method in RASP. Arctic endemic species are in red, and Source data are provided as a Source Data file.

Supplementary Figure 1

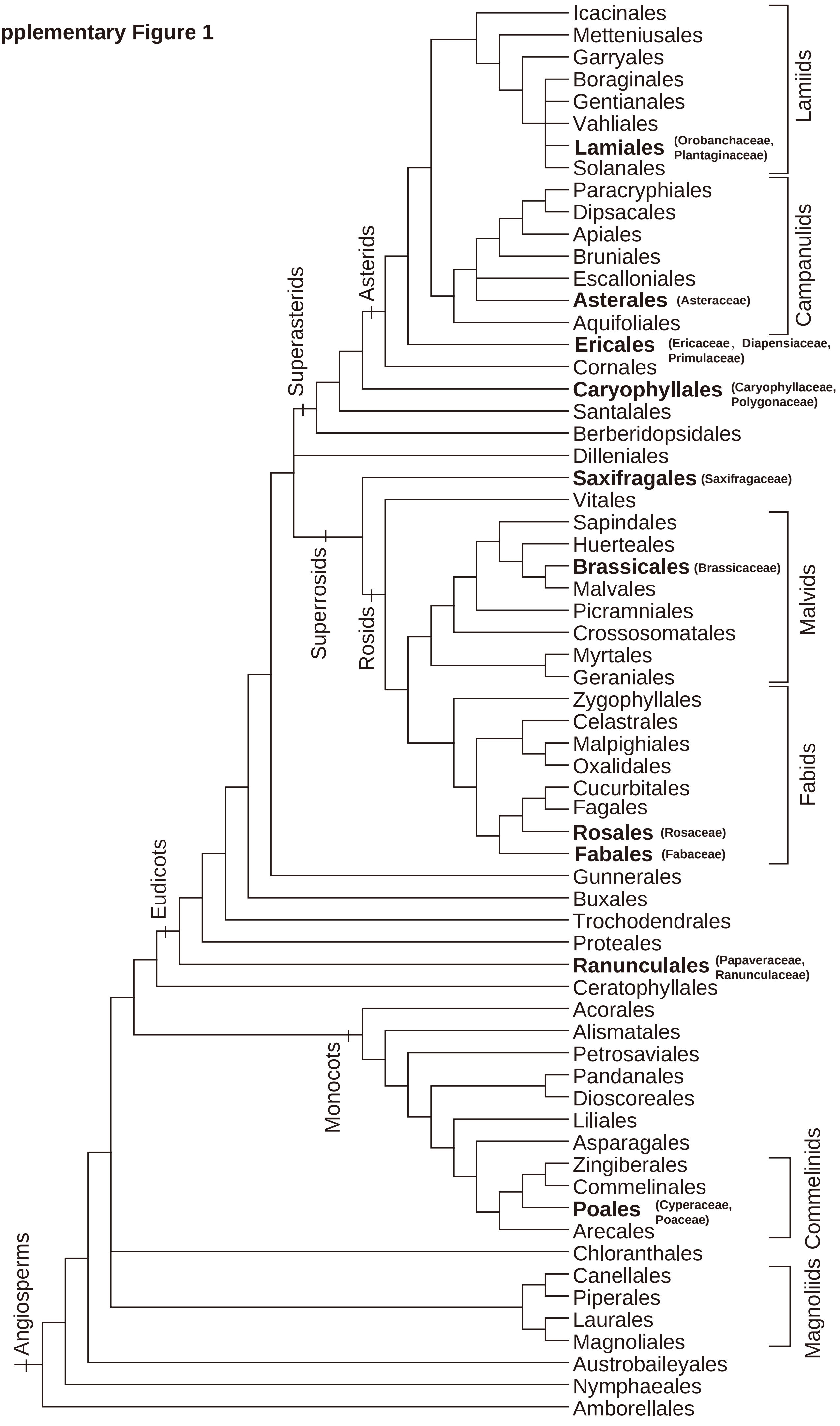

Supplementary Figure 2

Arctic *Artemisia* clade (Asteraceae)

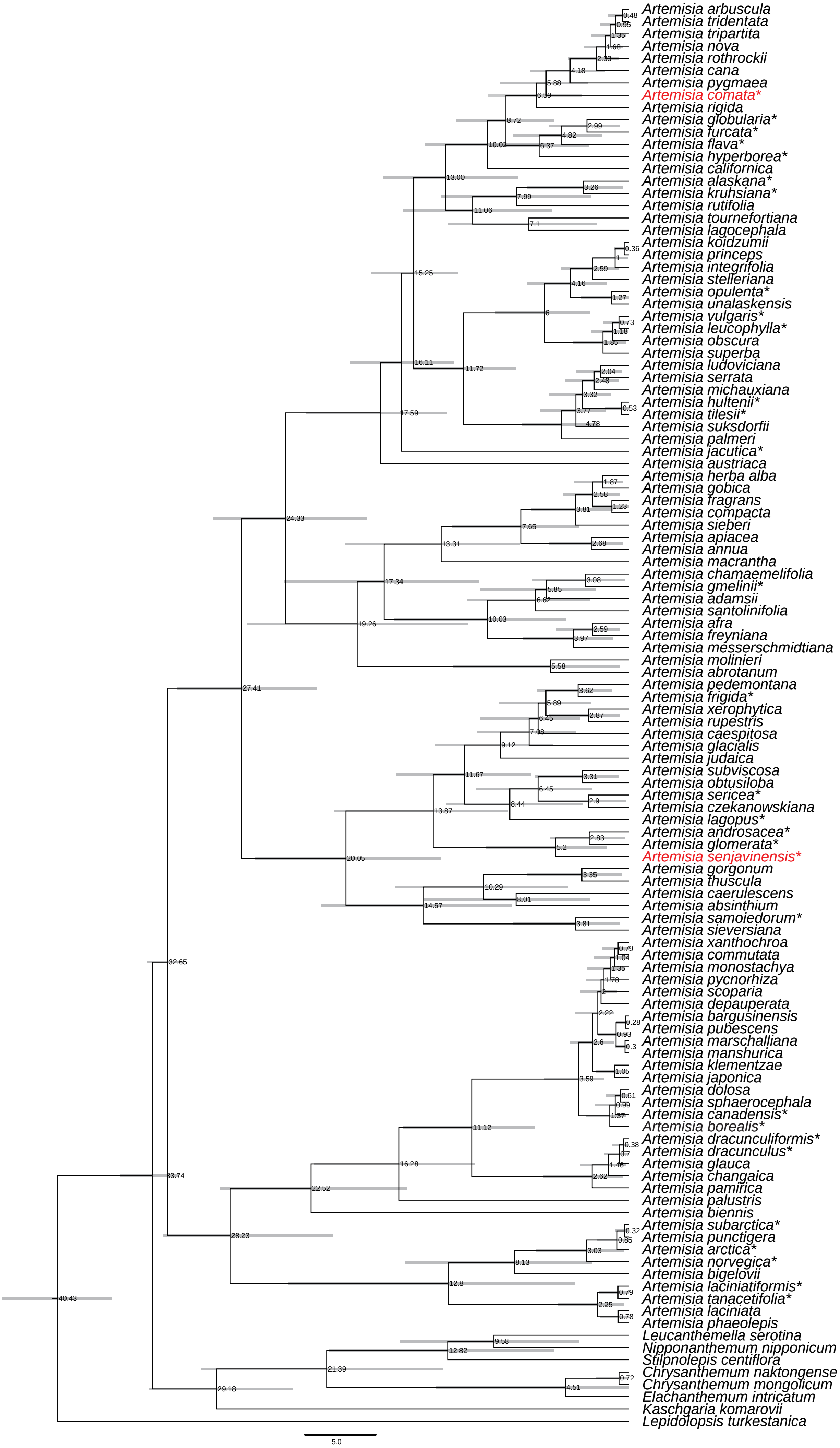

Supplementary Figure 3 *Astragalus* (Fabaceae)

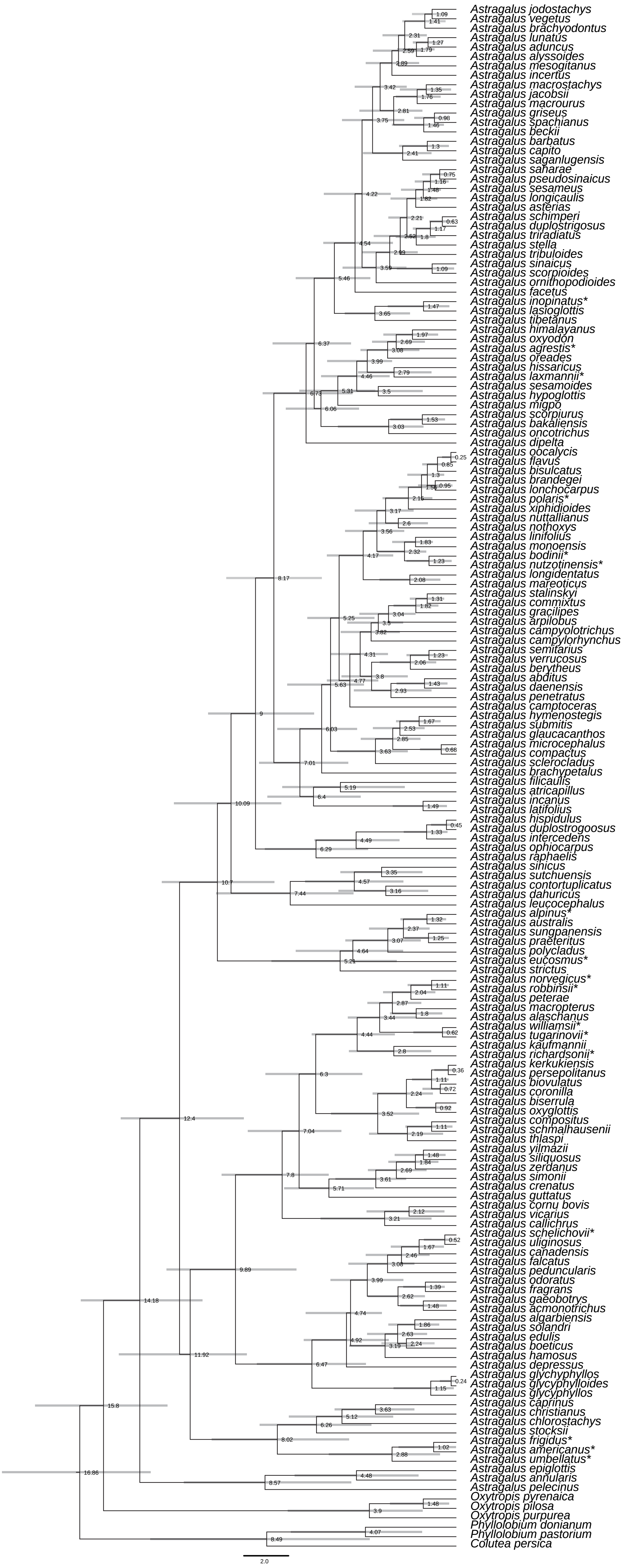

Supplementary Figure 4

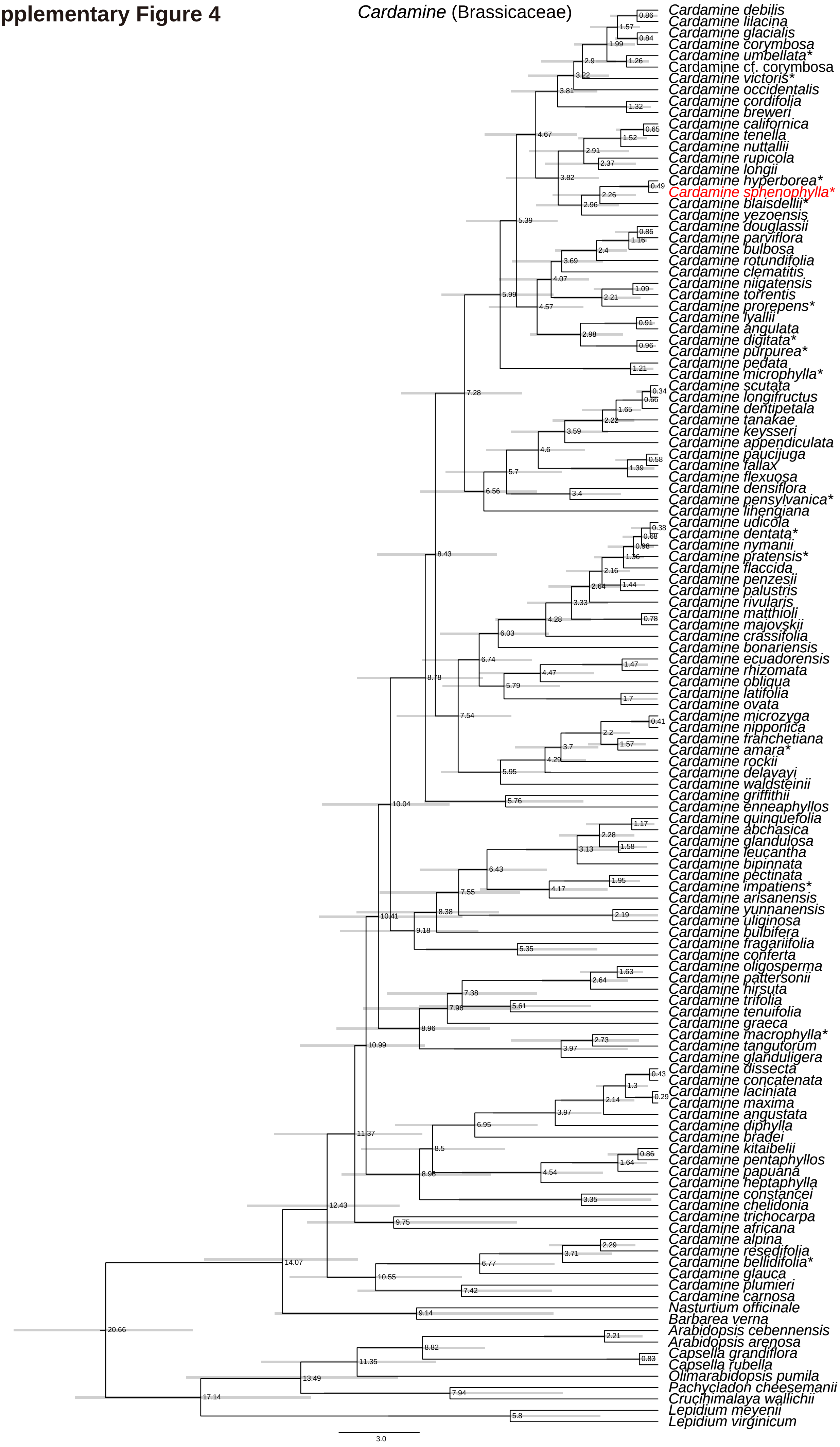

Supplementary Figure 5 Arctic *Carex* clade (Cyperaceae)

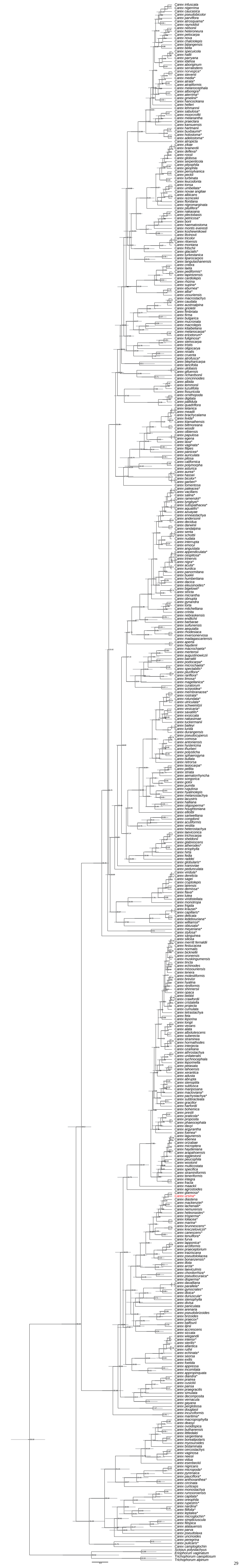

Supplementary Figure 6 *Cerastium* (Caryophyllaceae)

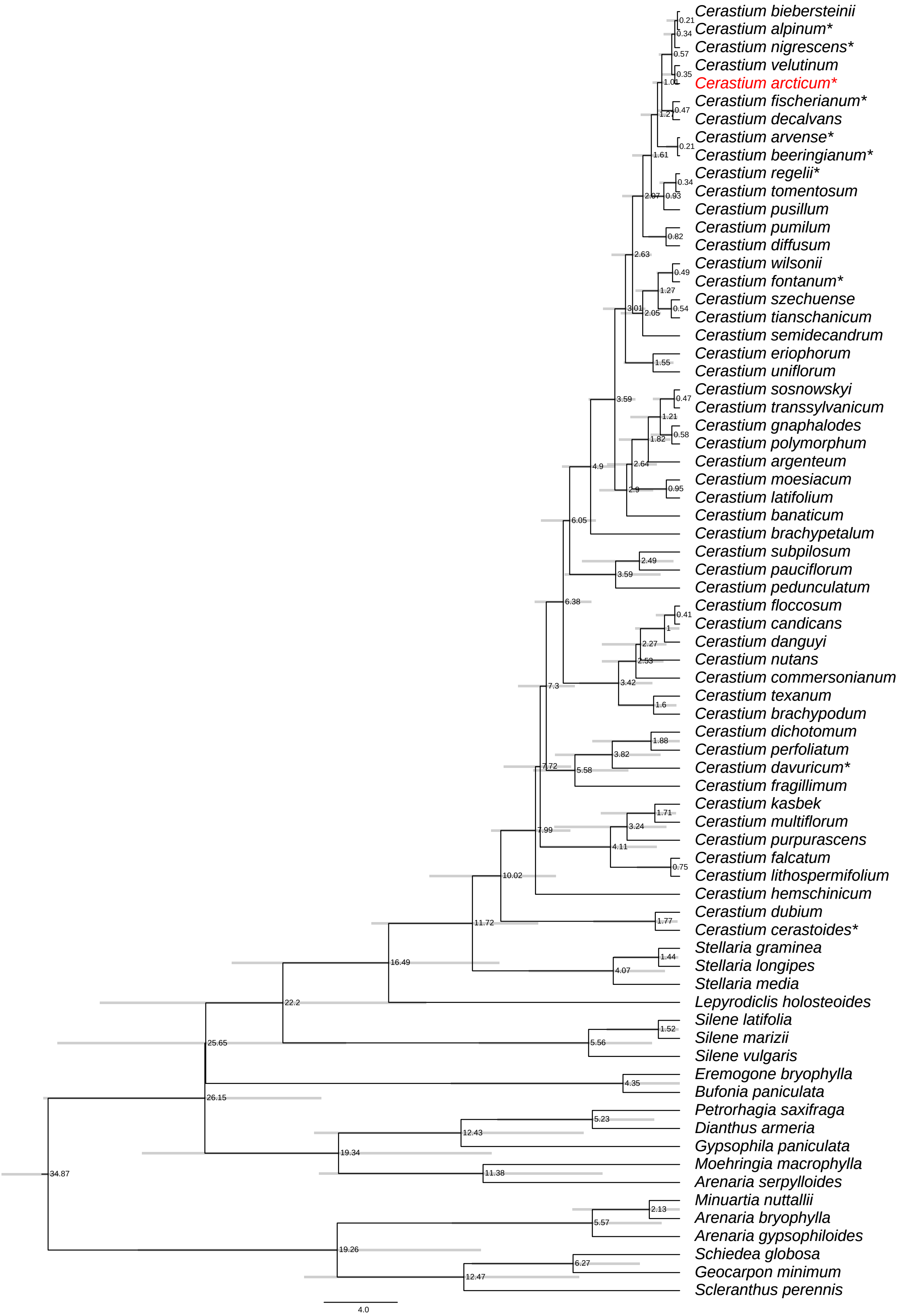

Supplementary Figure 7

Delphinium (Ranunculaceae)

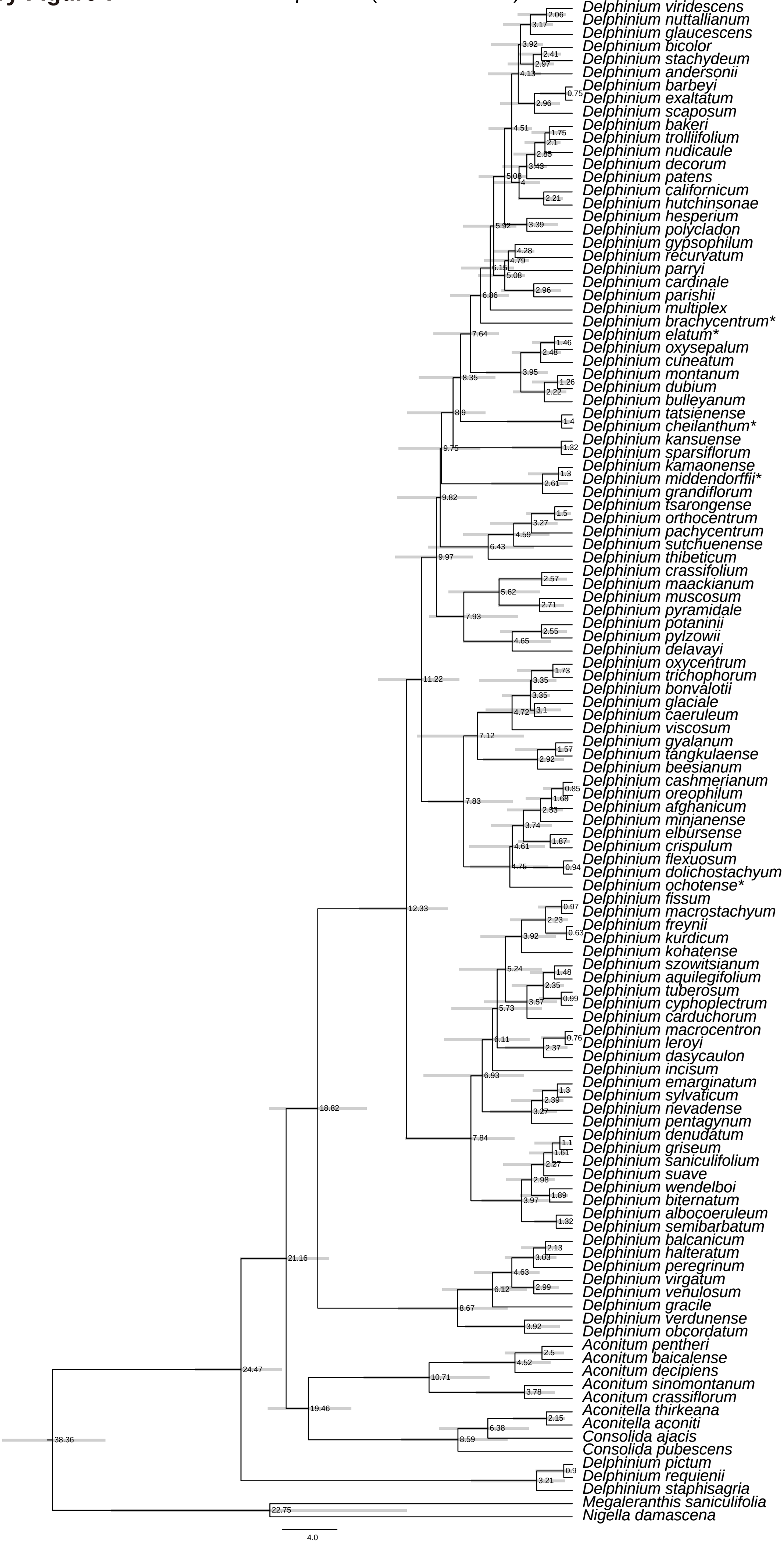

Supplementary Figure 8

Draba (Brassicaceae)

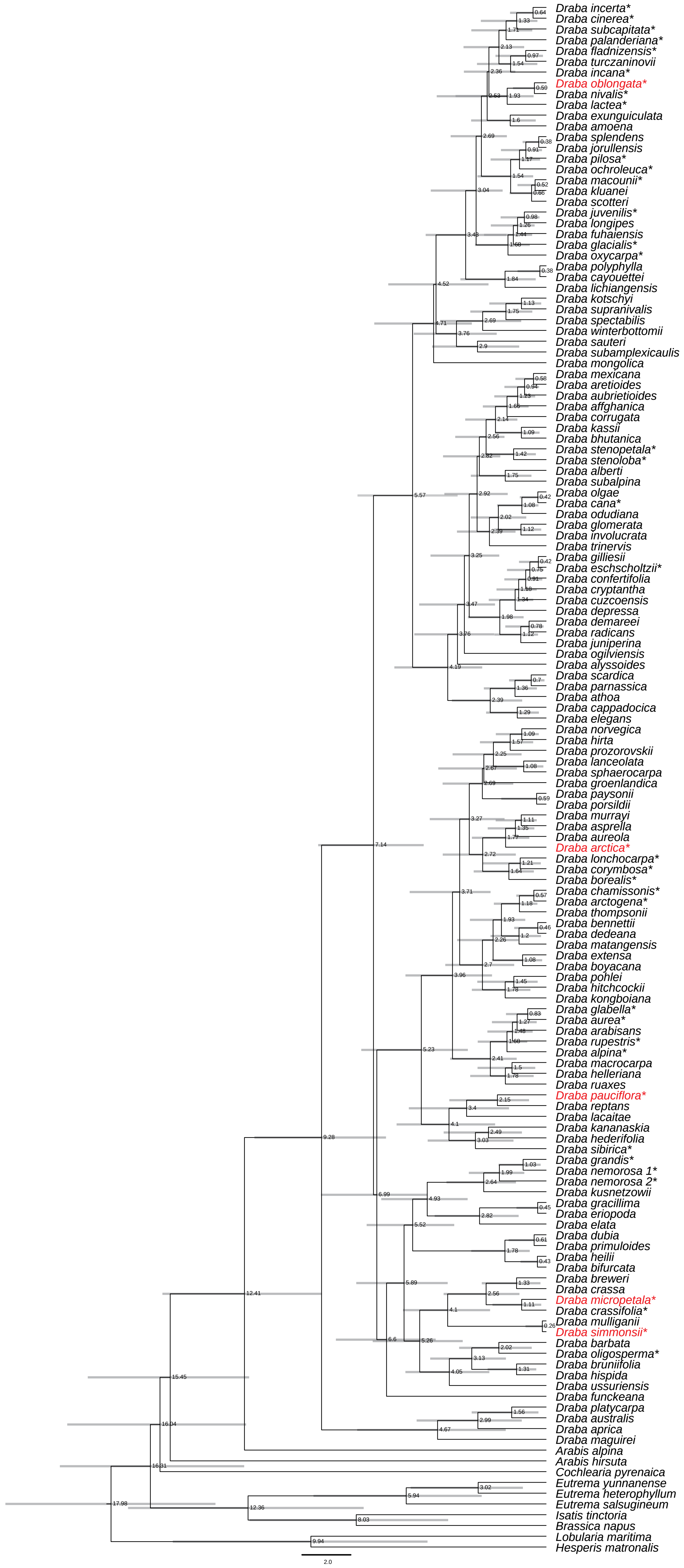

Supplementary Figure 9

Erigeron (Asteraceae)

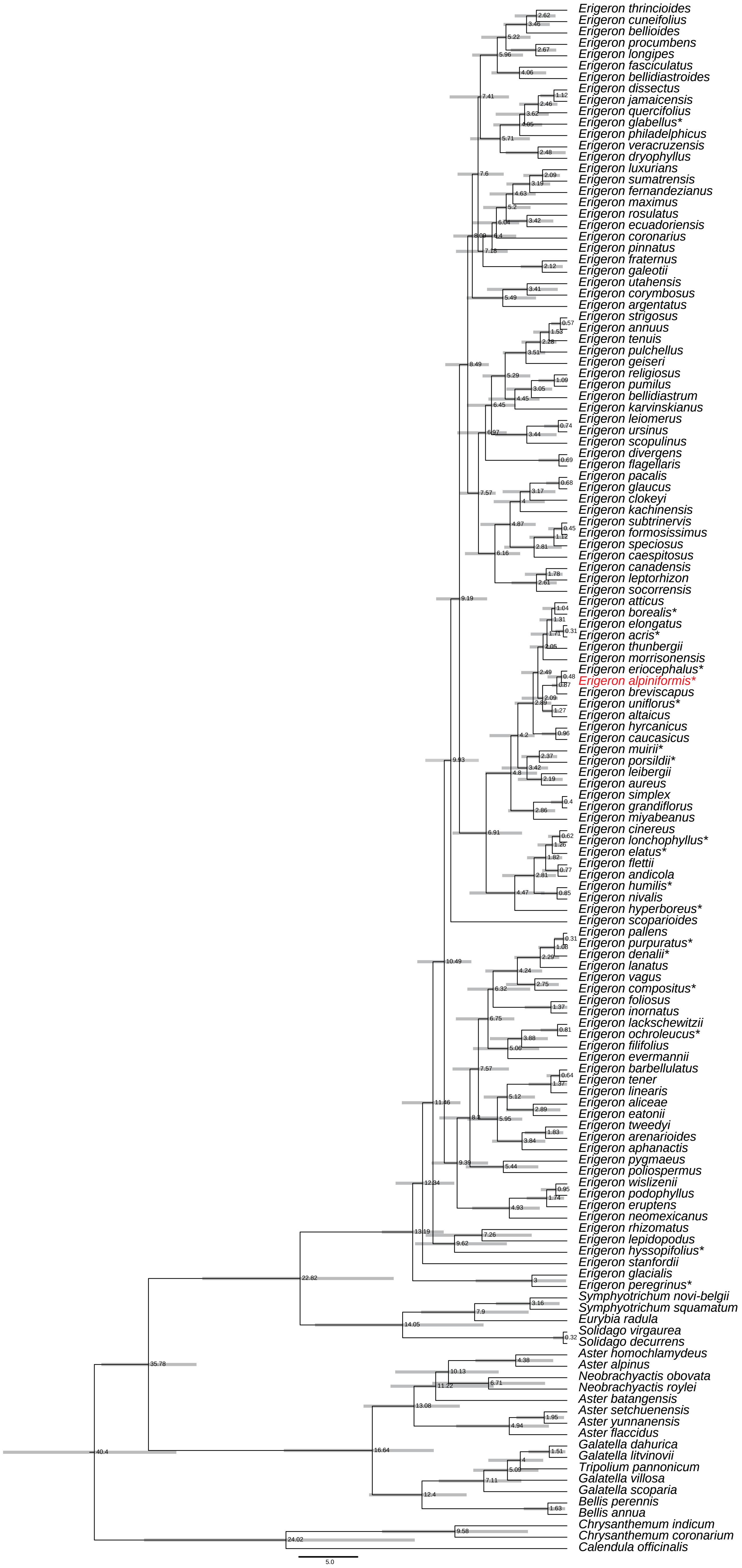

Supplementary Figure 10 *Euphrasia* (Orobanchaceae)

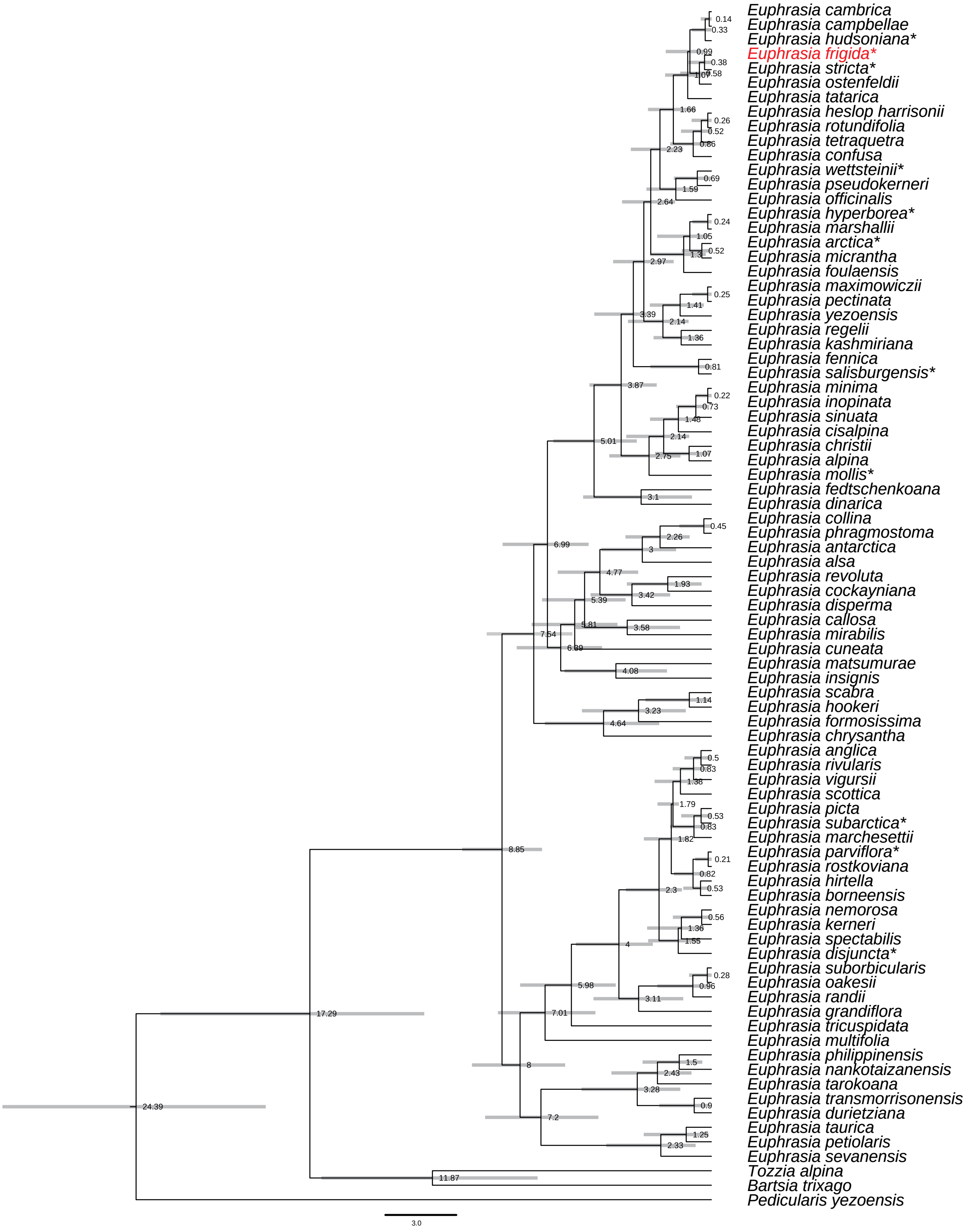

Supplementary Figure 11 *Festuca* (Poaceae)

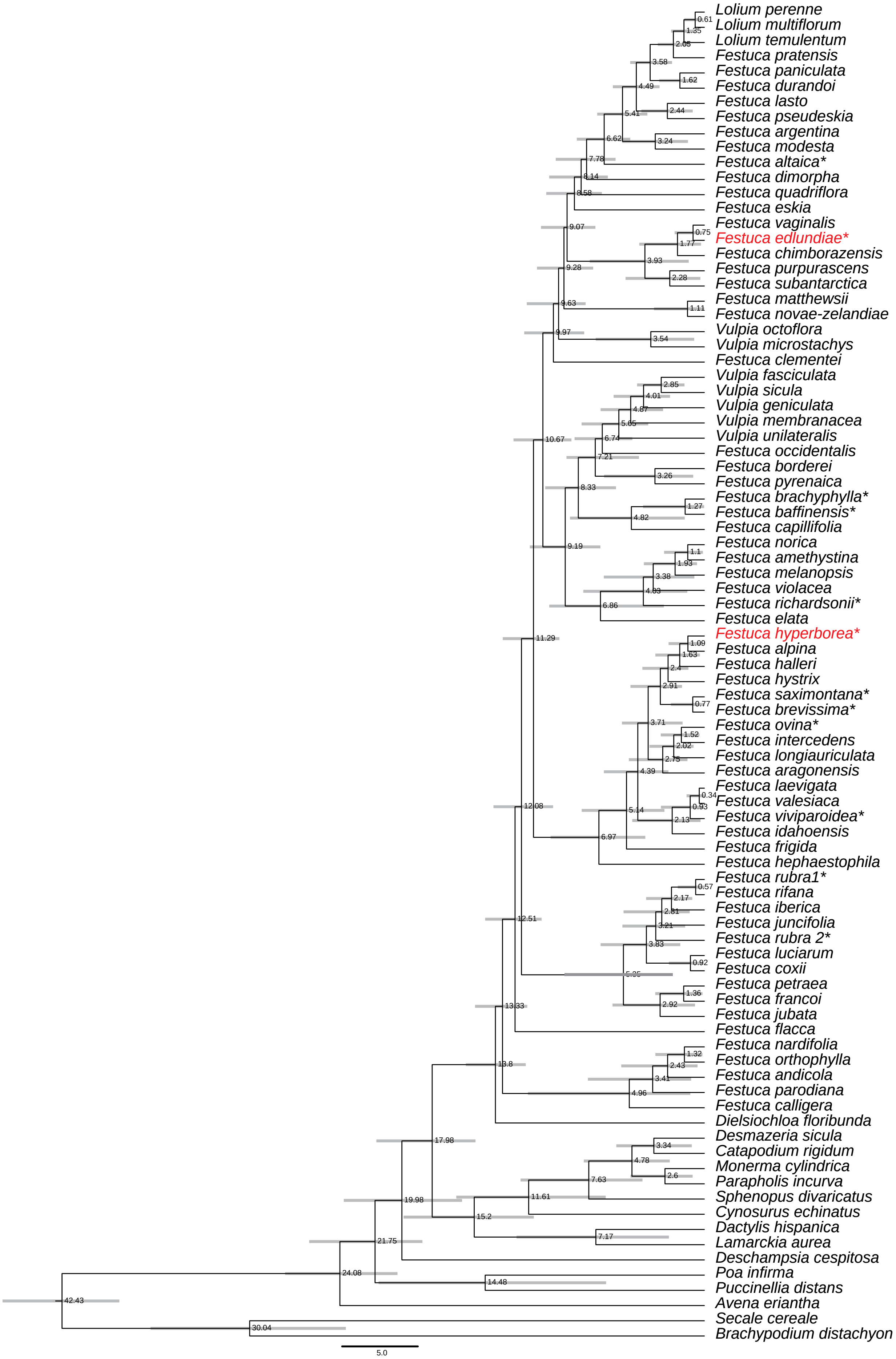

Supplementary Figure 12

Oxytropis (Fabaceae)

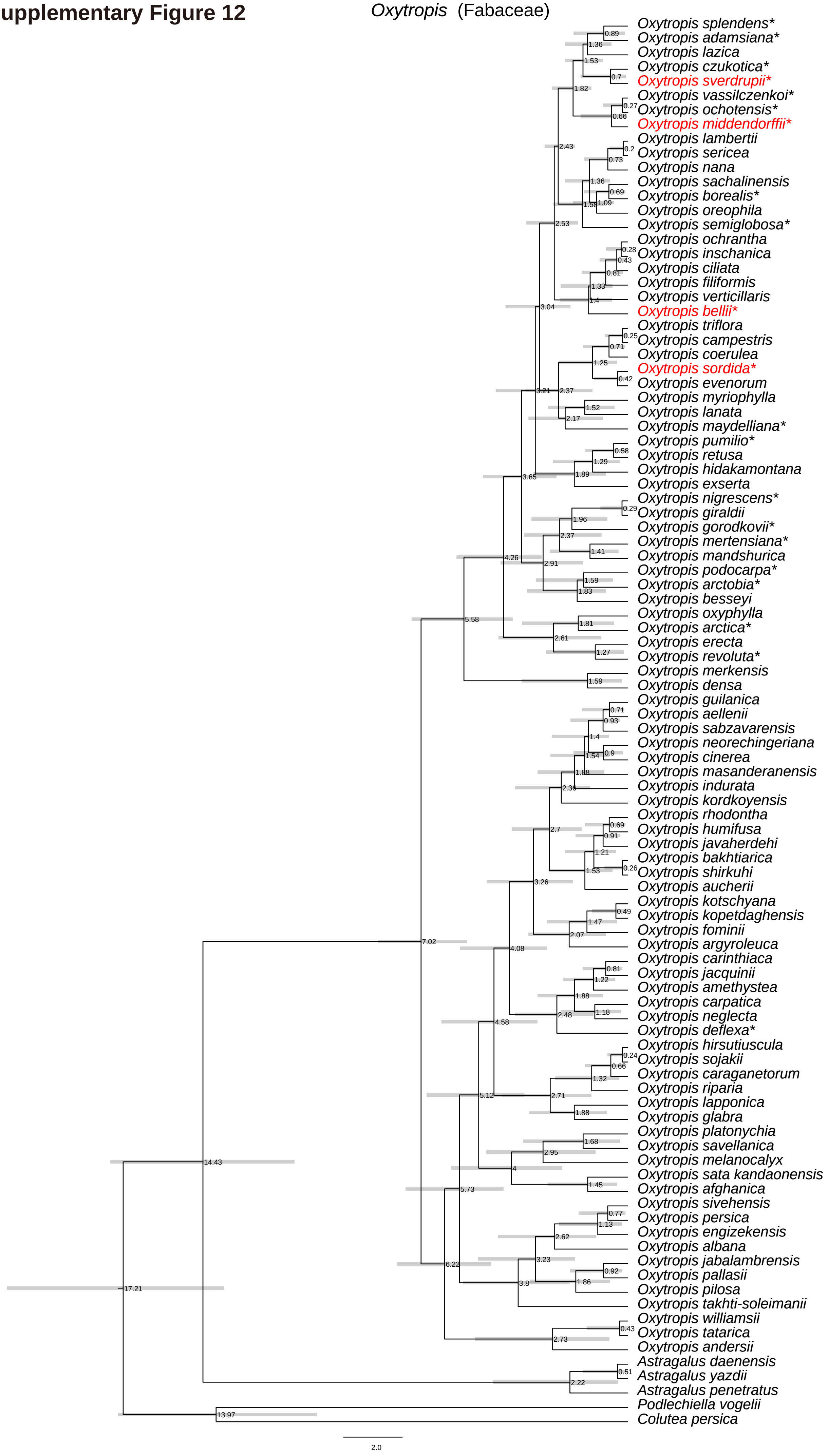

Supplementary Figure 13

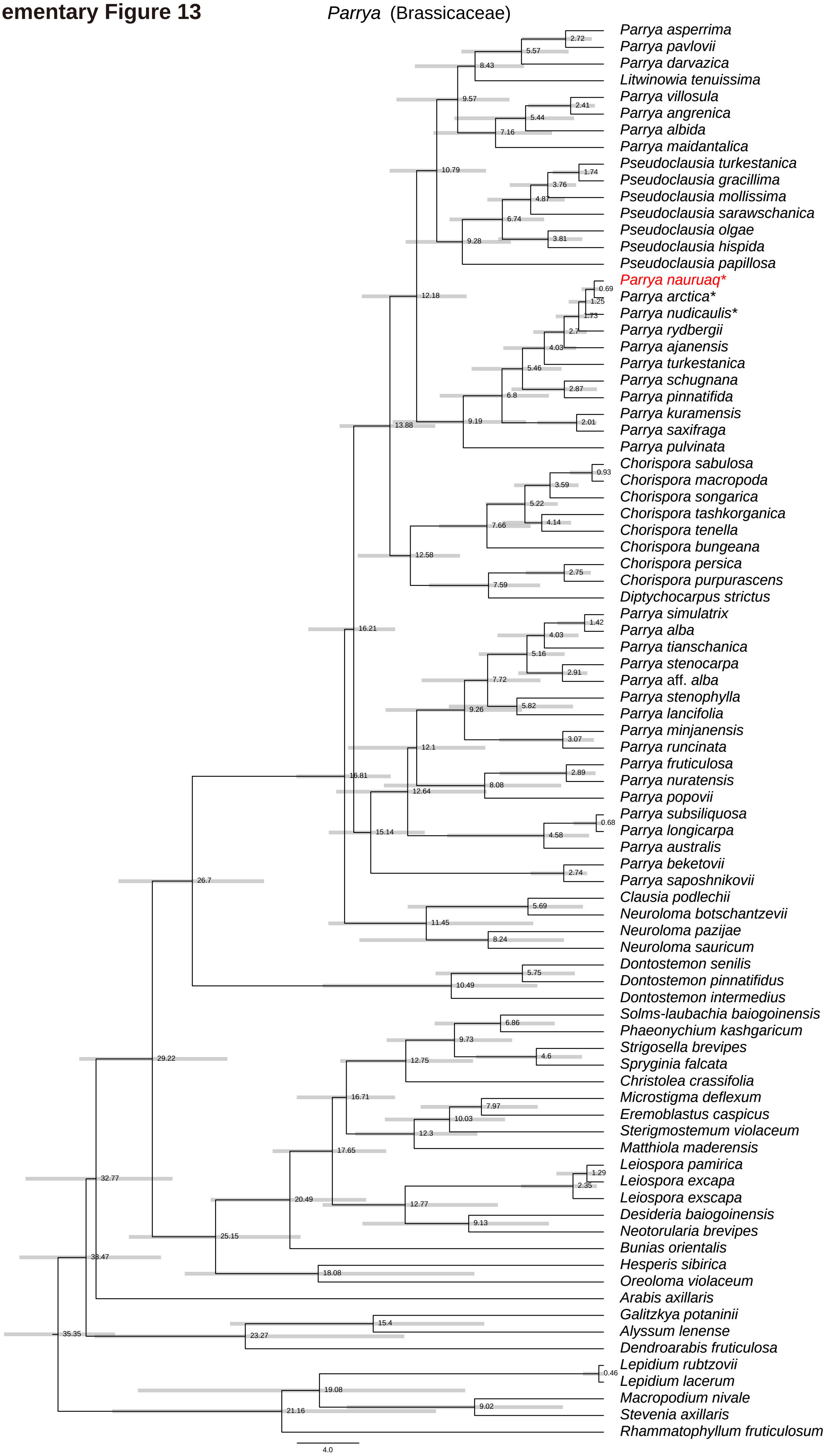

Supplementary Figure 14 *Pedicularis* (Orobanchaceae)

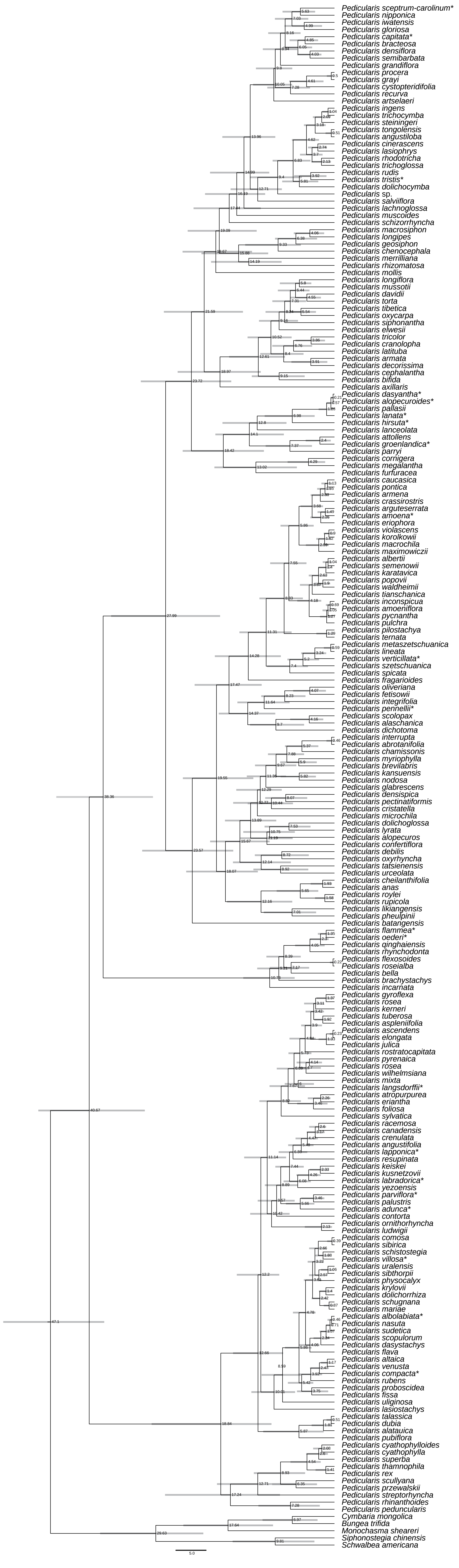

Supplementary Figure 15 *Plantago* (Plantaginaceae)

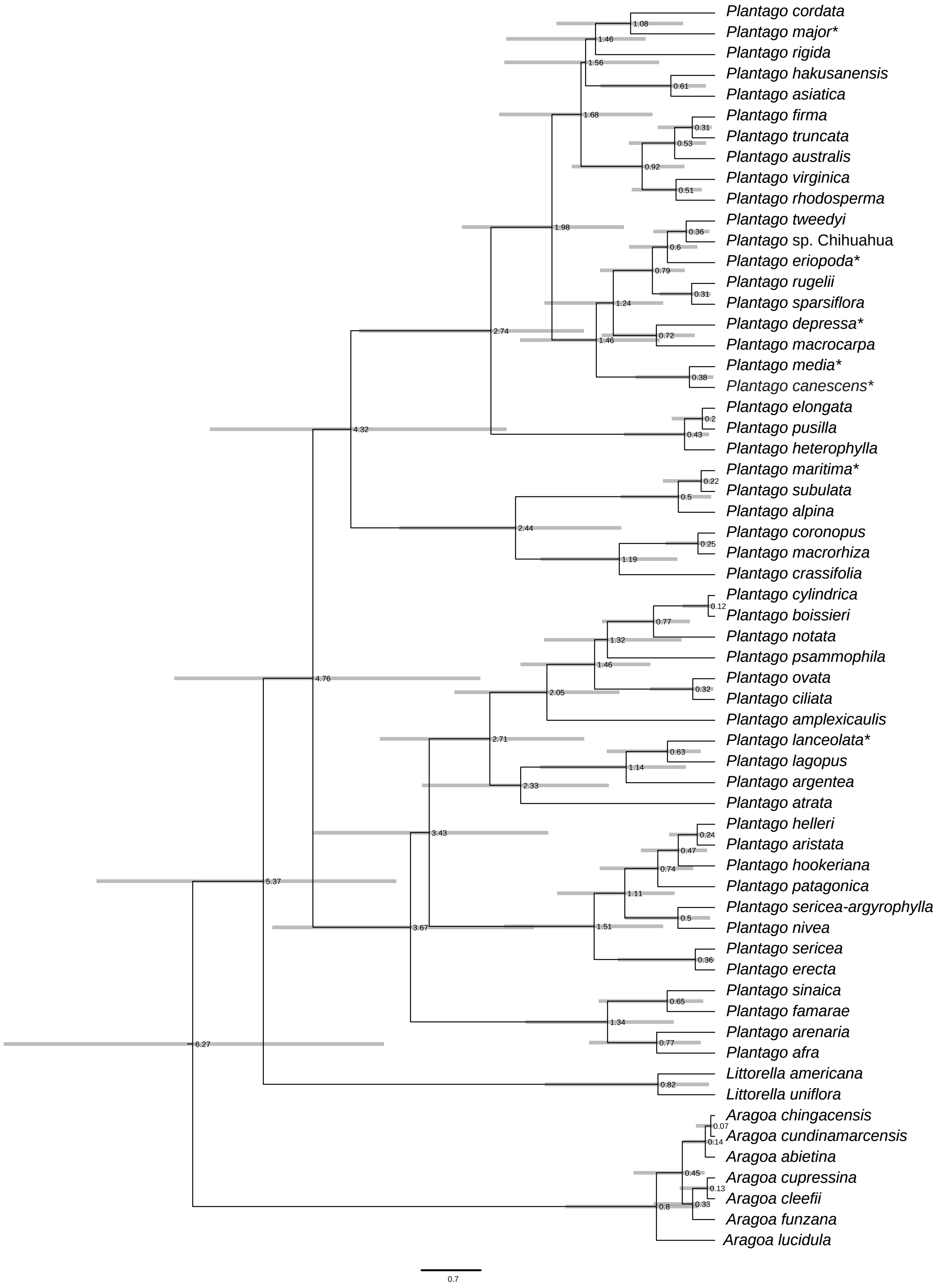

Supplementary Figure 16 *Poa* (Poaceae)

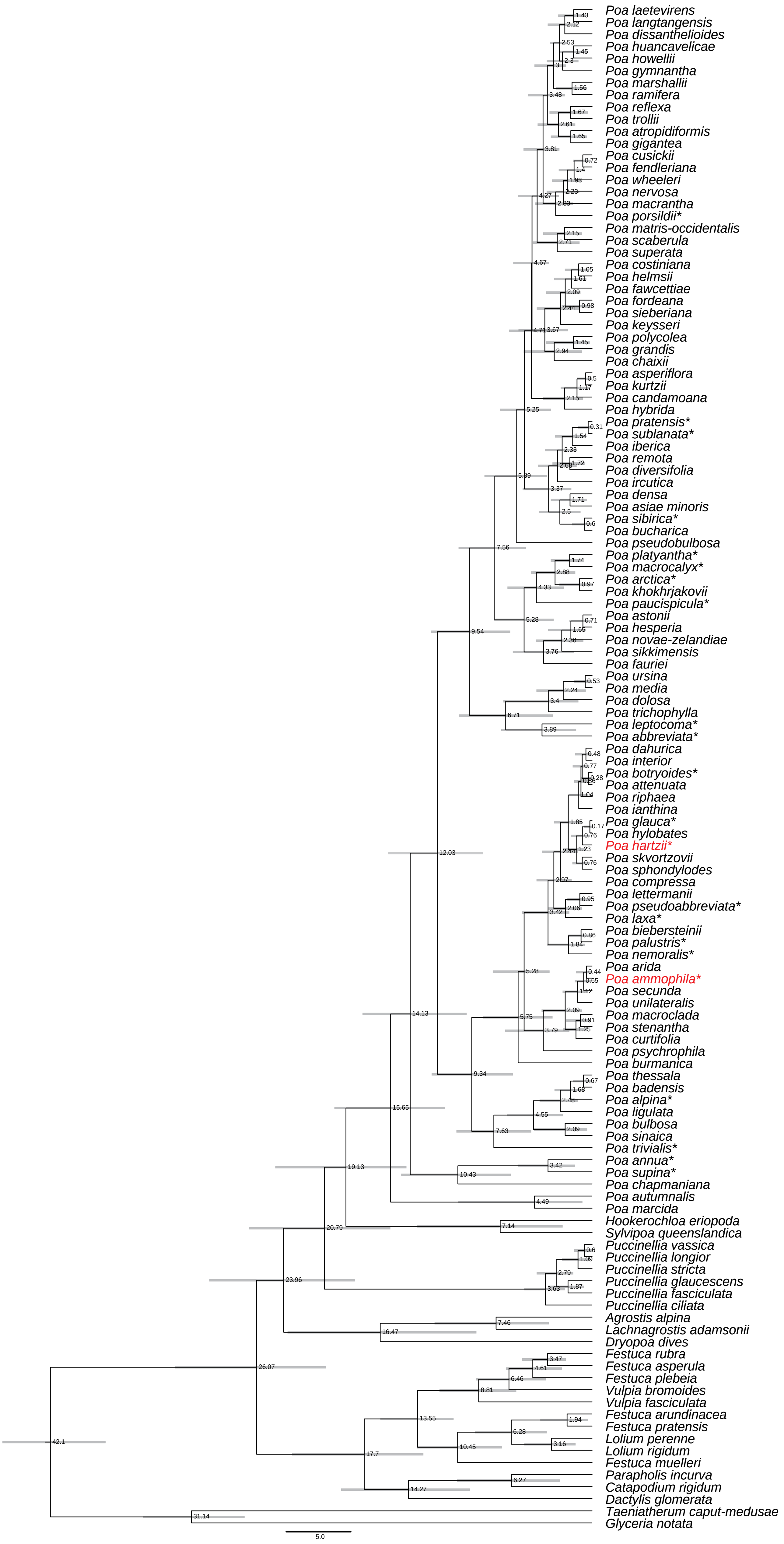

Supplementary Figure 17

Potentilla (Rosaceae)

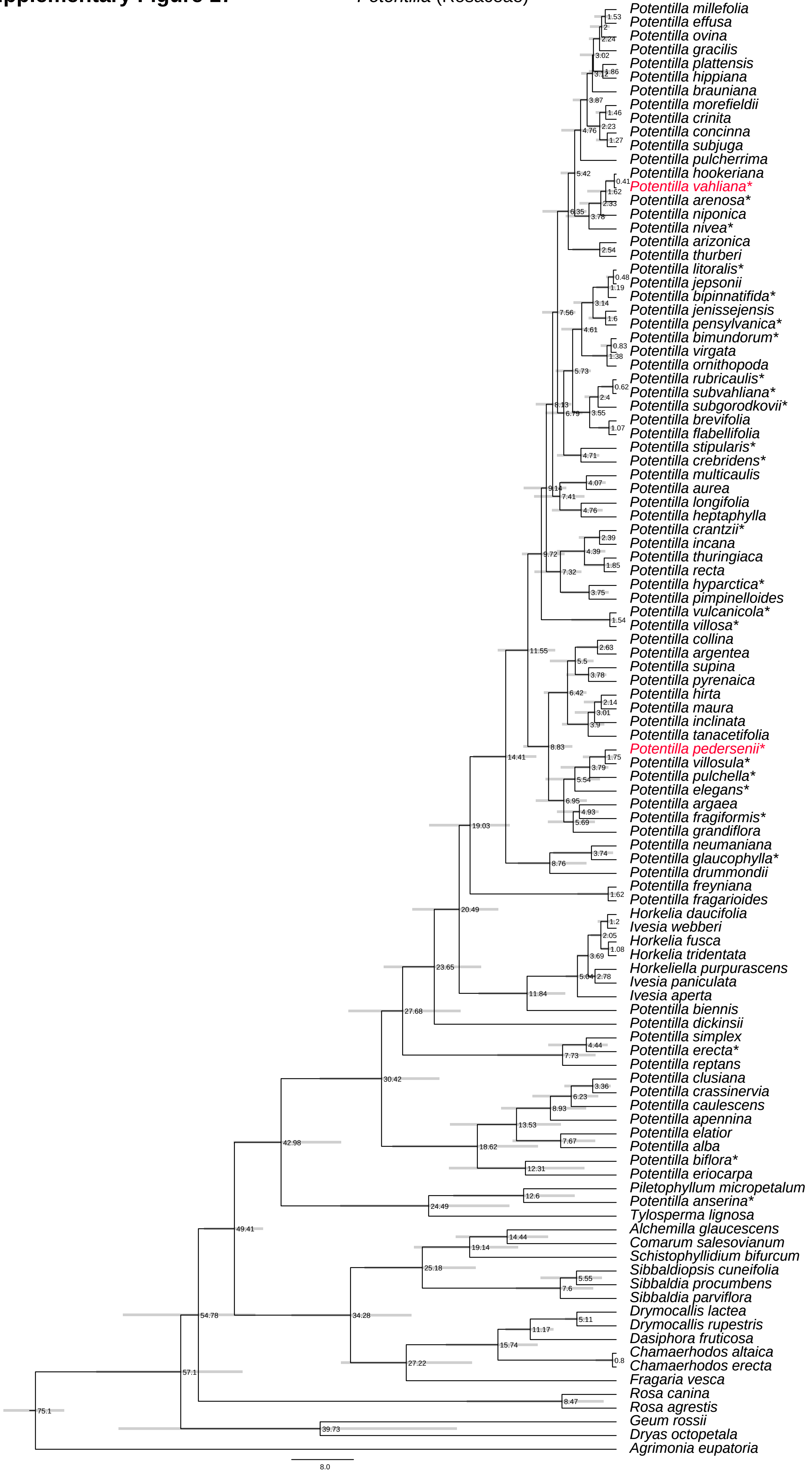

Supplementary Figure 18 *Primula* (Primulaceae)

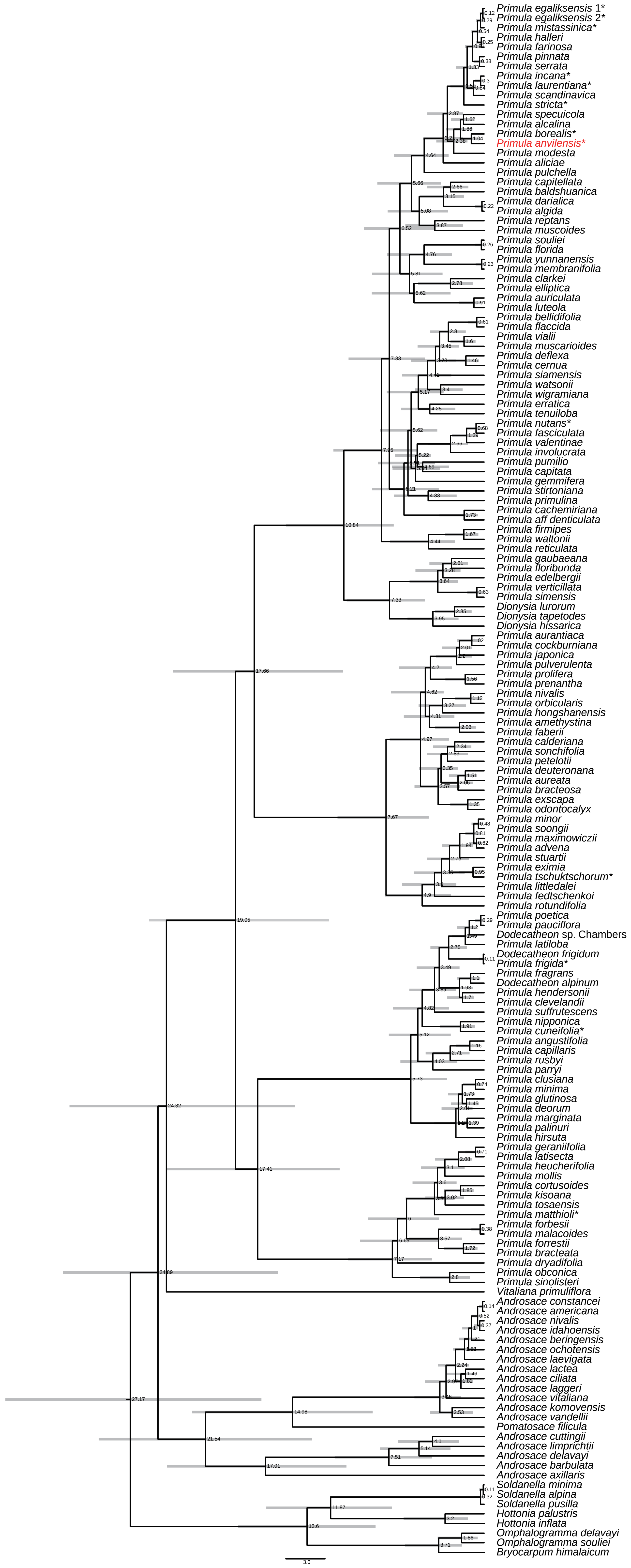

Supplementary Figure 19 *Puccinellia* (Poaceae)

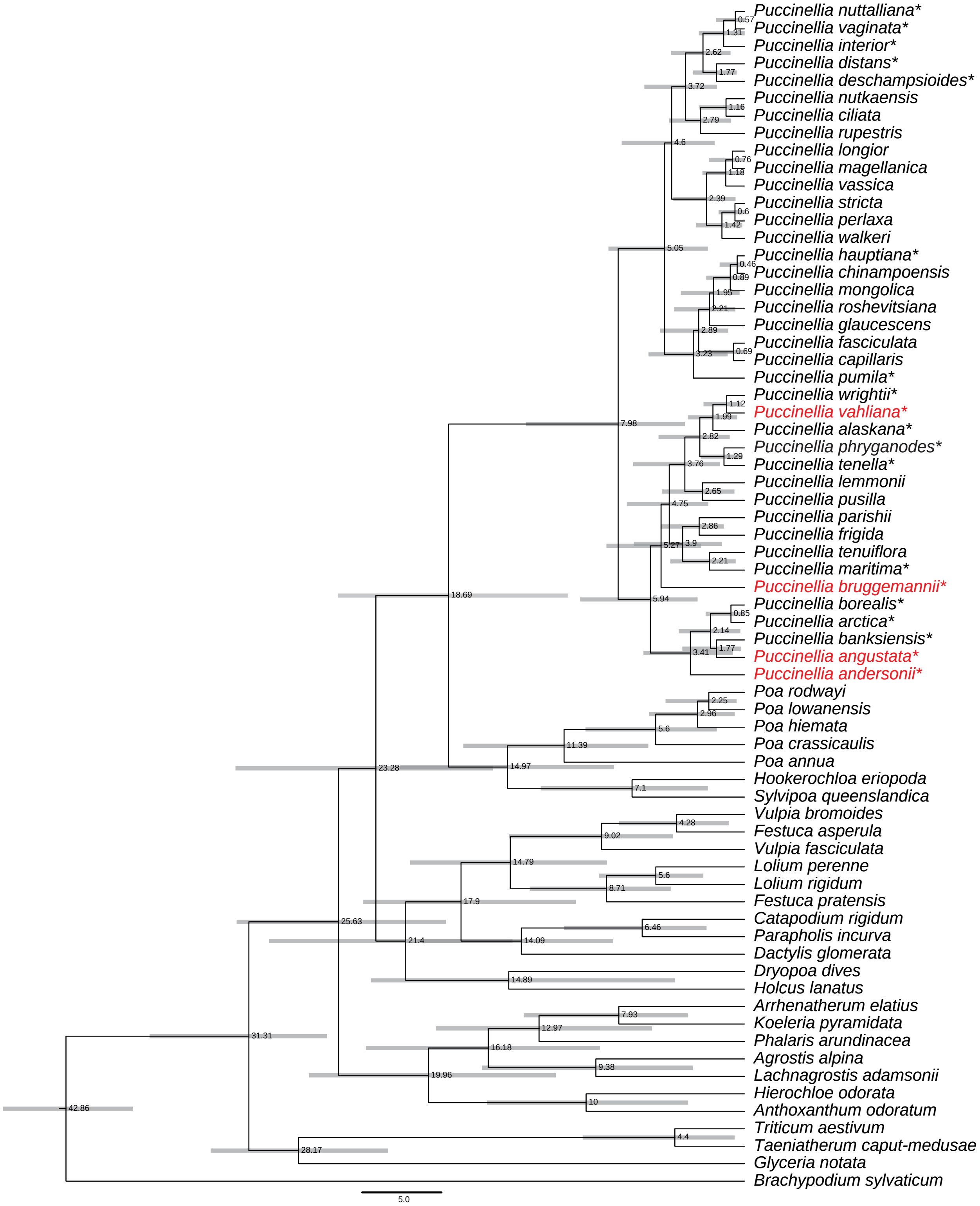

Supplementary Figure 20 *Ranunculus* (Ranunculaceae)

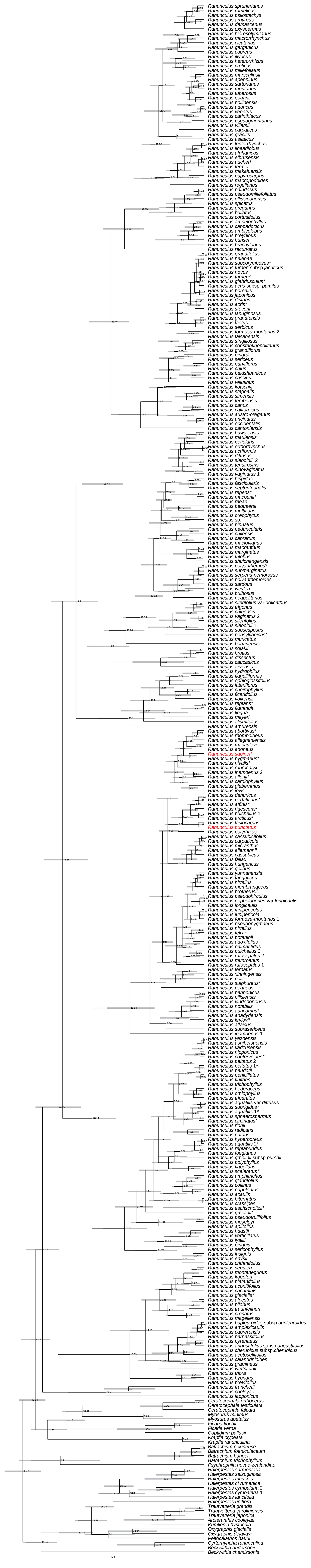

Supplementary Figure 21 *Rumex* (Polygonaceae)

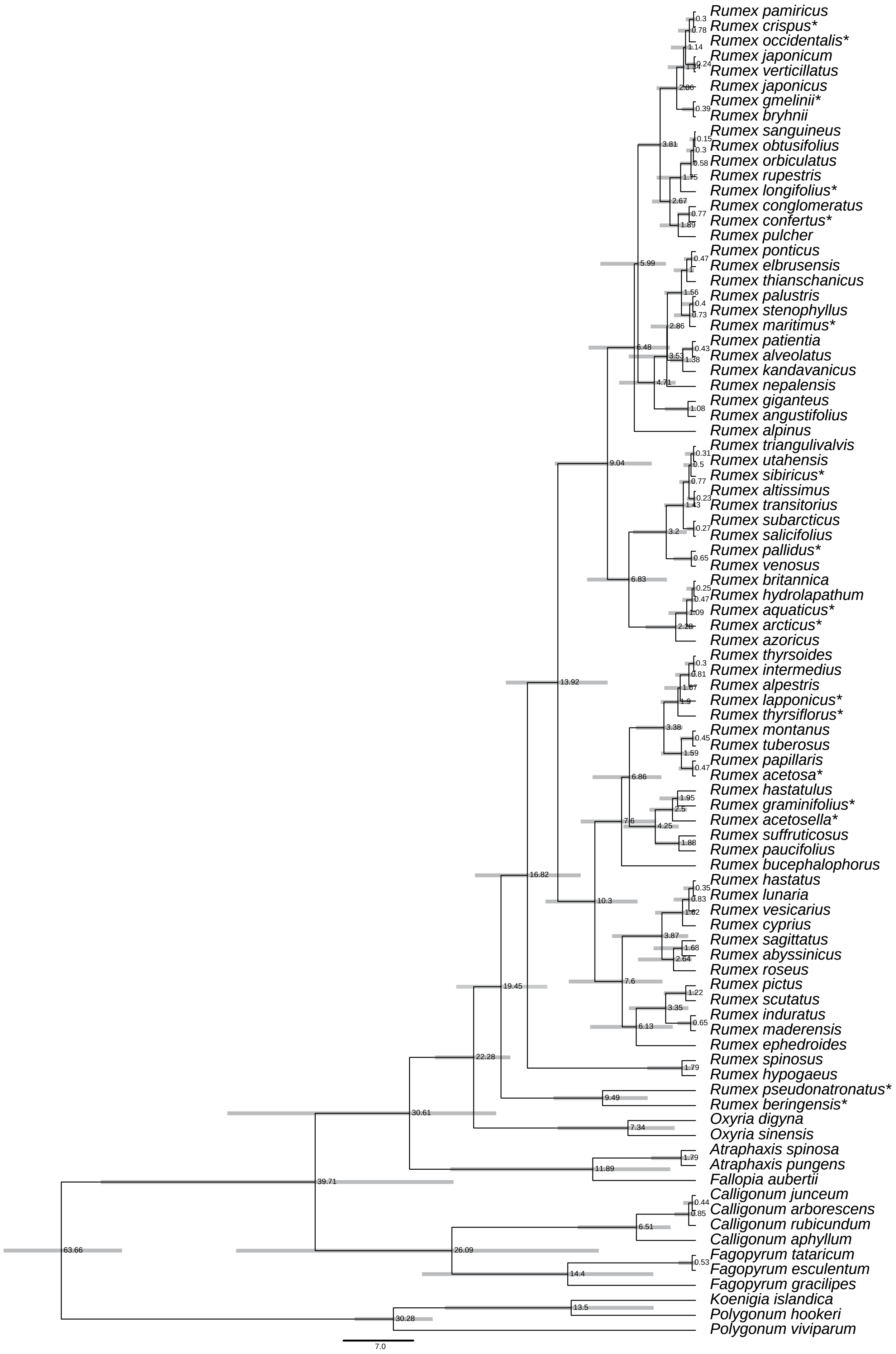

Supplementary Figure 22 *Saxifraga* (Saxifragaceae)

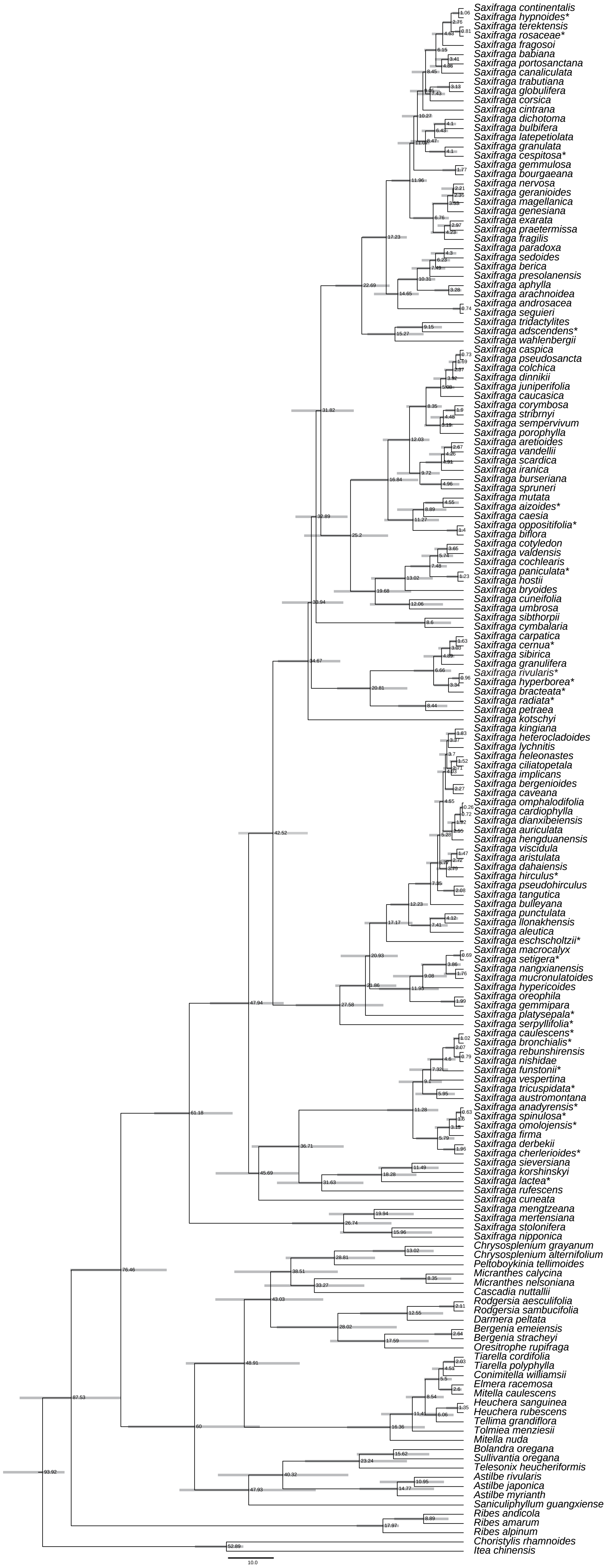

Supplementary Figure 23 *Silene* (Caryophyllaceae)

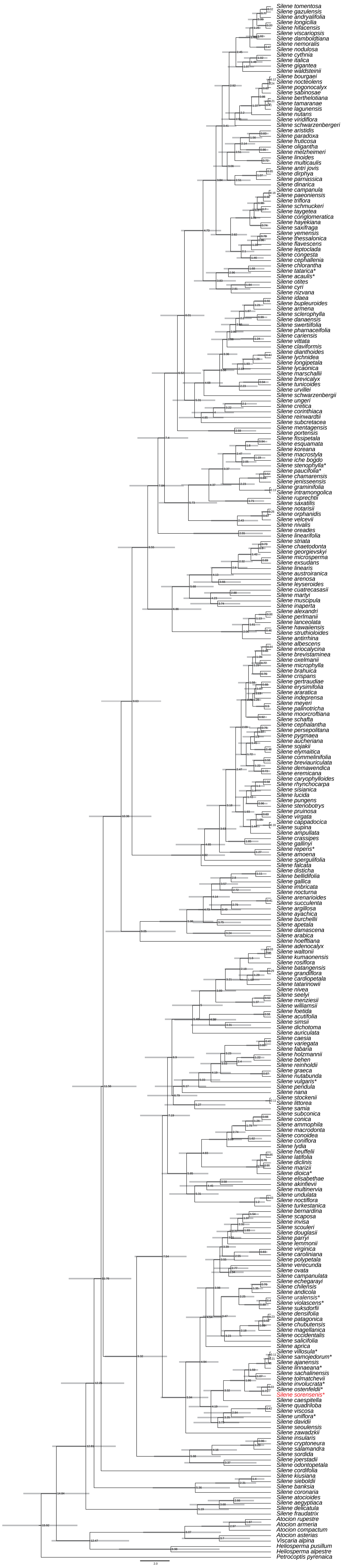

Supplementary Figure 24

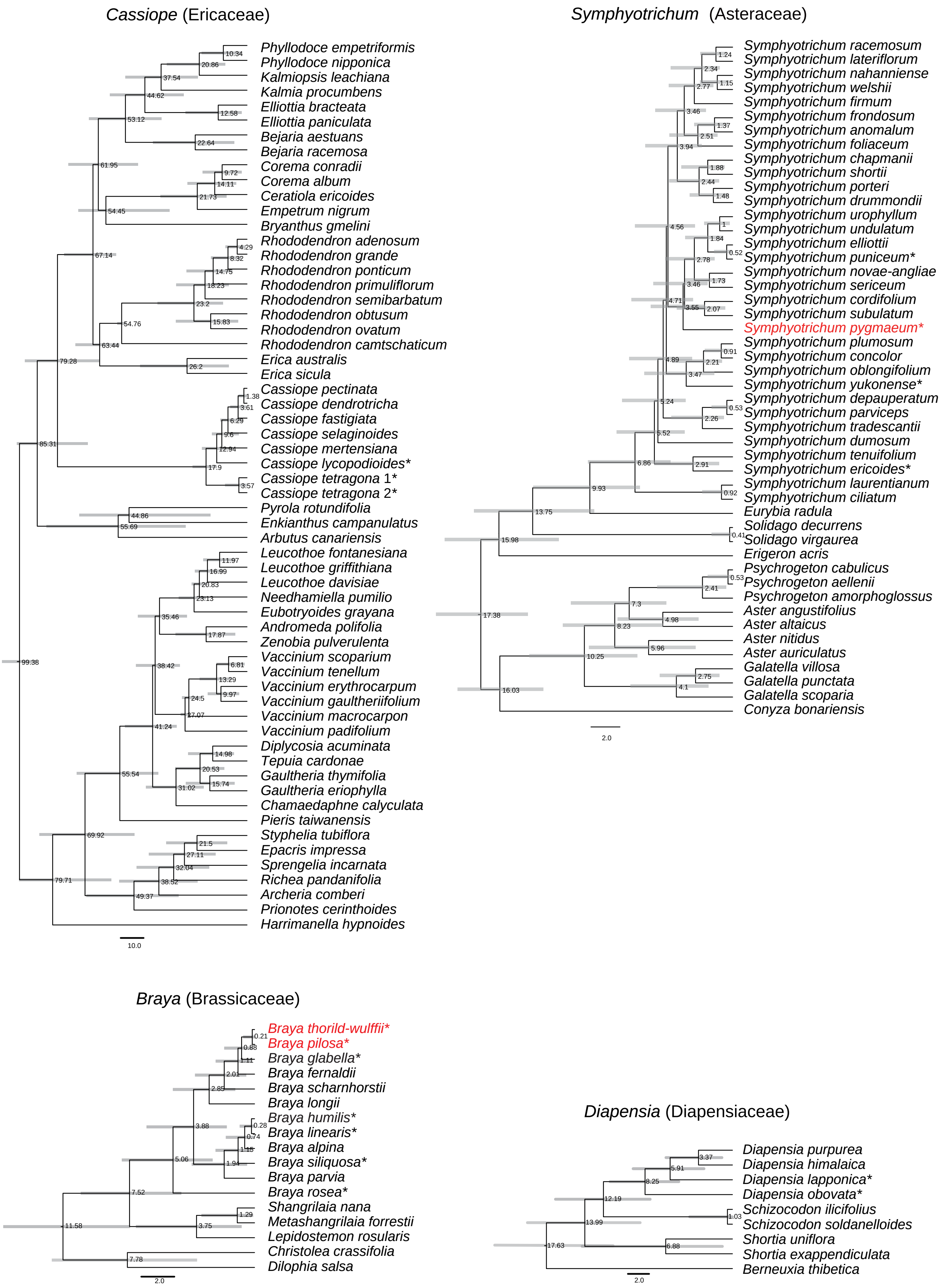

Supplementary Figure 25

Douglasia-Androsace clade (Primulaceae)

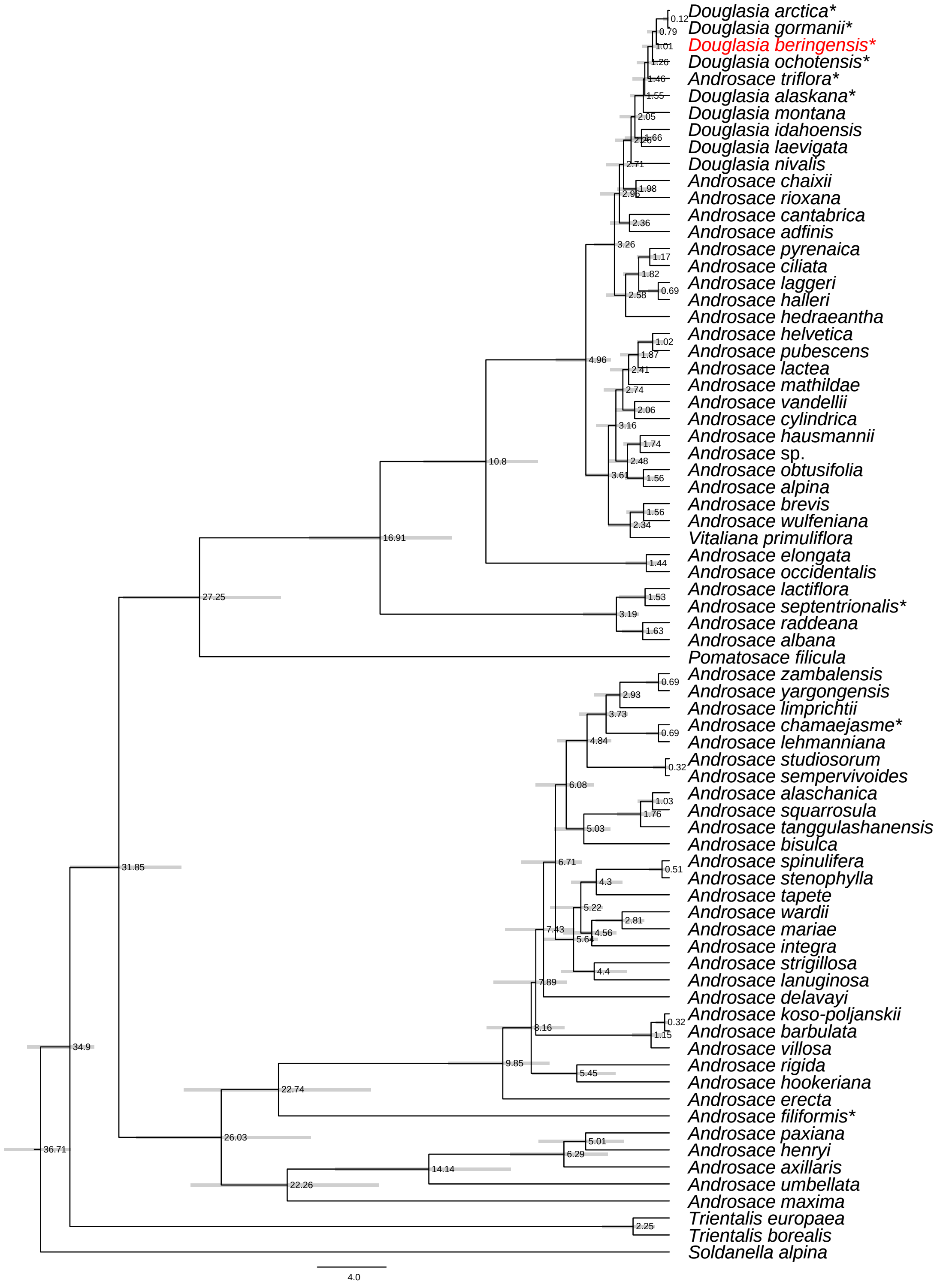

Smelowskia (Brassicaceae)

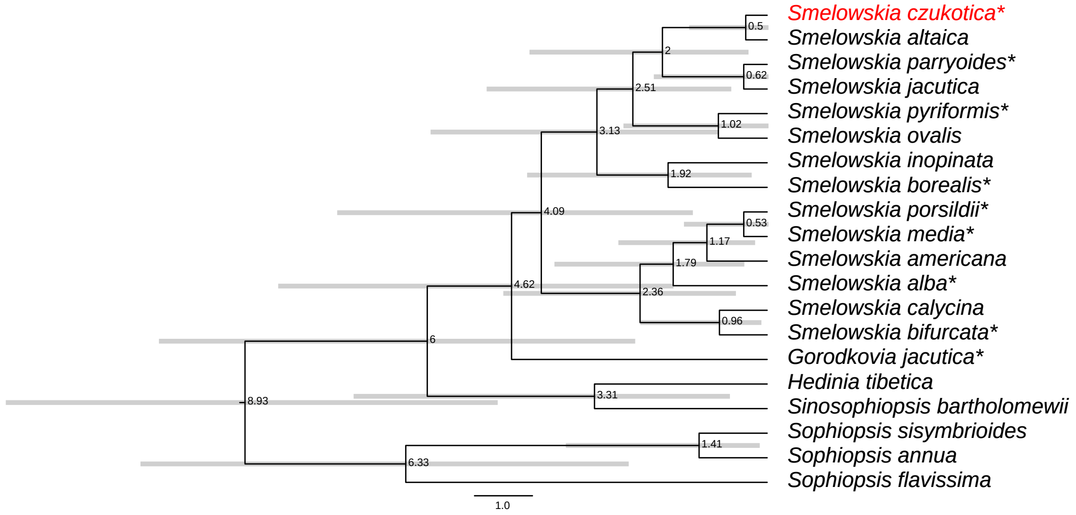

Supplementary Figure 26

Pleuropogon (Poaceae)

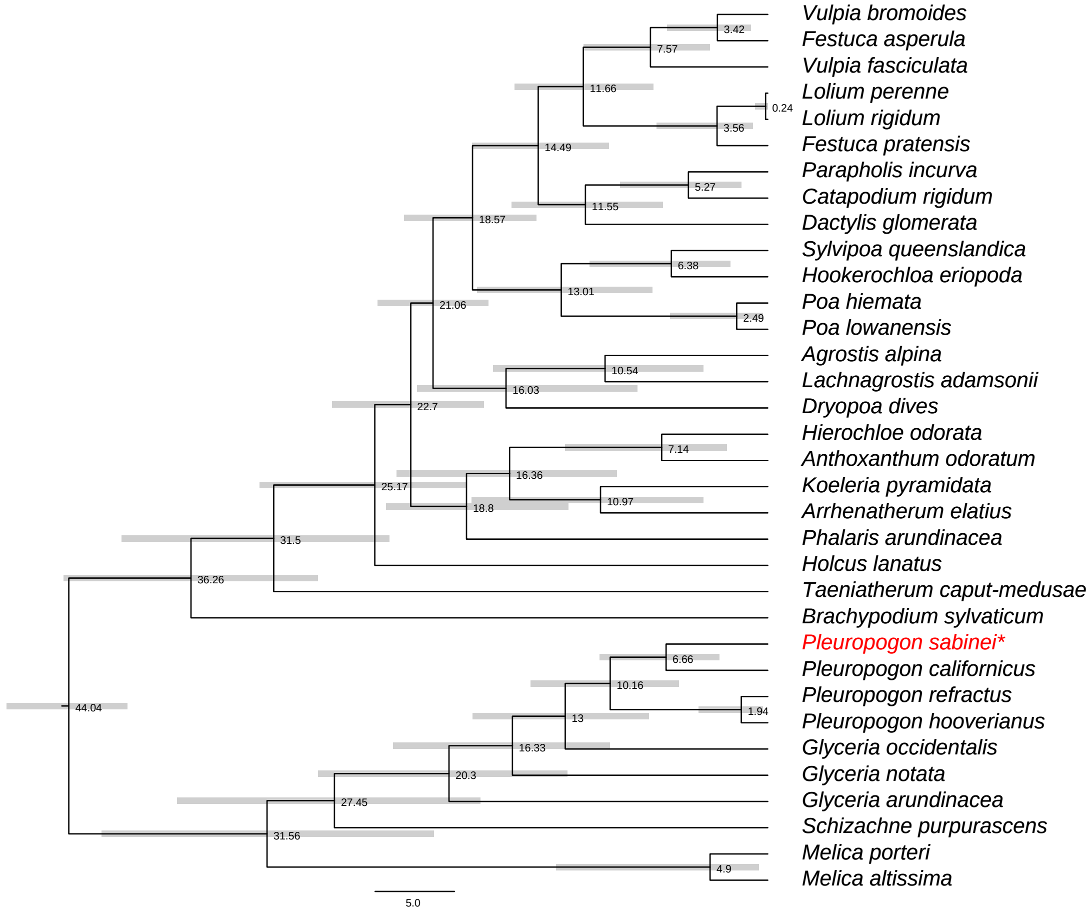

Packera (Asteraceae)

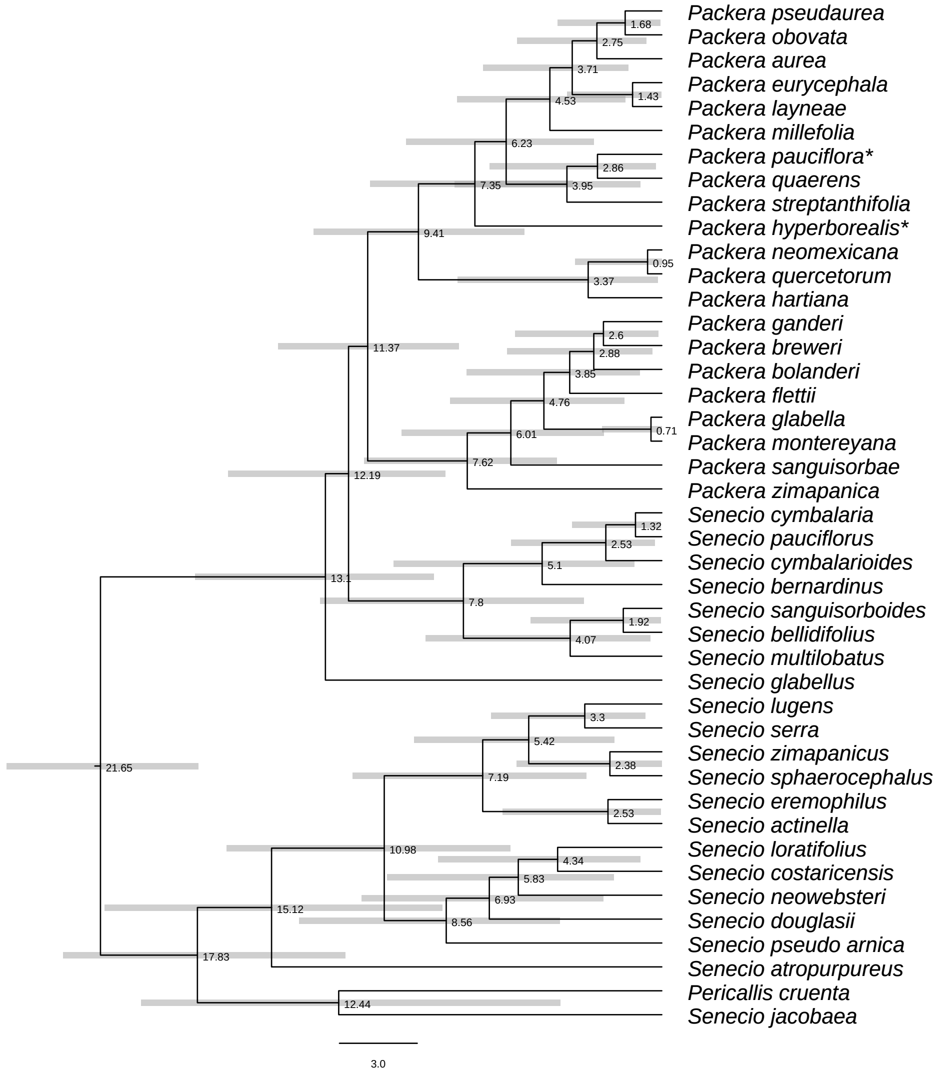

Supplementary Figure 27

Arctic *Papaver* clade (Papaveraceae)

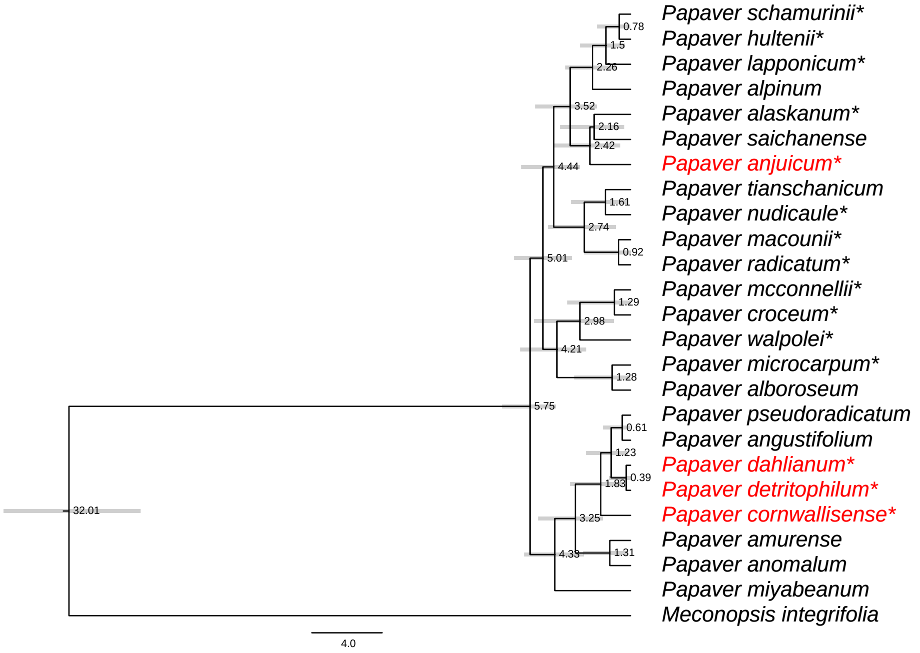

Chrysosplenium (Saxifragaceae)

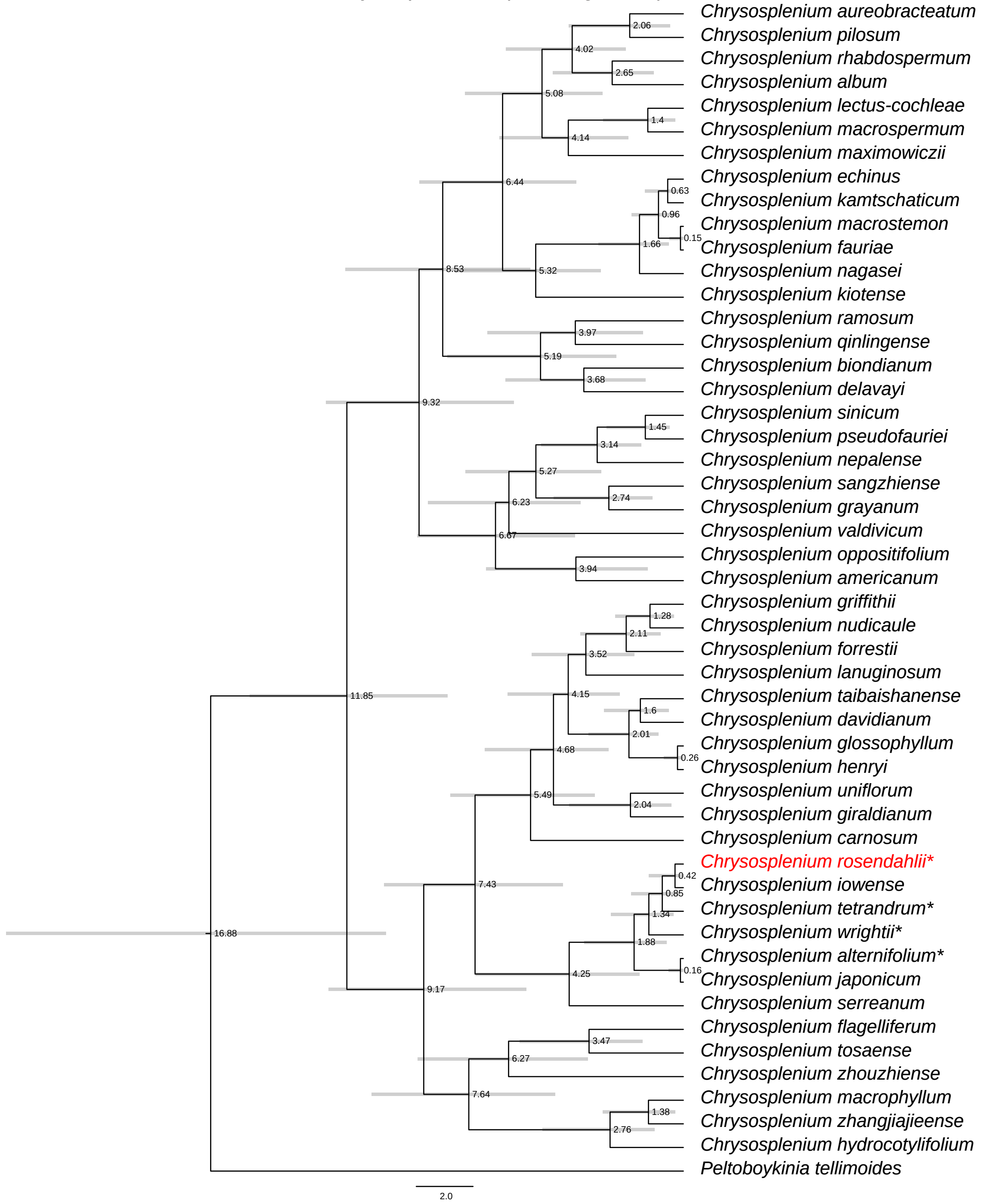

Supplementary Figure 28

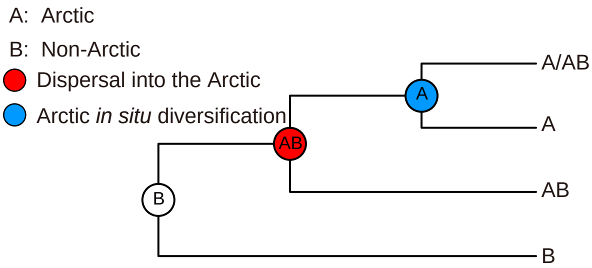

Supplementary Figure 29

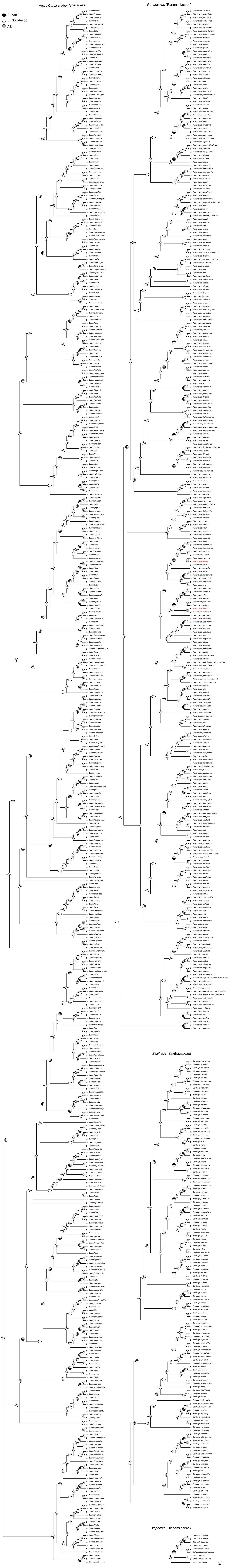

Supplementary Figure 30

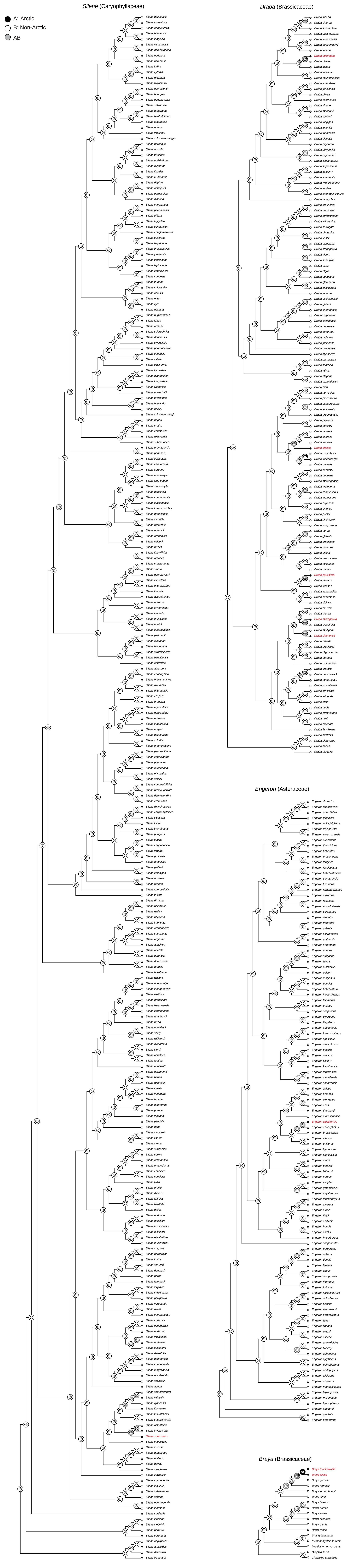

Supplementary Figure 31

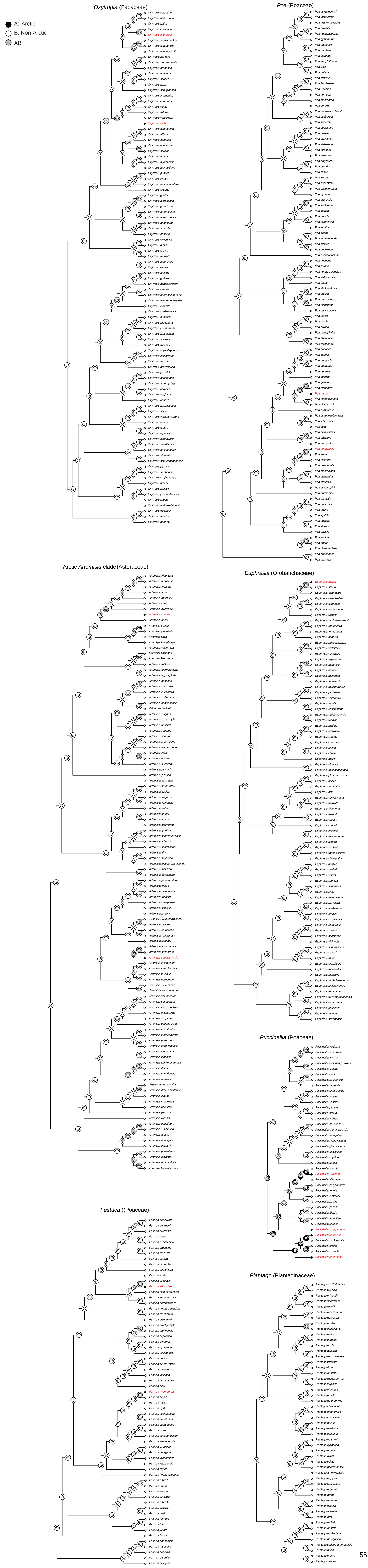

Supplementary Figure 32

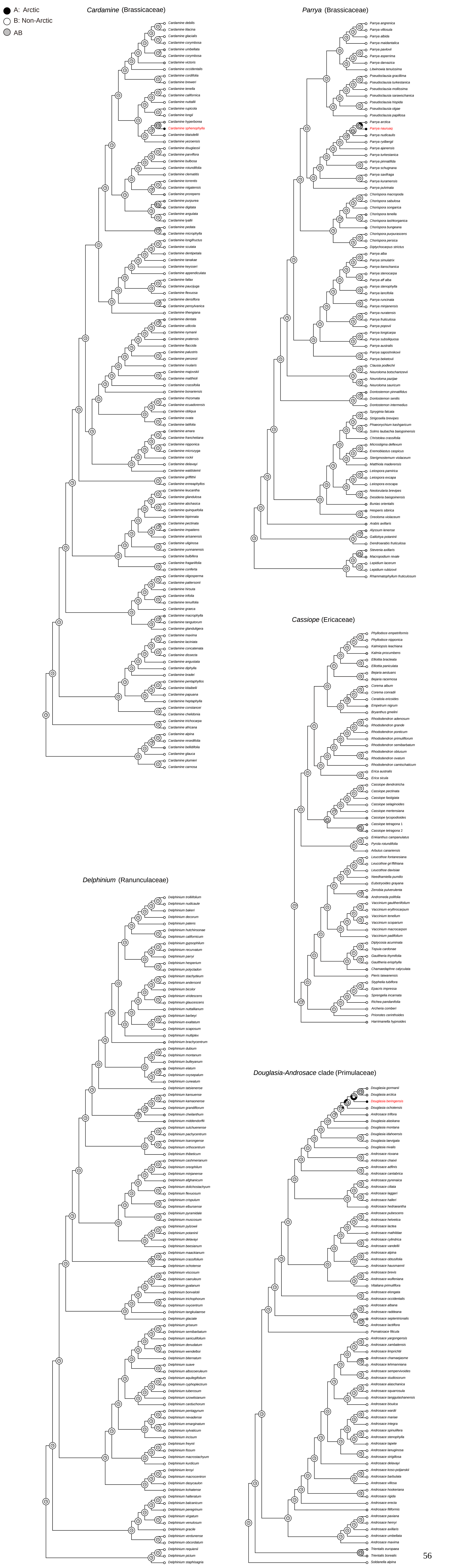

Supplementary Figure 33

A: Arctic
B: Non-Arctic
AB

Cerastium (Caryophyllaceae)

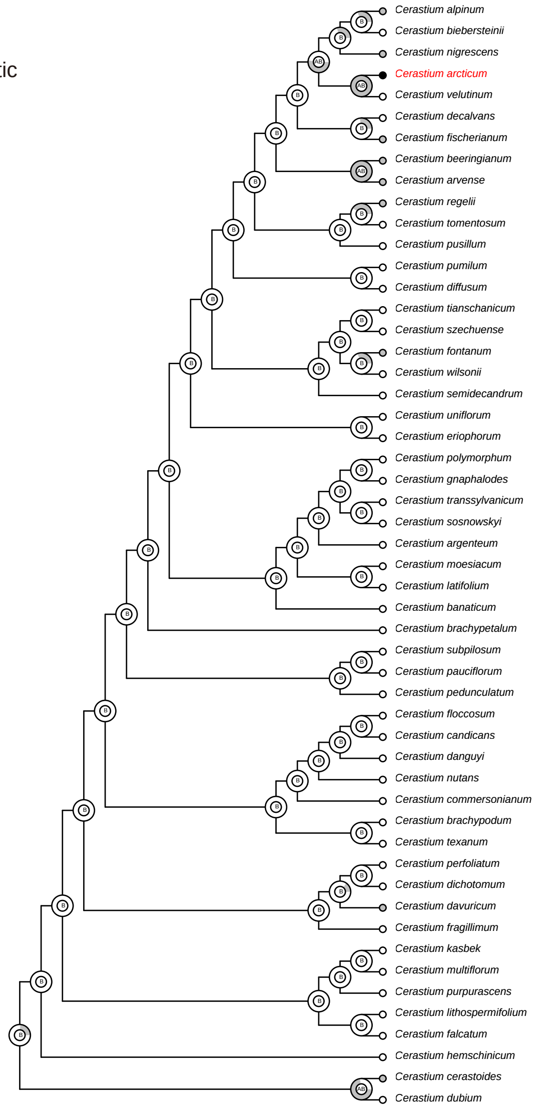

Chrysosplenium (Saxifragaceae)

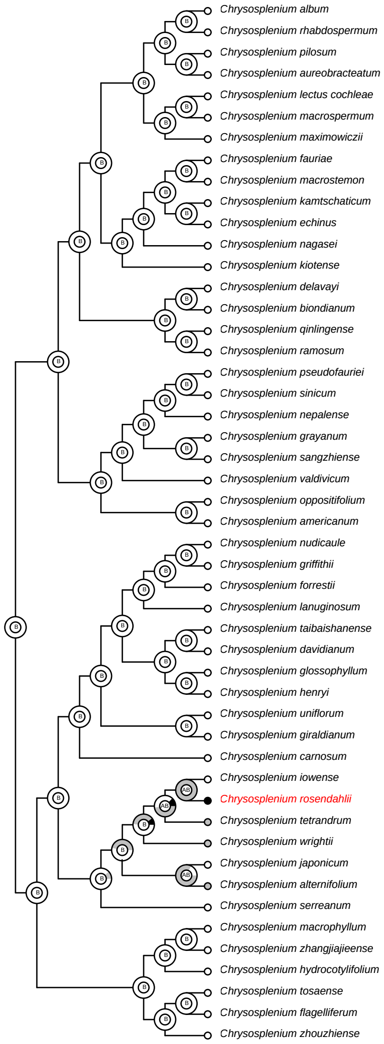

Symphyotrichum (Asteraceae)

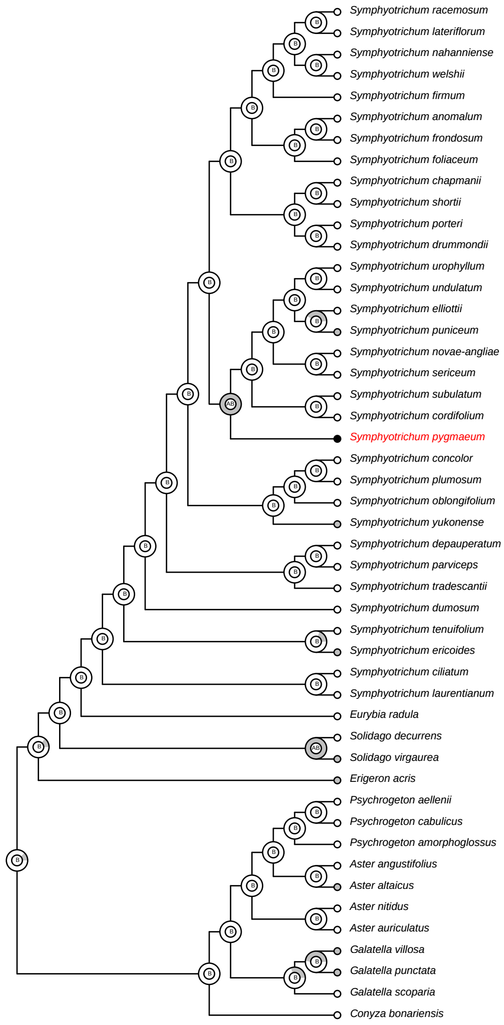

Packera (Asteraceae)

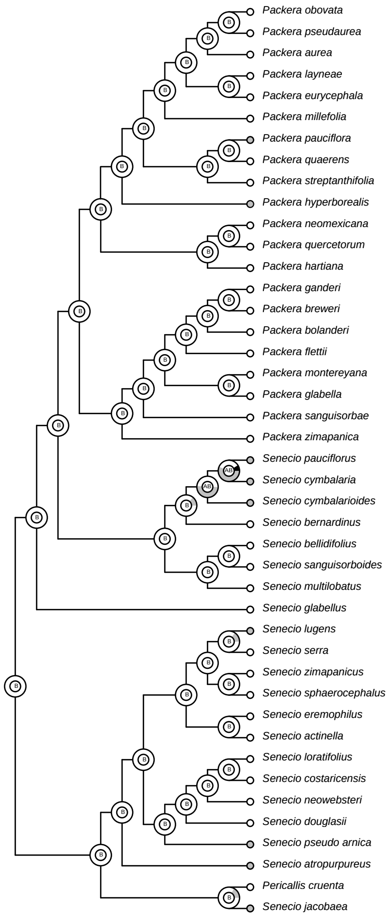

Supplementary Figure 34

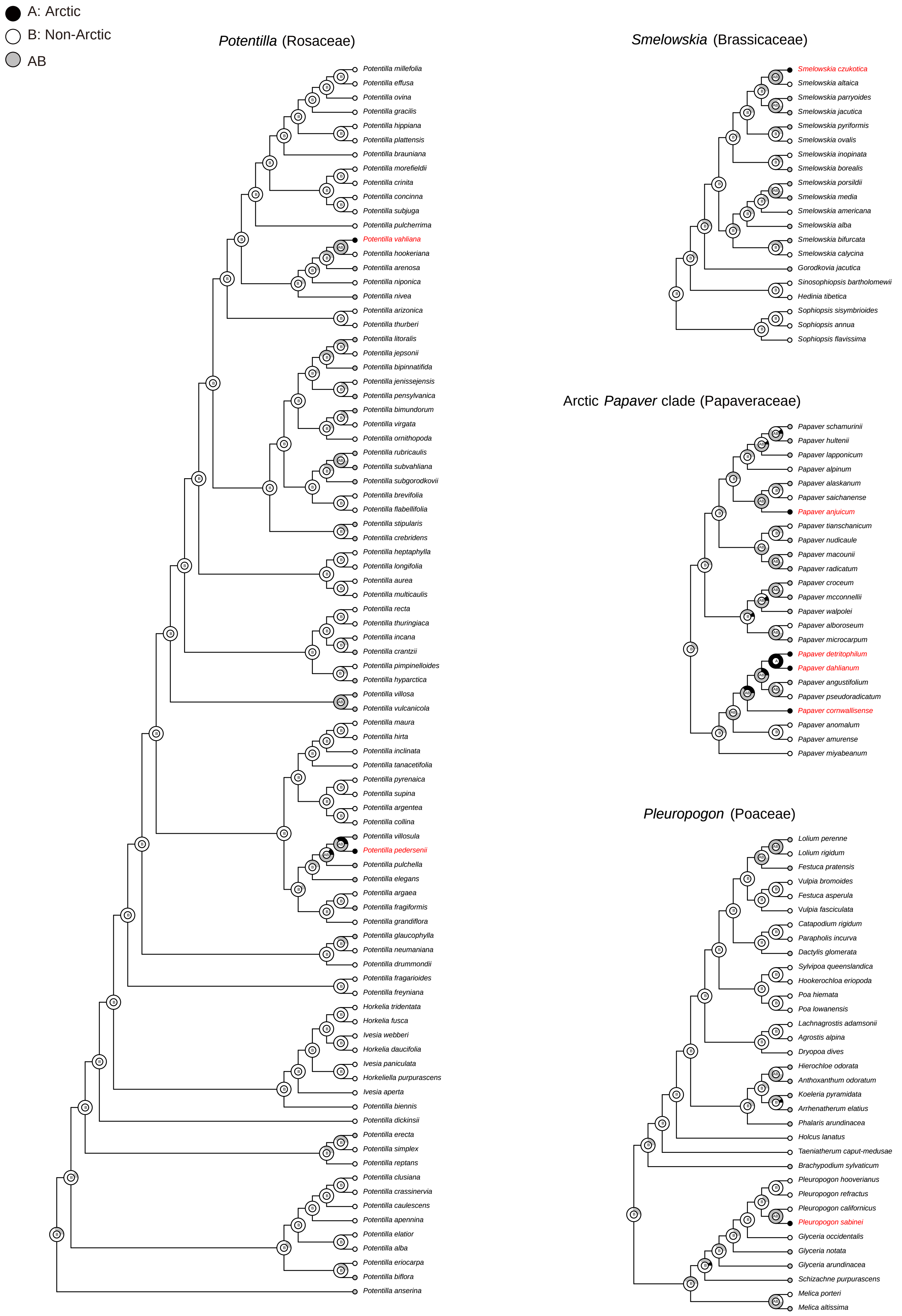

Supplementary Figure 35

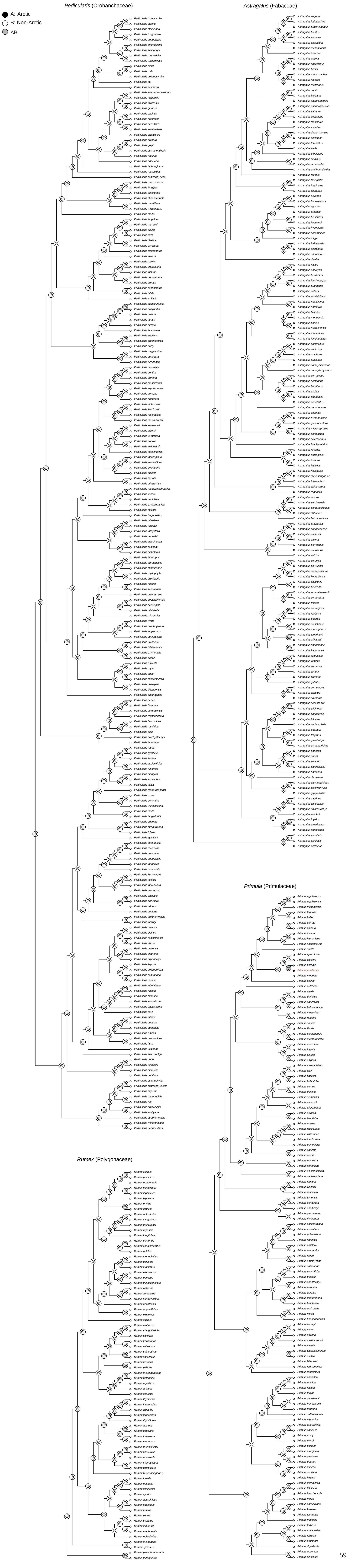

Supplementary Figure 36

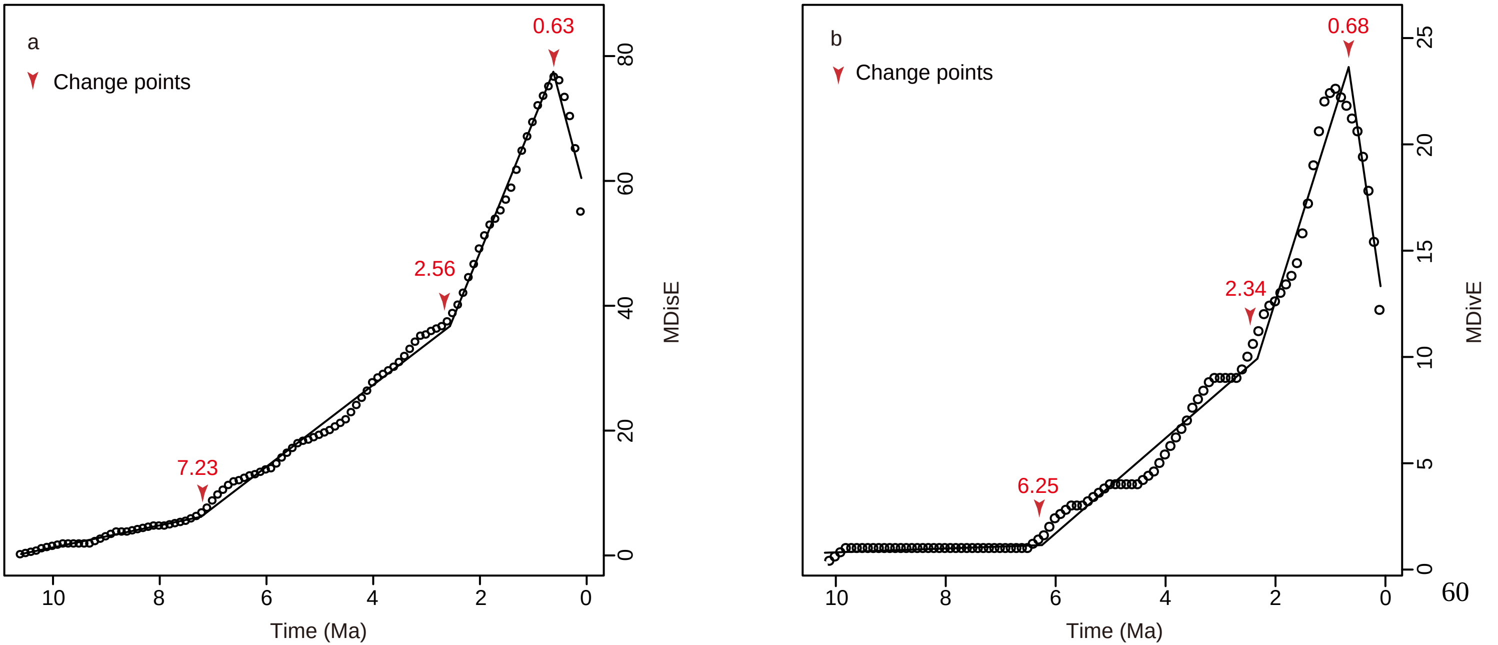

Supplementary Figure 37

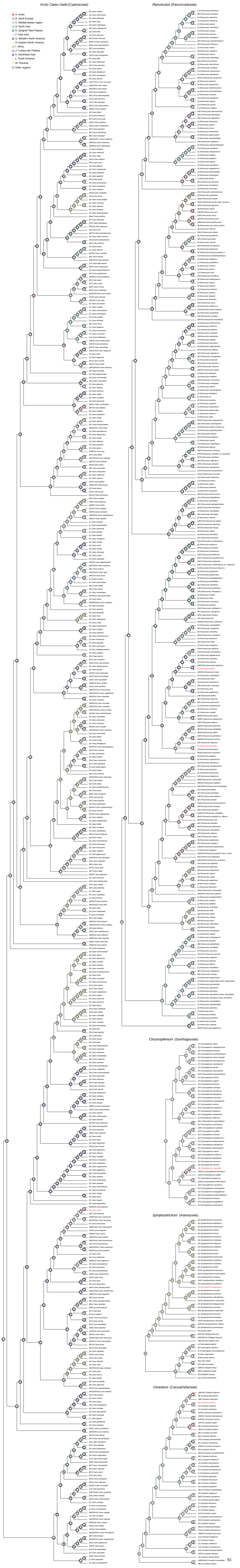

Supplementary Figure 38

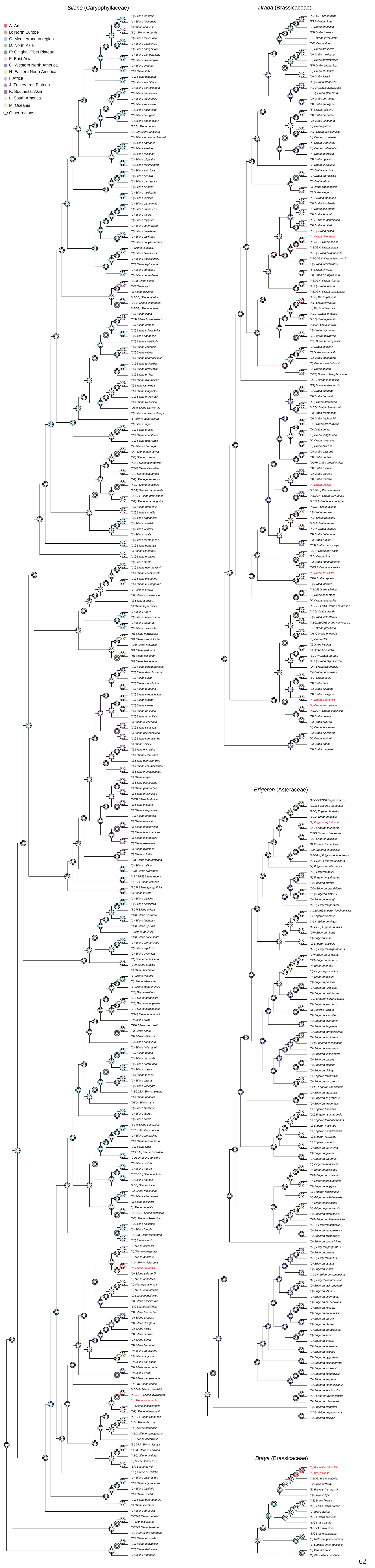

Supplementary Figure 39

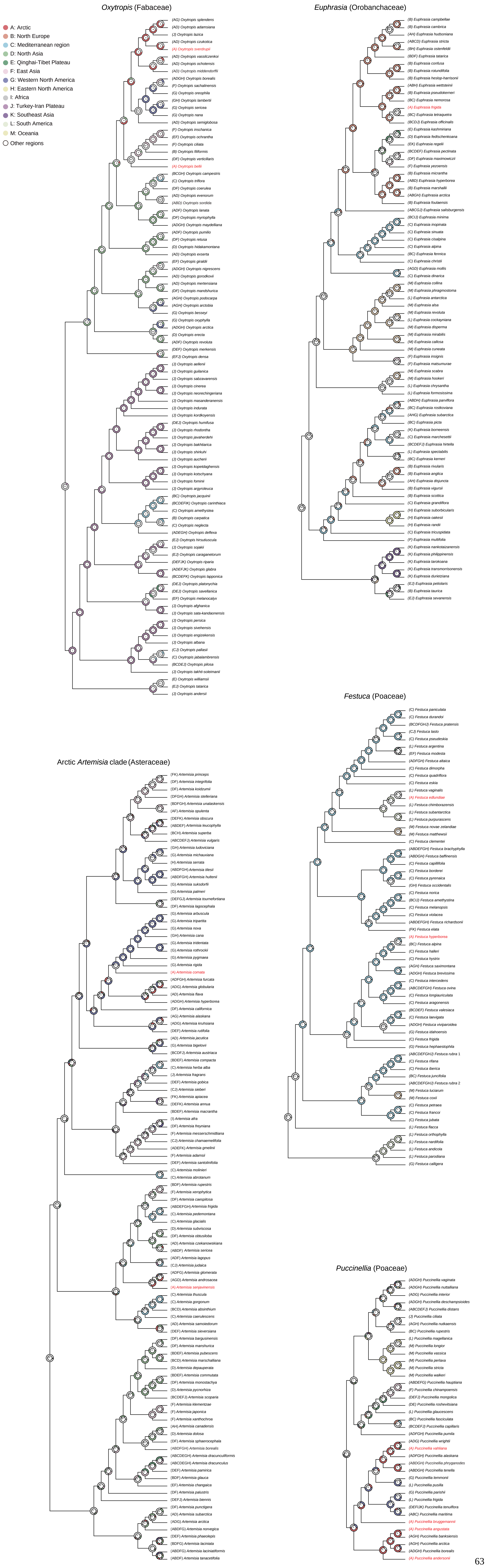

Supplementary Figure 40

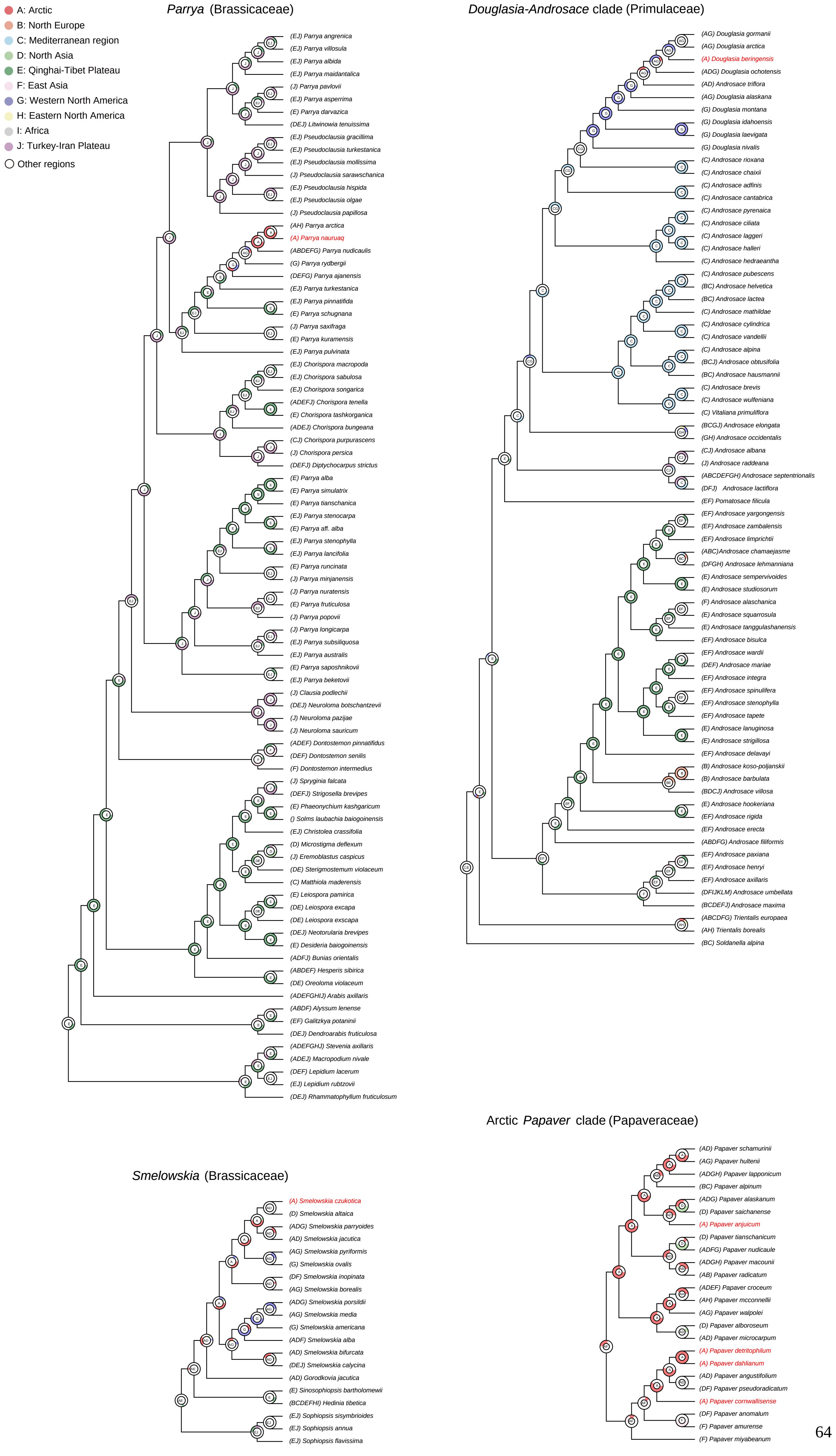

Supplementary Figure 41

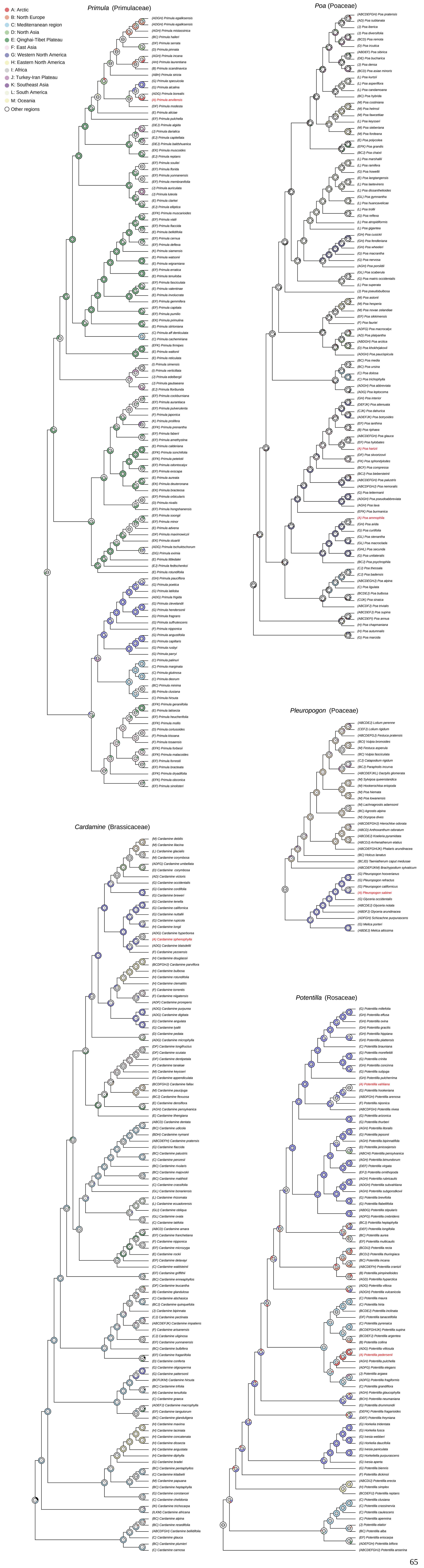

Supplementary Figure 42

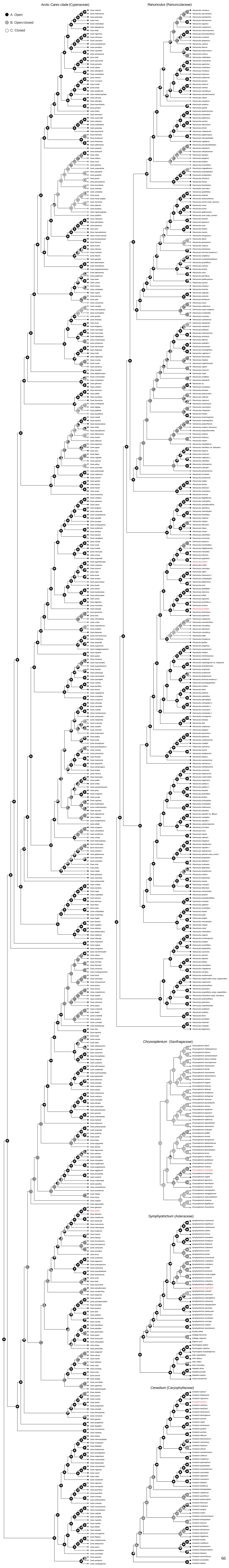

Supplementary Figure 43

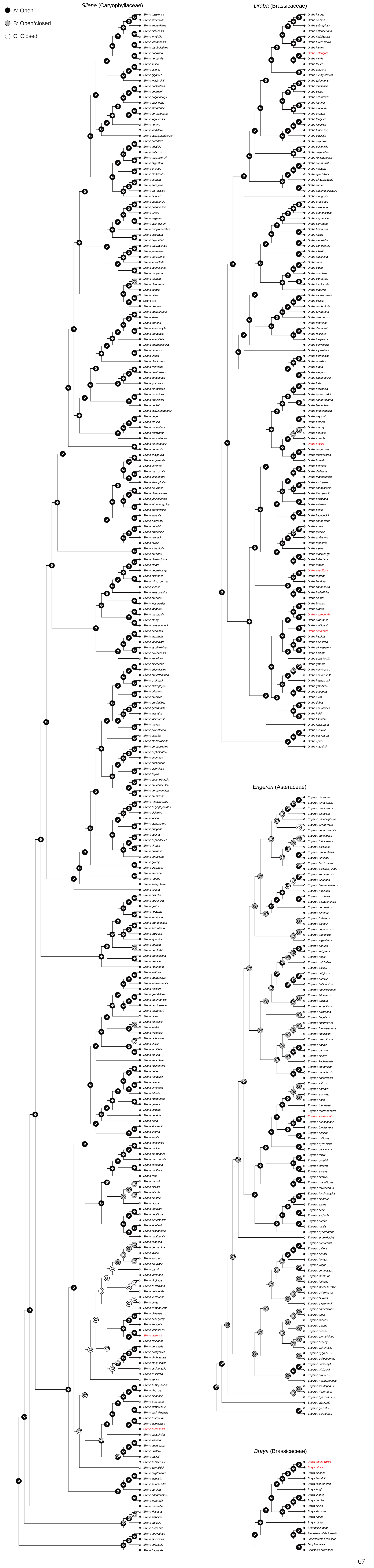

Supplementary Figure 44

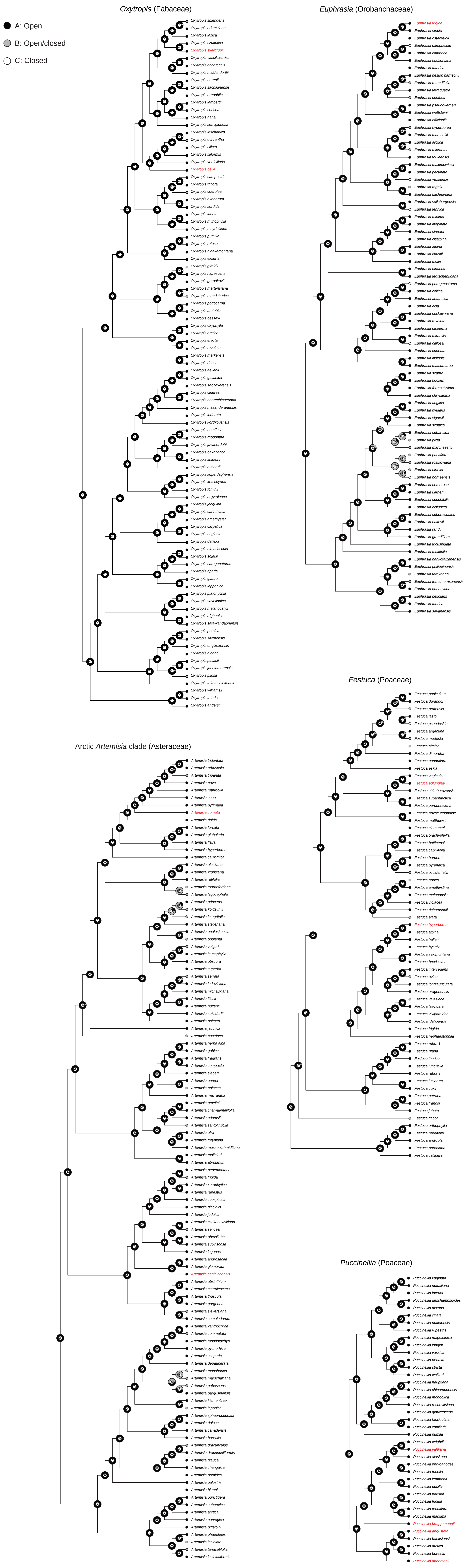

Supplementary Figure 45

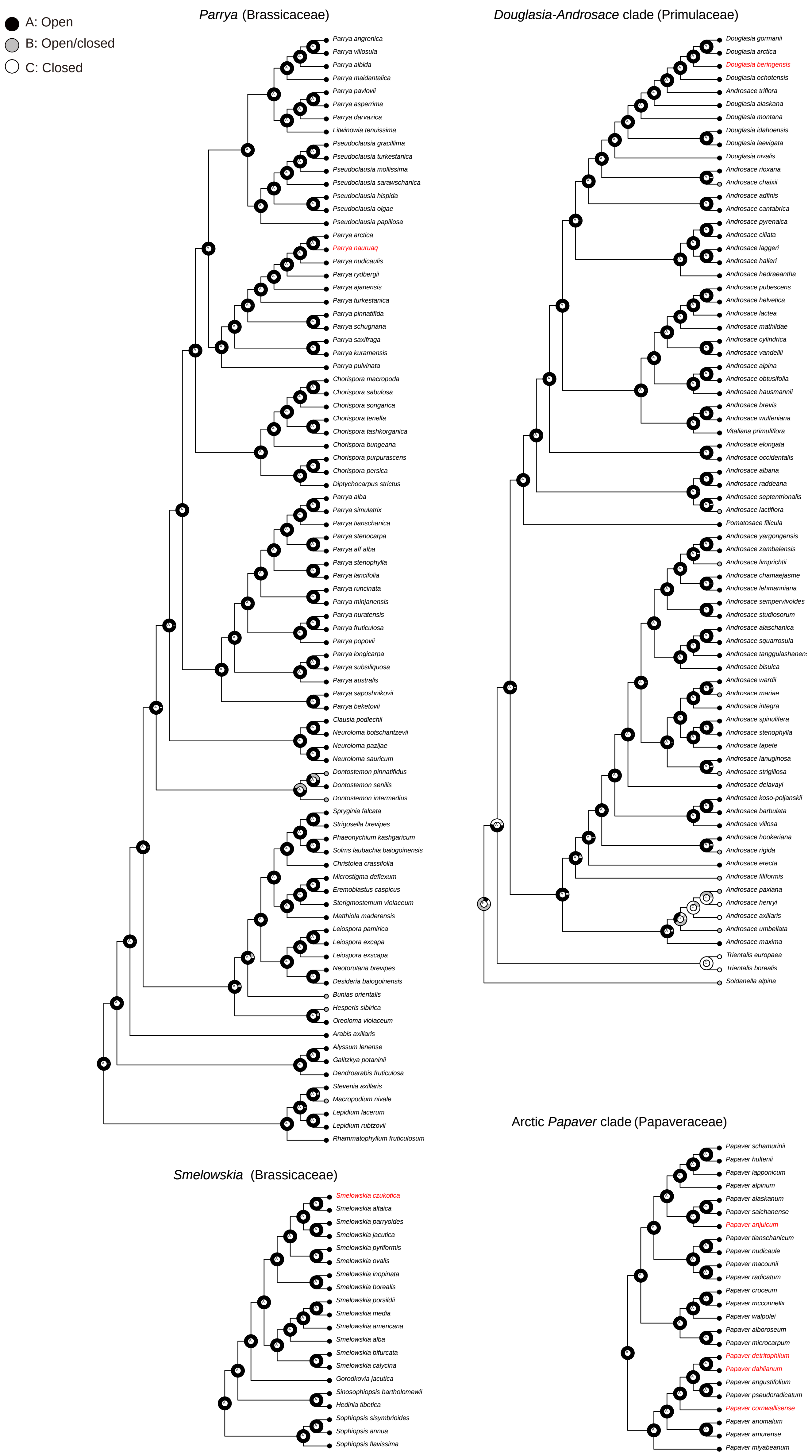

Supplementary Figure 46

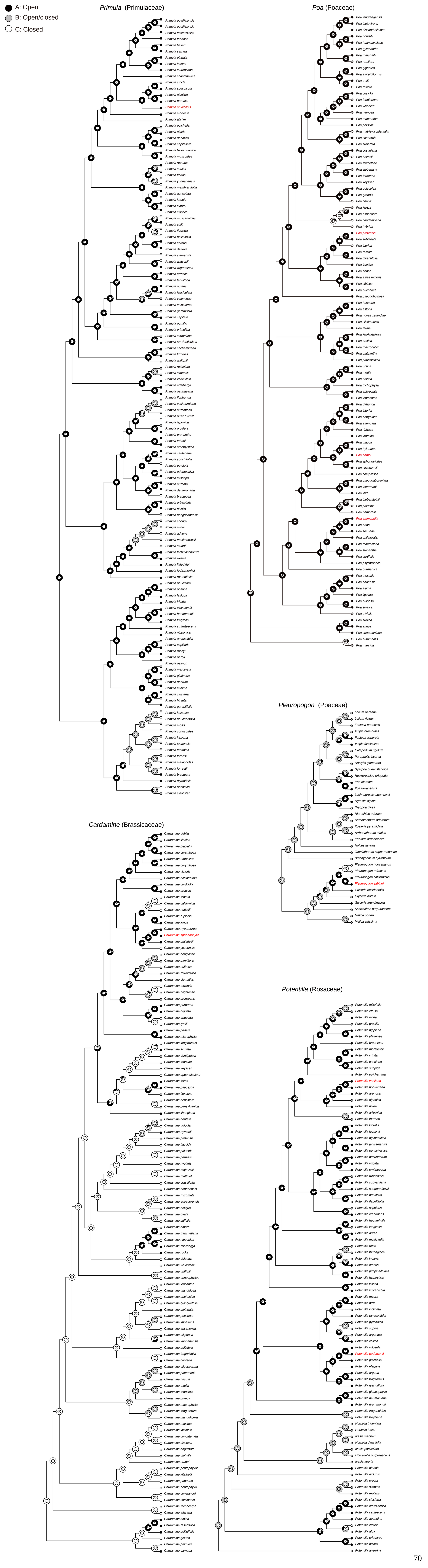

Supplementary Table 1 | Sampling information for the selected 32 clades analyzed in this study

Order	Family	Genus/Clade	Total species		Arctic species		Arctic endemic species		Reference
			Total	Sampled (%)	Total	Sampled (%)	Total	Sampled (%)	
Asterales	Asteraceae	Arctic *Artemisia* clade	51	33 (65%)	33	30 (91%)	2	2 (100%)	2
		Erigeron	400	113 (28%)	23	18 (78%)	1	1 (100%)	40
		Packera	70	20 (29%)	6	2 (33%)	–	–	23
		Symphyotrichum	90	33 (37%)	9	4 (44%)	1	1 (100%)	39
Brassicales	Brassicaceae	*Braya*	12	12 (100%)	7	7 (100%)	2	2 (100%)	41
		Cardamine	200	114 (57%)	17	16 (94%)	1	1 (100%)	41
		Draba	360	133 (37%)	44	37 (84%)	7	5 (71%)	42
		Parrya	40	35 (88%)	3	3 (100%)	1	1 (100%)	26
		Smelowskia	14	14 (100%)	9	9 (100%)	1	1 (100%)	42
Caryophyllales	Caryophyllaceae	*Cerastium*	100	52 (52%)	13	10 (77%)	1	1 (100%)	12
		Silene	700	280 (40%)	19	16 (84%)	1	1 (100%)	44
	Polygonaceae	*Rumex*	150	74 (49%)	23	17 (65%)	–	–	35
Ericales	Ericaceae	*Cassiope*	17	7 (41%)	3	2 (67%)	–	–	9
	Diapensiaceae	*Diapensia*	5	4 (80%)	2	2 (100%)	–	–	15
	Primulaceae	*Douglasia-Androsace* clade	110	68 (62%)	11	9 (82%)	1	1 (100%)	45
		Primula	500	126 (25%)	12	12 (100%)	1	1 (100%)	46
Fabales	Fabaceae	*Astragalus*	3000	158 (5%)	27	17 (63%)	2	0 (0%)	47
		Oxytropis	450	94 (21%)	46	21 (46%)	8	2 (25%)	48
Lamiales	Orobanchaceae	*Euphrasia*	350	80 (23%)	13	11 (85%)	1	1 (100%)	49

		Pedicularis	500	211 (42%)	28	23 (82%)	–	–	50
	Plantaginaceae	*Plantago*	250	51 (20%)	7	7 (100%)	–	–	51
Poales	Cyperaceae	Arctic *Carex* clade	520	288 (55%)	136	114 (84%)	1	1 (100%)	52
		Festuca	500	62 (12%)	16	11 (69%)	3	2 (67%)	21
	Poaceae	*Pleuropogon*	6	4 (67%)	1	1 (100%)	1	1 (100%)	53
		Poa	500	100 (20%)	35	22 (63%)	2	2 (100%)	54
		Puccinellia	200	39 (20%)	32	19 (59%)	11	4 (36%)	55
Ranunculales	Papaveraceae	Arctic *Papaver* clade	30	24 (80%)	24	16 (67%)	14	4 (29%)	56
	Ranunculaceae	*Delphinium*	300	104 (35%)	7	5 (71%)	–	–	57
		Ranunculus	*c.* 600	301 (50%)	33	32 (97%)	2	2 (100%)	58
Rosales	Rosaceae	*Potentilla*	300–400	84 (28%)	44	25 (57%)	6	2 (33%)	59
Saxifragales	Saxifragaceae	*Chrysosplenium*	70	49 (70%)	5	4(80%)	1	1 (100%)	60
		Saxifraga	450–500	134 (*c.* 30%)	31	26 (84%)	2	0 (0%)	61

Supplementary Table 2 | Results of the segmented regression of the increase in the inferred diversification events related to the Arctic over time

Features of the diversification dynamics	Number of change points	BIC	Point estimate of change points (95% confidence interval) (Ma)
MDisE	1	595.26	4.35 (4.50–4.20)
	2	580.25	7.37 (7.71–7.03), 3.31 (3.49–3.13)
	**3**	**385.22**	**7.23 (7.34–7.12), 2.56 (2.61–2.51), 0.63 (0.65–0.61)**
	4	411.98	7.21 (7.34–7.08), 2.84 (2.92–2.76), 1.46 (1.57–1.35), 0.70 (0.73–0.67)
MDivE	1	414.92	4.52 (4.71–4.33)
	2	416.71	6.70 (7.30–6.10), 4.02 (4.31–3.71)
	**3**	**232.71**	**6.25 (6.38–6.12), 2.34 (2.40–2.28), 0.68 (0.70–0.66)**
	4	237.50	9.70 (10.06–9.34), 6.32 (6.45–6.19), 2.31 (2.37–2.25), 0.74 (0.76–0.72)

Note. The most parsimonious model is highlighted in bold. The results under the most parsimonious model are presented in Supplementary Fig. 5. MDisE = maximal number of observed dispersal events per Ma. MDivE = maximal number of observed *in situ* diversification events per Ma.

Supplementary Table 3 | Summary of the uplift and erosion events in the Arctic and its adjacent regions since the mid-late Miocene

Region	Locality	Age (Ma)	Paleoelevation (km)	Modern elevation (km)	Method	References
Greenland	Nuussuaq, West Greenland	11–10, 7–2	*c*. 0	*c*. 2.1	Thermochronology	62
	East Greenland	*c*. 10, *c*. 5	*c*. 0	*c*. 2.0–3.0	Thermochronology	63
	Northeast Greenland	*c*. 15–10	–	–	Sedimentology	64
Svalbard	West Spitsbergen Foldbelt	*c*. 10	*c*. 0	*c*. 1.0	Sedimentology	64
	Central Tertiary Basin	*c*. 10	*c*. 0	*c*. 0.5	Sedimentology	65
Alaska	Central Alaska Range	5.3–0	*c*. 0.2	*c*. 3.0	Fossil, Thermochronology	66, 67
	Mt. McKinley	*c*. 6–5	–	*c*. 6.0	Thermochronology	68
	Yakataga Fm.	*c*. 8.5	–	*c*. 5.0	Sedimentology, Isotope	69
Other Ranges	Barents Shelf	5.3–0	–	–	Sedimentology	70

Supplementary Table 4 | The numbers and median ages (95% confidence intervals) of different biogeographic events inferred in this study

Biogeographic event type	Number	Age of origination of MDisE (Ma)	Age of peak of MDisE (Ma)
From western North America	14	9.75(10.50–6.90)	1.45(3.20–0.80)
From North Asia	3	3.32(3.40–1.60)	0.78(1.80–1.00)
From East Asia	3	2.16(2.40–1.40)	0.65(0.80–0.20)
From North Europe	2	1.32(1.40–1.10)	0.28(0.30–0.02)
From Mediterranean coastal	2	7.52(9.10–2.30)	1.73(2.20–0.30)
From eastern North America	1		
From South America	1		

Supplementary Table 5 | Primers used for amplification and sequencing in this study

Locus	Name	Sequence (5'-3')	Reference
ITS	ITS-1	CCTTATCATTTAGAGGAAGGAG	71
	ITS-4	TCCTCCGCTATTGATATGC	71
*mat*K	matK-AF2	CTTTCAGGARTACATTTATGC	72
	matK-8R2	ACGWGCCAAAGTTCTAGCAC	72
	matK-mF2	AAACAATCTTMTCATTTACG	72
	matK-mR2	AARGGATCCTTGAACAMCCA	72
*rbc*L	rbcL-1F	ATGTCACCACAAACAGAAACT	73
	rbcL-991R	CGGTACCAGCGTGAATATGAT	73
	rbcL-1494R	GATTGGGCCGAGTTAATTAC	73
*atp*B-*rbc*L	ATPBE	GTGGAAACCCCGGGACGAGAAGTAGT	74
	27-mer	ACTTGCTTTAGTTTCTGTTTGTGGTGA	74
*trn*L-*trn*F	c	CGAAATCGGTAGACGCTACG	75
	d	GGGGATAGAGGGACTTGAAC	75
	e	GGTTCAAGTCCCTCTATCCC	75
	f	ATTTGAACTGGTGACACGAG	75

Supplementary Table 6 | Dispersal multipliers per time slice used in ancestral range estimations

Regions	A	B	C	D	E	F	G	H	I	J	K	L	M
Time slice 1 (70-35 Ma)													
A	1	1	0.5	1	0.5	0.5	1	1	0.1	0.5	0.1	0.1	0.01
B		1	1	0.1	0.1	0.1	0.1	0.5	0.5	0.1	0.1	0.01	0.01
C			1	0.1	0.1	0.1	0.1	0.5	1	0.1	0.1	0.01	0.01
D				1	1	1	0.1	0.1	0.5	1	0.5	0.01	0.1
E					1	1	0.1	0.1	0.5	1	1	0.01	0.5
F						1	0.1	0.1	0.1	0.5	1	0.01	0.5
G							1	1	0.01	0.01	0.01	0.01	0.01
H								1	0.1	0.1	0.01	0.1	0.01
I									1	1	0.5	0.1	0.1
J										1	1	0.01	0.5
K											1	0.01	1
L												1	0.1
M													1
Time slice 2 (35-0 Ma)													
A	1	1	0.5	1	0.5	0.5	1	1	0.1	0.5	0.1	0.01	0.01
B		1	1	1	0.5	0.5	0.1	0.5	0.5	1	0.5	0.01	0.01
C			1	0.5	0.5	0.1	0.1	0.5	1	1	0.5	0.01	0.01
D				1	1	1	0.5	0.1	0.5	1	0.5	0.01	0.1
E					1	1	0.5	0.1	0.5	1	1	0.01	0.5
F						1	0.5	0.1	0.1	0.5	1	0.01	0.5
G							1	1	0.01	0.01	0.01	0.1	0.01
H								1	0.1	0.1	0.01	0.1	0.01
I									1	1	0.5	0.1	0.1
J										1	1	0.01	0.5
K											1	0.01	1
L												1	0.1
M													1

Note. A: Arctic, B: North Europe, C: Mediterranean region, D: North Asia, E: Qinghai-Tibet Plateau, F: East Asia, G: western North America, H: eastern North America, I: Africa, J: Turkey-Iran Plateau, K: Southeast Asia, L: South America, M: Oceania. p = 1.0 (dispersal between adjacent areas), p = 0.5 (dispersal across one area), p = 0.1 (dispersal across two areas), and p = 0.01 (very unlikely dispersal events). Time slice 1 (70–35 Ma). During this time frame either the North Atlantic migration pathways, the Thulean Route (55–57 Ma) connecting Europe and North America, or the Bering Land Bridge (BLB; 66–65 Ma, 58–3.5 Ma) connecting northern Asia and North America allowed for biotic interchange between Northern

Hemisphere landmasses[76] while the Turgai Strait disconnected Europe and Asia[77,78]. Time slice 2 (35–0 Ma). 35–0 Ma correspond roughly to the time frame after the southern QTP had reached significant elevation and the Turgai Strait had closed. The North Atlantic migration pathways were no longer connecting Europe and North America but the BLB allowed for continuous interchange[76].

Supplementary References

1. Chase, M. W. et al. An update of the Angiosperm Phylogeny Group classification for the orders and families of flowering plants: APG IV. *Bot. J. Linn. Soc.* **181,** 1–20 (2016).
2. Tkach, N., Hoffmann, M. H., Röser, M. & von Hagen, K. B. Temporal patterns of evolution in the Arctic explored in *Artemisia* L. (Asteraceae) lineages of different age. *Plant Ecol. Divers.* **1,** 161–169 (2008).
3. Azani, N., Bruneau, A., Wojciechowski, M. F. & Zarre, S. Miocene climate change as a driving force for multiple origins of annual species in *Astragalus* (Fabaceae, Papilionoideae). *Mol. Phylogenet. Evol.* **137,** 210–221 (2019).
4. Chen, H., German, D. A., Al-Shehbaz, I. A., Yue, J. & Sun, H. Phylogeny of *Euclidieae* (Brassicaceae) based on plastome and nuclear ribosomal DNA data. *Mol. Phylogenet. Evol.* **153,** 106940 (2020).
5. Carlsen, T., Bleeker, W., Hurka, H., Elven, R. & Brochmann, C. Biogeography and phylogeny of *Cardamine* (Brassicaceae). *Ann. Mo. Bot. Gard.* **96,** 215–236 (2009).
6. Guo, X. et al. Plastome phylogeny and early diversification of Brassicaceae. *BMC Genomics* **18,** 176 (2017).
7. Martín-Bravo, S. et al. A tale of worldwide success: Behind the scenes of *Carex* (Cyperaceae) biogeography and diversification. *J. Syst. Evol.* **57,** 695–718 (2019).
8. Jiménez-Mejías, P. et al. A commented synopsis of the pre-Pleistocene fossil record of *Carex* (Cyperaceae). *Bot. Rev.* **82,** 258–345 (2016).
9. Hou, Y. et al. RAD-seq data point to a northern origin of the arctic-alpine genus *Cassiope* (Ericaceae). *Mol. Phylogenet. Evol.* **95,** 152–160 (2016a).
10. Gizaw, A. et al. Colonization and diversification in the African 'sky islands': Insights from fossil-calibrated molecular dating of *Lychnis* (Caryophyllaceae). *New Phytol.* **211,** 719–734 (2016).
11. Marincovich, L. & Gladenkov, A. Y. Evidence for an early opening of the Bering Strait. *Nature* **397,** 149–151 (1999).
12. Scheen, A. C. et al. Northern hemisphere biogeography of *Cerastium* (Caryophyllaceae): Insights from phylogenetic analysis of noncoding plastidnucleotide sequences. *Am. J. Bot.* **91,** 943–952 (2004).
13. Deng, J. B. et al. Phylogeny, divergence times and historical biogeography of the angiosperm family Saxifragaceae. *Mol. Phylogenet. Evol.* **83,** 86–98 (2015).
14. Jabbour, F. & Renner, S. S. A phylogeny of Delphinieae (Ranunculaceae) shows that *Aconitum* is nested within *Delphinium* and that late Miocene transitions to long life cycles in the Himalayas and Southwest China coincide with bursts in diversification. *Mol. Phylogenet. Evol.* **62,** 928–942 (2012).
15. Hou, Y., Bjorå, C. S., Ikeda, H., Brochmann, C. & Popp, M. From the north into the Himalayan-Hengduan Mountains: Fossil-calibrated phylogenetic and biogeographical inference in the arctic-alpine genus *Diapensia* (Diapensiaceae). *J. Biogeogr.* **43,** 1502–1513 (2016b).
16. Boucher, F. C. et al. Reconstructing the origins of high-alpine niches and

cushion life form in the genus *Androsace* S.L. (Primulaceae). *Evolution* **66,** 1255–1268 (2012).

17. Jordon-Thaden, I. E, Hase, I., Al-Shehbaz, I. A. & Koch, M. A. Molecular phylogeny and systematics of the genus *Draba* (Brassicaceae) and identification of its most closely related genera. *Mol. Phylogenet. Evol.* **55,** 524–540 (2010).
18. Panero, J. L. & Crozier, B. S. Macroevolutionary dynamics in the early diversification of Asteraceae. *Mol. Phylogenet. Evol.* **99,** 116–132 (2016).
19. Farhani,T., Kazempour-Osaloo, S., Zare-Maivan, H. & Mozaffarian, V. Evolutionary history of the tribe Astereae in the Flora Iranica area: Systematic implications. *Phytotaxa* **379,** 95–117 (2018).
20. Gussarova, G., Popp, M., Vitek, E. & Brochmann, C. Molecular phylogeny and biogeography of the bipolar *Euphrasia* (Orobanchaceae): Recent radiations in an old genus. *Mol. Phylogenet. Evol.* **48,** 444–460 (2008).
21. Inda, L. A., Segarra-Moragues, J. G., Müller, J., Peterson, P. M. & Catalán, P. Dated historical biogeography of the temperate *Loliinae* (Poaceae, Pooideae) grasses in the northern and southern hemispheres. *Mol. Phylogenet. Evol.* **46,** 932–957 (2008).
22. Shavvon, R. S. et al. Increasing phylogenetic support for explosively radiating taxa: The promise of high-throughput sequencing for *Oxytropis* (Fabaceae). *J. Syst. Evol.* **55,** 385–404 (2017).
23. Bain, J. F. & Golden, J. L. A phylogeny of Packera (Senecioneae; asteraceae) based on internal transcribed spacer region sequence data and a broad sampling of outgroups. *Mol. Phylogenet. Evol.* **16,** 331–338 (2000).
24. Mandel, J. R. et al. A fully resolved backbone phylogeny reveals numerous dispersals and explosive diversifications throughout the history of Asteraceae. *Proc. Natl. Acad. Sci. USA* **116,** 14083–14088 (2019).
25. Peng, H. W. et al. A complete genus-level phylogeny reveals the Cretaceous biogeographic diversification of the poppy family. *Mol. Phylogenet. Evol.* **181,** 107712 (2023).
26. German, D. A., Grant, J. R., Lysak, M. A. & Al-Shehbaz, I. A. Molecular phylogeny and systematics of the tribe Chorisporeae (Brassicaceae). *Plant Syst. Evol.* **294,** 65–86 (2011).
27. Yu, W. et al. The hemiparasitic plant *Phtheirospermum* (Orobanchaceae) is polyphyletic and contains cryptic species in the Hengduan Mountains of southwest China. *Front. Plant Sci.* **9,** (2018).
28. Rønsted, N., Chase, M. W., Albach, D. C. & Bello, M. A. Phylogenetic relationships within *Plantago* (Plantaginaceae): Evidence from nuclear ribosomal ITS and plastid *trn*L-F sequence data. *Bot. J. Linn. Soc.* **139,** 323–338 (2002).
29. Bouchenak-Khelladi, Y., Verboom, G. A., Savolainen, V. & Hodkinson, T. R. Biogeography of the grasses (Poaceae): A phylogenetic approach to reveal evolutionary history in geographical space and geological time. *Bot. J. Linn. Soc.* **162,** 543–557 (2010).
30. Dobeš, C. & Paule, J. A comprehensive chloroplast DNA-based phylogeny of

the genus *Potentilla* (Rosaceae): Implications for its geographic origin, phylogeography and generic circumscription. *Mol. Phylogenet. Evol.* **56,** 156–175 (2010).

31. Mast, A. R., Kelso, S. & Conti, E. Are any primroses (*Primula*) primitively monomorphic? *New Phytol.* **171,** 605–616 (2006).
32. de Vos, J. M., Hughes, C. E., Schneeweiss, G. M., Moore, B. R. & Conti, E. Heterostyly accelerates diversification via reduced extinction in primroses. *Proc. R. Soc. B.* **281,** 20140075 (2014).
33. Emadzade, K. & Hörandl, E. Northern hemisphere origin, transoceanic dispersal, and diversification of *Ranunculeae* DC. (Ranunculaceae) in the Cenozoic. *J. Biogeogr.* **38,** 517–530 (2011).
34. Schuster, T. M., Setaro, S. D. & Kron, K. A. Age estimates for the buckwheat family Polygonaceae based on sequence data calibrated by fossils and with a focus on the amphi-Pacific Muehlenbeckia. *PLoS One* **8,** e61261 (2013).
35. Ebersbach, J. et al. In and out of the Qinghai-Tibet Plateau: Divergence time estimation and historical biogeography of the large arctic-alpine genus *Saxifraga* L. *J. Biogeogr.* **44,** 900–910 (2017).
36. Sloan, D. B., Oxelman, B., Rautenberg, A. & Taylor, D. R. Phylogenetic analysis of mitochondrial substitution rate variation in the angiosperm tribe Sileneae. *BMC Evol. Biol.* **9,** 260 (2009).
37. Carlsen, T., Elven, R. & Brochmann, C. The evolutionary history of Beringian *Smelowskia* (Brassicaceae) inferred from combined microsatellite and DNA sequence data. *Taxon* **59,** 427–438 (2010).
38. Couvreur, T. L. P. et al. Molecular phylogenetics, temporal diversification, and principles of evolution in the mustard family (Brassicaceae). *Mol. Biol. Evol.* **27,** 55–71 (2010).
39. Vaezi, J. & Brouillet, L. Phylogenetic relationships among diploid species of *Symphyotrichum* (Asteraceae: Astereae) based on two nuclear markers, ITS and GAPDH. *Mol. Phylogenet. Evol.* **5,** 540–53 (2009).
40. Noyes, R. D. Biogeographical and evolutionary insights on *Erigeron* and allies (Asteraceae) from ITS sequence data. *Plant Syst. Evol.* **220,** 93–114. (2000)
41. Warwick, S. I., Al-Shehbaz, I. A., Sauder, C., Harris, J. G. & Koch, M. Phylogeny of *Braya* and *Neotorularia* (Brassicaceae) based on nuclear ribosomal internal transcribed spacer and chloroplast *trn*L intron sequences. *Can. J. Bot.* **82,** 376–392 (2004a).
42. Warwick, S. I., Francis, A. & Al-Shehbaz, I. A. Brassicaceae: Species checklist and database on CD-Rom. *Plant Syst. Evol.* **259,** 249–258 (2006).
43. Warwick, S. I., Al-Shehbaz, I. A., Sauder, C. A., Murray, D. F. & Mummenhoff, K. Phylogeny of *Smelowskia* and related genera (Brassicaceae) based on nuclear ITS DNA and chloroplast *trn*L intron DNA sequences. *Ann. Mo. Bot. Gard.* **91,** 99–123 (2004b).
44. Greuter, W. *Silene* (Caryophyllaceae) in Greece: A subgeneric and sectional classification. *Taxon* **44,** 543–581 (1995).
45. Schneeweiss, G. M., Schönswetter, P., Kelso, S. & Niklfeld, H. Complex

biogeographic patterns in *Androsace* (Primulaceae) and related genera: Evidence from phylogenetic analyses of nuclear internal transcribed spacer and plastid *trn*L-F sequences. *Syst. Biol.* **53,** 856–876 (2004).

46. Ren, G., Conti, E. & Salamin, N. Phylogeny and biogeography of *Primula* sect. Armerina: Implications for plant evolution under climate change and the uplift of the Qinghai-Tibet Plateau. *BMC Evol. Biol.* **15,** 161–161 (2015).
47. Wojciechowski, M. F. Sanderson, M. J. & Hu, J. Evidence on the monophyly of *Astragalus* (Fabaceae) and its major subgroups based on nuclear ribosomal DNA ITS and chloroplast DNA *trn*L intron data. *Syst. Bot.* **24,** 409–437 (1999).
48. Malyshev, L. I. Phenetics of the subgenera and sections in the genus *Oxytropis* DC. (Fabaceae) bearing on ecology and phylogeny. *Contemp. Probl. Ecol.* **1,** 440–444 (2008).
49. Fisher, E. in *The Families and Genera of Vascular Plants* Vol. 7 (ed Kadereit, J. W.) 333–432 (Springer, Berlin, 2004).
50. Mill, R. R. Notes relating to the flora of Bhutan: XLIII. Scrophulariaceae (Pedicularis). *Edinb. J. Bot.* **58,** 57–98 (2001).
51. Shipunov, A. et al. How to map a plantain: Phylogeny of the diverse Plantagineae (Lamiales). Preprint at https://www.biorxiv.org/content/10.1101/2020.07.31.230813v1 (2020).
52. Hoffmann, M. H., Gebauer, S. & von Rozycki, T. Assembly of the Arctic flora: Highly parallel and recurrent patterns in sedges (*Carex*). *Am. J. Bot.* **104,** 1334–1343 (2017).
53. Soreng, R. J. et al. A worldwide phylogenetic classification of the Poaceae (Gramineae): Phylogenetic classification of the grasses. *J. Syst. Evol.* **53,** 117–137 (2015).
54. Birch, J. L., Cantrill, D. J., Walsh, N. G. & Murphy, D. J. Phylogenetic investigation and divergence dating of *Poa* (Poaceae, tribe Poeae) in the Australasian region. *Bot. J. Linn. Soc.* **175,** 523–552 (2014).
55. Consaul, L. L., Gillespie, L. J. & Waterway, M. J. Evolution and polyploid origins in North American Arctic *Puccinellia* (Poaceae) based on nuclear ribosomal spacer and chloroplast DNA sequences. *Am. J. Bot.* **97,** 324–336 (2010).
56. Kadereit, J. W., Preston, C. & Valtueña, F. J. Is Welsh Poppy, Meconopsis cambrica (L.) Vig. (Papaveraceae), truly a Meconopsis? *New Zeal. J. Bot.* **1,** 80–88 (2011).
57. Wang, W. T. & Warnock, M. J. in *Flora of China* Vol. 6 (eds Wu, Z.Y. & Raven, P. H.) 223–237 (Science Press, Missouri Botanical Garden, Beijing, St. Louis, 2001).
58. Tamura, M. in *Angiospermae: Ordnung Ranunculales. Fam. Ranunculaceae. II. Systematic Part* (ed Hiepko, P.) 223–519 (Duncker und Humblot, Berlin, 1995).
59. Soják, J. *Potentilla* L. s.1. (Rosaceae) in Flora Europae Orientalis (Notes on *Potentilla* XVIII). *Candollea* **60,** 59–78 (2005).
60. Kim, Y. et al. A new species of *Chrysosplenium* (Saxifragaceae) from Northeastern China. *PhytoKeys* **135,** 39–47 (2019).

61. Zhmylev, P. Y. *Genus Saxifraga L.* (Saxifragaceae)*: Biomorphology, Systematics and Evolution of the Life Forms* (Moscow State University, Russian, 2004).
62. Japsen, P., Green, P. F. & Chalmers, J. A. Separation of palaeogene and Neogene uplift on Nuussuaq, West Greenland. *J. Geol. Soc.* **162,** 299–314 (2005).
63. Japsen, P., Green, P. F., Bonow, J. M., Nielsen, T. F. & Chalmers, J. A. From volcanic plains to glaciated peaks: Burial, uplift and exhumation history of southern East Greenland after opening of the NE Atlantic. *Global Planet. Change* **116,** 91–114 (2014).
64. Døssing, A. et al. Miocene uplift of the NE Greenland margin linked to plate tectonics: Seismic evidence from the Greenland Fracture Zone, NE Atlantic. *Tectonics* **35,** 257–282 (2016).
65. Dörr, N., Clift, P. D., Lisker, F. & Spiegel, C. Why is Svalbard an island? Evidence for two-stage uplift, magmatic underplating, and mantle thermal anomalies. *Tectonics* **32,** 473–486 (2013).
66. Wahrhaftig, C., Wolfe, J. A., Leopold, E. B. & Lanphere, M. A. The coal-bearing group in the Nenana coal field, Alaska. *United States Geol. Surv. Bull.* 1274–D (1969).
67. Fitzgerald, P. G., Sorkhabi, R. B., Redfield, T. F. & Stump, E. Uplift and denudation of the central Alaska Range: A case study in the use of apatite fission track thermochronology to determine absolute uplift parameters. *J. Geophys. Res.* **100,** 20175–20191 (1995).
68. Plafker, G., Naeser, C. W., Zimmermann, R. A., Lull, J. S. & Hudson, T. Cenozoic uplift history of the Mount McKinley area in the central Alaska Range based on fission-track dating. *U.S. Geological Survey* **2041,** 202–212 (1991).
69. Lagoe, M. B., Eyles, C. H., Eyles, N. & Hale, C. J. Timing of late Cenozoic tidewater glaciation in the far North Pacific. *Geol. Soc. Am. Bull.* **105,** 1542–1560 (1993).
70. Lasabuda, A. P. E. et al. Cenozoic uplift and erosion of the Norwegian Barents Shelf-A review. *Earth-Sci. Rev.* **217,** 103609 (2021).
71. White, T. J., Bruns, T., Lee, S. & Taylor, J. in *PCR Protocols: A guide to methods and applications* (eds Innis, M., Gelfand, D., Sninsky, J. & White, T.) 315–322 (Academic Press, San Diego, 1990).
72. Wang, W., Chen, Z. D., Liu, Y., Li, R. Q. & Li, J. H. Phylogenetic and biogeographic diversification of Berberidaceae in the northern hemisphere. *Syst. Bot.* **32,** 731–742 (2007).
73. Chen, Z. D. et al. Systematic position of the Rhoipteleaceae: Evidence from nucleotide sequences of *rbc*L gene. *Acta Phytotaxon. Sin.* **36,** 1–7 (1998).
74. Hodges, S. A. & Arnold, M. L. Columbines: A geographically widespread species flock. *Proc. Natl. Acad. Sci. USA* **91,** 5129–5132 (1994).
75. Taberlet, P., Gielly, L., Pautou, G. & Bouvet, J. Universal primers for amplification of three non-coding regions of chloroplast DNA. *Plant Mol.Biol.* **17,** 1105–1109 (1991).

76. Brikiatis, L. The De Geer, Thulean and Beringia routes: Key concepts for understanding early Cenozoic biogeography. *J. Biogeogr.* **41,** 1036–1054 (2014).
77. Rögl, F. Palaeogeographic considerations for Mediterranean and Paratethys Seaways (Oligocene to Miocene). *Ann. Naturhist. Mus. Wien* **99A,** 279–310 (1998).
78. Sanmartín, I. Enghoff, H. & Ronquist, F. Patterns of animal dispersal, vicariance and diversification in the Holarctic. *Biol. J. Linn. Soc.* **73,** 345–390 (2001).
