# Supplementary material for: Evolutionary history of the Arctic flora
Source: Nat Commun. 2023 Jul 18;14:4021. doi: 10.1038/s41467-023-39555-6 (PMC10354081; doi:10.1038/s41467-023-39555-6)
Supplement: Supplementary file 3 — Description of Additional Supplementary Files [file 41467_2023_39555_MOESM3_ESM.pdf]

Description of Additional Supplementary Files

File Name: Supplementary Data 1

Description: Inferred dispersal events and *in situ* diversification events related to the Arctic summarized from Supplementary Figs. 29–35.

File Name: Supplementary Data 2

Description: Estimated ages (Ma) of 548 Arctic species.

File Name: Supplementary Data 3

Description: Species and GenBank accession numbers used in this study.
