# Supplementary material for: Evolutionary history of the Arctic flora
Source: Nat Commun. 2023 Jul 18;14:4021. doi: 10.1038/s41467-023-39555-6 (PMC10354081; doi:10.1038/s41467-023-39555-6)
Supplement: Supplementary file 7 — Reporting Summary [file 41467_2023_39555_MOESM7_ESM.pdf]

Corresponding author(s): Wei Wang, Fu-Cai Xia, Pamela S. Soltis, Douglas E. Soltis

Last updated by author(s): Jun 9, 2023

Reporting Summary

Nature Portfolio wishes to improve the reproducibility of the work that we publish. This form provides structure for consistency and transparency in reporting. For further information on Nature Portfolio policies, see our Editorial Policies and the Editorial Policy Checklist.

Statistics

For all statistical analyses, confirm that the following items are present in the figure legend, table legend, main text, or Methods section.

n/a	Confirmed	
☐	☒	The exact sample size (*n*) for each experimental group/condition, given as a discrete number and unit of measurement
☒	☐	A statement on whether measurements were taken from distinct samples or whether the same sample was measured repeatedly
☒	☐	The statistical test(s) used AND whether they are one- or two-sided<br>*Only common tests should be described solely by name; describe more complex techniques in the Methods section.*
☒	☐	A description of all covariates tested
☐	☒	A description of any assumptions or corrections, such as tests of normality and adjustment for multiple comparisons
☐	☒	A full description of the statistical parameters including central tendency (e.g. means) or other basic estimates (e.g. regression coefficient) AND variation (e.g. standard deviation) or associated estimates of uncertainty (e.g. confidence intervals)
☒	☐	For null hypothesis testing, the test statistic (e.g. *F*, *t*, *r*) with confidence intervals, effect sizes, degrees of freedom and *P* value noted<br>*Give P values as exact values whenever suitable.*
☐	☒	For Bayesian analysis, information on the choice of priors and Markov chain Monte Carlo settings
☐	☒	For hierarchical and complex designs, identification of the appropriate level for tests and full reporting of outcomes
☒	☐	Estimates of effect sizes (e.g. Cohen's *d*, Pearson's *r*), indicating how they were calculated

Our web collection on statistics for biologists contains articles on many of the points above.

Software and code

Policy information about availability of computer code

Data collection	N/A
Data analysis	Softwares used for data analyses include R 4.2.2., MAFFT v.7.037, Geneious v.9.1.4, IQ-TREE v.2.1.2, BEAST v.1.8.4, Tracer v.1.7.2, and RASP 4.2.

For manuscripts utilizing custom algorithms or software that are central to the research but not yet described in published literature, software must be made available to editors and reviewers. We strongly encourage code deposition in a community repository (e.g. GitHub). See the Nature Portfolio guidelines for submitting code & software for further information.

Data

Policy information about availability of data

All manuscripts must include a data availability statement. This statement should provide the following information, where applicable:

- Accession codes, unique identifiers, or web links for publicly available datasets
- A description of any restrictions on data availability
- For clinical datasets or third party data, please ensure that the statement adheres to our policy

Newly obtained sequences have been deposited in GenBank and their accession numbers are provided in Supplementary Data 3. Alignments and timetrees for the 32 clades investigated are available in the Zenodo database at https://doi.org/10.5281/zenodo.7868007.

Human research participants

Policy information about studies involving human research participants and Sex and Gender in Research.

Reporting on sex and gender	N/A
Population characteristics	N/A
Recruitment	N/A
Ethics oversight	N/A

Note that full information on the approval of the study protocol must also be provided in the manuscript.

Field-specific reporting

Please select the one below that is the best fit for your research. If you are not sure, read the appropriate sections before making your selection.

☐ Life sciences ☐ Behavioural & social sciences ☒ Ecological, evolutionary & environmental sciences

For a reference copy of the document with all sections, see nature.com/documents/nr-reporting-summary-flat.pdf

Ecological, evolutionary & environmental sciences study design

All studies must disclose on these points even when the disclosure is negative.

Study description	This study describes insights into the origin and evolutionary dynamics of the Arctic flora. We selected 32 clades and reconstructed a time-calibrated phylogeny for each of these clades. We then compiled credibility intervals of dispersal and in situ diversification times and calculated their maximal number of observed diversification events, respectively. We also inferred the ancestral habitat states for each clade for the Arctic endemic species to determine whether the immigrants of the Arctic originated from pre-adapted lineages or were subjected to habitat shifts.
Research sample	We selected 32 angiosperm clades that together encompass 3,626 species, of which 548 are distributed in the Arctic and 40 are restricted to the Arctic.
Sampling strategy	We selected 32 angiosperm clades that contain Arctic and non-Arctic species and have sufficient molecular data available to infer a time-calibrated phylogeny covering the major taxonomic and geographical diversity of each clade. These selected taxa belong to 10 orders and 16 families (following APG IV) across the angiosperm tree of life. We first conducted maximum likelihood analysis in IQ-TREE v.2.1.2 and removed the strongly conflicting taxa (> 70% bootstrap values for different placements) between the plastid and nuclear trees. After removing sixty-eight species, all of which are non-Arctic (Supplementary Table 7), a total of 3,588 species was used for subsequent analyses.
Data collection	The DNA data were generated on ABI Prism 3730xl DNA sequencers or downloaded from GenBank.
Timing and spatial scale	We collected 185 new sequences from 50 species between 2017 and 2020, and downloaded the remaining sequences from GenBank (through August 2022).
Data exclusions	Specimens with low levels of successful sequencing were excluded from analyses, as well as some individuals that appeared to be hybrids between two species.
Reproducibility	We have provided all data and results of this study in GenBank, Zenodo, and Supplementary files. Further, we provided detailed methodology for analyses in the Methods section. We therefore believe that all results of this study can be reproduced.
Randomization	We performed phylogenetic analyses based on the taxon sampling and molecular sampling outlined, applying models of nucleotide evolution to infer phylogenetic trees using maximum likelihood methods. Phylogenetic methods are fundamentally different from a standard statistical experimental design that requires randomization.
Blinding	There was no blinding because there are no participants who may be influenced by the treatments.

Did the study involve field work? ☒ Yes ☐ No

Field work, collection and transport

Field conditions	Field conditions are not related with our study, in which we only collected leaves to do DNA extraction and sequencing.
Location	Locations of all specimens collected are detailed in Supplemental Figures 4 and 6.
Access & import/export	All dried plant collections included in our analyses were collected and transported in compliance with national and international regulations.
Disturbance	There was no disturbance caused by this study. Our collecting only includes one leaf for one species.

Reporting for specific materials, systems and methods

We require information from authors about some types of materials, experimental systems and methods used in many studies. Here, indicate whether each material, system or method listed is relevant to your study. If you are not sure if a list item applies to your research, read the appropriate section before selecting a response.

Materials & experimental systems

n/a	Involved in the study
☒	☐ Antibodies
☒	☐ Eukaryotic cell lines
☒	☐ Palaeontology and archaeology
☒	☐ Animals and other organisms
☒	☐ Clinical data
☒	☐ Dual use research of concern

Methods

n/a	Involved in the study
☒	☐ ChIP-seq
☒	☐ Flow cytometry
☒	☐ MRI-based neuroimaging
